# Design, Synthesis, Anticancer Activity, and Solid Lipid Nanoparticle Formulation of Indole- and Benzimidazole-Based Compounds as Pro-Apoptotic Agents Targeting Bcl-2 Protein

**DOI:** 10.3390/ph14020113

**Published:** 2021-02-01

**Authors:** Manar I. Nagy, Khaled M. Darwish, Safaa M. Kishk, Mohamed A. Tantawy, Ali M. Nasr, Mona Qushawy, Shady A. Swidan, Samia M. Mostafa, Ismail Salama

**Affiliations:** 1 Department of Medicinal Chemistry, Faculty of Pharmacy, Suez Canal University, Ismailia 41522, Egypt; manar.nagy@pharm.suez.edu.eg (M.I.N.); khaled_darwish@pharm.suez.edu.eg (K.M.D.); safaa_keshk@pharm.suez.edu.eg (S.M.K.); samiamostafa2010@hotmail.com (S.M.M.); 2 National Research Center, Hormones Department, Medical Research Division, Dokki, Giza 12622, Egypt; mohamed_tantawy@daad-alumni.de; 3 Department of Pharmaceutics, Faculty of Pharmacy, Port Said University, Port Said 42526, Egypt; a.nasr@pharm.psu.edu.eg; 4 Department of Pharmaceutics, Faculty of Pharmacy, Sinai University, Alarish, North Sinai 45511, Egypt; mqushawy@ut.edu.sa; 5 Department of Pharmaceutics, Faculty of Pharmacy, University of Tabuk, Tabuk 71491, Saudi Arabia; 6 Department of Pharmaceutics, Faculty of Pharmacy, The British University in Egypt, El-Sherouk City, Cairo 11837, Egypt; shady.swidan@bue.edu.eg; 7 The Center for Drug Research and Development (CDRD), Faculty of Pharmacy, The British University in Egypt, El-Sherouk City, Cairo 11837, Egypt

**Keywords:** Bcl-2 inhibitors, Indole-based analogues, benzimidazole, MTT cytotoxic assay, cell cycle analysis, DNA fragmentation, ELISA, docking, solid/lipid nanoparticles

## Abstract

Cancer is a multifactorial disease necessitating identification of novel targets for its treatment. Inhibition of Bcl-2 for triggered pro-apoptotic signaling is considered a promising strategy for cancer treatment. Within the current work, we aimed to design and synthesize a new series of benzimidazole- and indole-based derivatives as inhibitors of Bcl-2 protein. The market pan-Bcl-2 inhibitor, obatoclax, was the lead framework compound for adopted structural modifications. The obatoclax’s pyrrolylmethine linker was replaced with straight alkylamine or carboxyhydrazine methylene linkers providing the new compounds. This strategy permitted improved structural flexibility of synthesized compounds adopting favored maneuvers for better fitting at the Bcl-2 major hydrophobic pocket. Anti-cancer activity of the synthesized compounds was further investigated through MTT-cytotoxic assay, cell cycle analysis, RT-PCR, ELISA and DNA fragmentation. Cytotoxic results showed compounds **8a**, **8b** and **8c** with promising cytotoxicity against MDA-MB-231/breast cancer cells (IC_50_ = 12.69 ± 0.84 to 12.83 ± 3.50 µM), while **8a** and **8c** depicted noticeable activities against A549/lung adenocarcinoma cells (IC_50_ = 23.05 ± 1.45 and 11.63 ± 2.57 µM, respectively). The signaling Bcl-2 inhibition pathway was confirmed by molecular docking where significant docking energies and interactions with key Bcl-2 pocket residues were depicted. Moreover, the top active compound, **8b**, showed significant upregulated expression levels of pro-apoptotic/anti-apoptotic of genes; *Bax*, *Bcl-2*, *caspase-3*, *-8*, and *-9* through RT-PCR assay. Improving the compound’s pharmaceutical profile was undertaken by introducing **8b** within drug-solid/lipid nanoparticle formulation prepared by hot melting homogenization technique and evaluated for encapsulation efficiency, particle size, and zeta potential. Significant improvement was seen at the compound’s cytotoxic activity. In conclusion, **8b** is introduced as a promising anti-cancer lead candidate that worth future fine-tuned lead optimization and development studies while exploring its potentiality through in-vivo preclinical investigation.

## 1. Introduction

The failure of most cancer cells to undergo apoptosis confers them a survival advantage over normal cells. Apoptosis is a genetically programmed cell death to get rid of an undesirable cell, which is necessary for cell turnover [1]. Apoptosis is controlled by two major modes: the extrinsic and the intrinsic pathways. Both pathways lead to activation of caspases that result in morphological changes such as chromatin condensation, nuclear fragmentation, formation of apoptotic bodies and finally cell death [2].

The extrinsic pathway comprises external signals, that bind to the cell’s surface death receptors resulting in the formation of the death-induced signaling complex [3]. Bcl-2 (B cell lymphoma gene 2) family proteins control the intrinsic pathway. These proteins prevent the release of cytochrome C from mitochondria [4]. The Bcl-2 family of proteins can be classified as proapoptotic and antiapoptotic proteins. The conserved Bcl-2 homology domain-3 (BH3) of pro-apoptotic Bcl-2 members is responsible for the mitochondrial apoptosis [5]. Anti-apoptotic Bcl-2 proteins have a hydrophobic groove, containing both of the conserved Bcl-2 homology domains BH1 and BH2 which sequesters the BH3 of pro-apoptotic Bcl-2 members [6]. 

**Figure 1:**
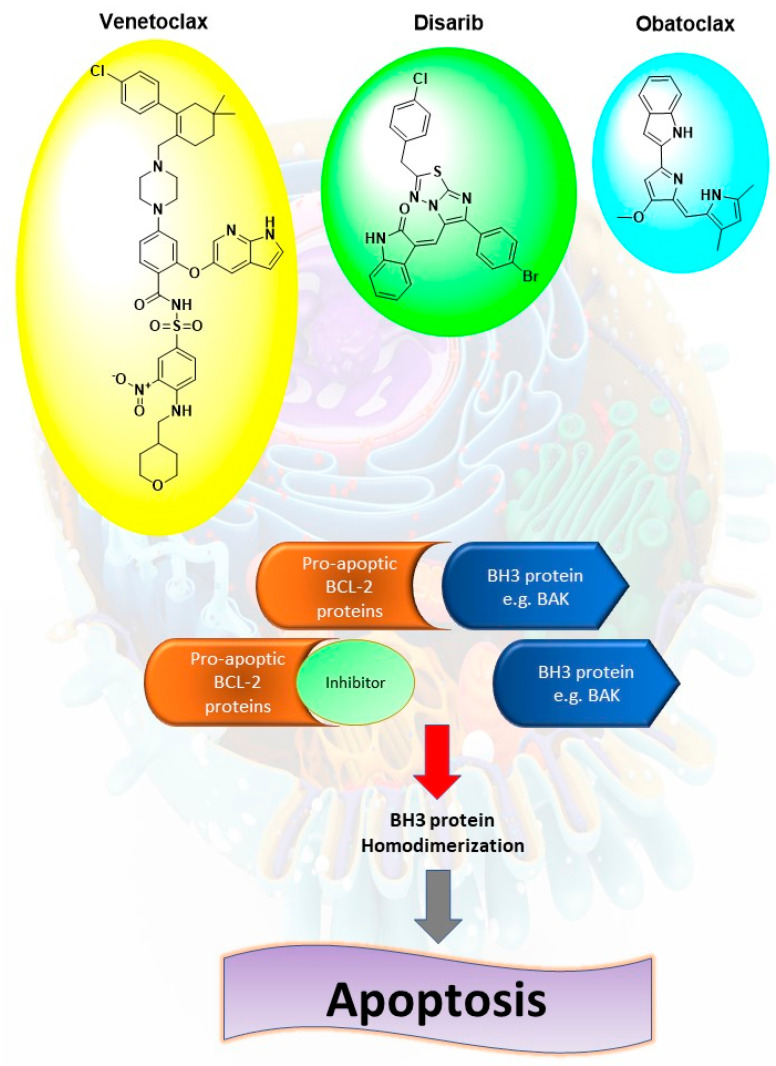
Mechanism of action of pro-apoptic Bcl-2 proteins small molecules inhibitors such as venetoclax, disarib and obatoclax, where they can disrupt the interactions of Bcl-2 and BH3 proteins such as BAK, leading to homodimerization of the BH3 proteins and activation of apoptotic cascade.

There are three approaches for targeting Bcl-2 in cancer therapy. The first one depends on the use of antisense oligonucleotide, which can bind to the complementary mRNA of Bcl-2, resulting in blocking expression at the protein level. The second strategy develops peptides against Bcl-2 protein [7,8]. Lastly, the third approach utilizes small molecules that can bind to the hydrophobic groove of anti-apoptotic Bcl-2 [9]. These molecules target one or more of P1–P4 sub-pockets in the BH3 groove of anti-apoptotic proteins. This binding results in releasing pro-apoptotic BH3-only proteins that can activate Bax and Bak and lead to apoptosis [10]. Several small molecules have been identified and synthesized, such as venetoclax, disarib, and obatoclax (Figure 1).

Venetoclax is a highly potent BH3 mimetic molecule. It showed a subnanomolar affinity to Bcl-2 (Ki < 0.010 nM) with no measurable affinities for other apoptotic proteins. It showed cytotoxicity against non-Hodgkin’s lymphoma, chronic lymphocytic leukemia (CLL) and acute leukemias [11]. Disarib is another selective Bcl-2 inhibitor [12]. Disarib was proven to inhibit the growth of Bcl-2 high cancer cell lines and CLL patient primary cell lines with minimum effect on Bcl-2 low cancer cell lines. In addition, disarib exhibited fewer side effects in treated animals with respect to platelet count, body weight, and liver and kidney functions [13]. Obatoclax is an oligopyrrole which antagonizes Bcl-2 and Bcl-xL [14]. Obatoclax binds to the BH3 domain of Bcl-2 and interrupts the interaction with proapoptotic proteins. The combination of obatoclax and lapatinib, gifitinib, bortezomib, and entinostat showed synergistic repression of cell growth in human breast cancer cells [15]. Furthermore, obatoclax synergizes chemotherapeutic agents as cisplatin, in cancer cell lines [16].

For Bcl-2 inhibitors development, we have described several series of compounds with chemical scaffolds that mimic obatoclax by either replacement of the pyrrole ring of obatoclax with benzimidazole or extending the structure after the indole ring. Additionally, comprehensive biological and molecular docking investigation, within the crystallised Bcl-2 crystal structure, provided further insights regarding the molecular pro-apoptotic mechanisms of the presented small synthesized molecules. Finally, formulating the most promising lead within an optimized solid lipid nanoparticle formulation provided beneficial results concerning improved cytotoxic activity, drug release and pharmaceutical properties, particularly drug’s aqueous solubility and bioavailability. 

## 2. Results and Discussion

### 2.1. Compounds Design

The reported drug-like pan Bcl-2 inhibitor, obatoclax (GX15_070), was adopted as the lead framework for structural modifications and manipulations. Obatoclax is BH3-mimetic small molecule (317.4 Da) acting on several anti-apoptotic proteins such as Mcl-1, Bcl-2, Bcl-W, and Bcl-XL [17]. Preclinical studies illustrated the drug capability in reversing BH3-mediated binding of Bcl-2 family proteins to apoptotic proteins, Bak and Bax, allowing unopposed Bak/Bax dimerization and subsequent initiation of intrinsic apoptotic signaling [18,19]. Several additional mechanisms of action have been accounted for obatoclax anticancer activity, including induction of autophagic cell death and necroptosis [19,20,21,22]. Moreover, obatoclax can inhibit cell proliferation through seizing cell cycle development at G1/S-phases [19,23]. Many reported studies confirmed the clinical advent of obatoclax for inducing apoptosis within cells derived from solid tumors and hematological cancers such as small cell lung cancer, acute myeloid leukemia, and Hodgkin’s lymphoma as well as potentiating cytotoxicity of targeted therapy drugs and conventional chemotherapeutics.

**Figure 2:**
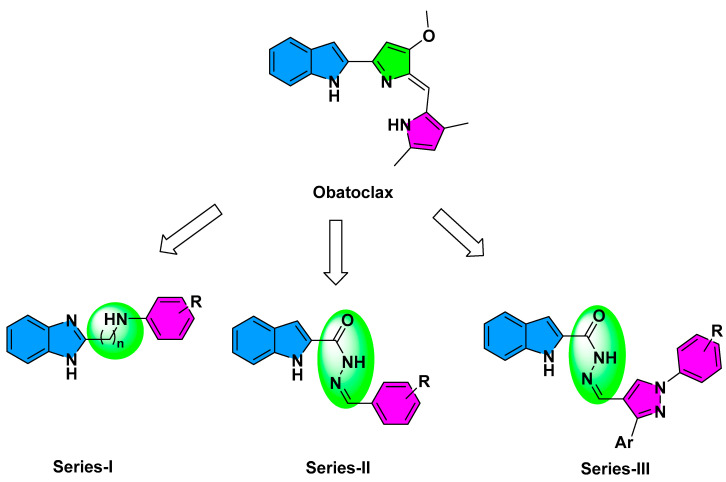
Rationalized design of the proposed new pro-apoptotic agents (Series-I-to-III), relying on the pan Bcl-2 inhibitor, obatoclax.

Currently, there is no reported atomic resolution data concerning the obatoclax being bounded to pre-survival Bcl-2 protein. Nevertheless, Zacarias-Lara et al. have reported seven recognized Bcl-2 inhibitors, including obatoclax, to be investigated for their respective affinities and binding interactions through molecular docking calculations [24]. Interestingly, the authors suggested anchoring of obatoclax just adjacent the hydrophobic cleft (H1-3) which may compromise the drug’s ability to dissociate BH3-only pro-apoptotic proteins for subsequent programmed cell death. Driven by the above findings, the presented study aimed for developing new pro-apoptotic agents, based on obatoclax principal pharmacophore, with an extra goal of improved Bcl-2 hydrophobic cleft accommodation. Obatoclax belongs to the class of organic compounds, designated as dipyrrins, incorporating two pyrrole rings within their chemical architectures where the two 5-membered rings are being fused via a trivalent methine (-CH=) bridge. Here within the presented manuscript, three structural modification strategies have been adopted to design the newly synthesized compounds, Series-I-to-III (Figure 2). The first modification strategy was to improve the obatoclax flexibility through replacing the drug’s central pyrrolylmethine linker with straight alkylamine or carboxyhydrazine methylene linkers furnishing the members of the Series-I or -II, respectively. The aim of such tactic was to permit enhanced maneuver for these newly synthesized compounds to better accommodate the Bcl-2 major hydrophobic pockets (S1-to-5). Additionally, the terminal obatoclax pyrrole ring was replaced with substituted phenyl groups to permit an enriched π–π stacking and other non-polar interactions with the hydrophobic residues at the Bcl-2 pocket.

Within a comparable bases, members of Series-I possess the benzimidazole scaffold as the classical isostere of the indole moiety present within the obatoclax chemical architecture. Such introduced second structural modification relied on the ability of the benzimidazole ring to permit extra H-bonding with the surface polar residues of the Bcl-2 pocket and thus further ligand fixation. Notably, both members of Series-I and -II were of comparable overall size as that of the obatoclax, the thing that can limit the extension of the synthesized compounds to the farthest hydrophobic pockets. Therefore, a third and last structural modification strategy has been adopted where the terminal substituted phenyl moieties at Series-II were replaced with the highly extended substituted bisaryl pyrazole scaffold resulting in the members of Series-III. The latter members possess the double advent of great flexibility and extended size. Finally, the adoption of the central pyrazole scaffold was rationalized for its straightforward synthesis as well as easily derivatized during the pyrazole ring closure. Based on the adopted three structural modification strategies, fourteen compounds were synthesized as well as evaluated for respective predicted anti-cancer activities through in-vitro and in-vivo biological analyses as well as molecular docking investigations.

### 2.2. Chemistry

**Scheme 1:**
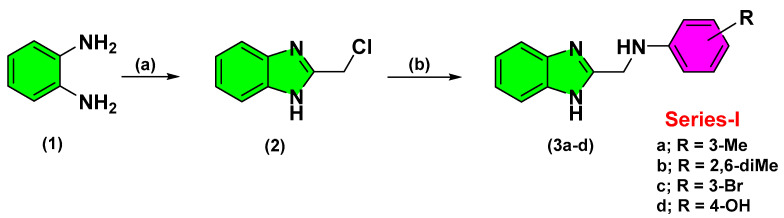
Reagents and conditions for Series-I: (**a**) ClCH_2_COOH, 4N HCL, reflux, overnight, 40%; (**b**) Ar-NH_2_, 51–73%.

The synthesized compounds of the three designed series were obtained from straightforward schematic pathways. Structural diversity for synthesizing the target compounds was performed at the final steps of the synthetic pathways. Adopting such strategy was beneficial for minimizing the number of needed reactions that would provide each individual analogue. Within Scheme 1, Series-I benzimidazole derivatives were synthesized from the commercially available, ortho-phenylene diamine (**1**) through Phillips-Ladenburg reaction [25]. Cyclization was proceeded through refluxing ortho-phenylene diamine with chloroacetic acid, under acidic conditions, to yield the corresponding intermediate 2-(chloromethyl)-1H-benzimidazole (**2**). Introduction of 2-chloromethyl arm, within the benzimidazole skeleton, served as a handy scaffold for introducing structural diversity at the final synthetic pathway. Subsequent S_N_2 nucleophilic substitution at 2-methyl benzimidazole with various aromatic amines furnished the target substituted 2-aminomethyl-benzimidazole derivatives (**3a**–**d**) depicting extra aromatic signals at their ^1^H-NMR spectra [26].

**Scheme 2:**
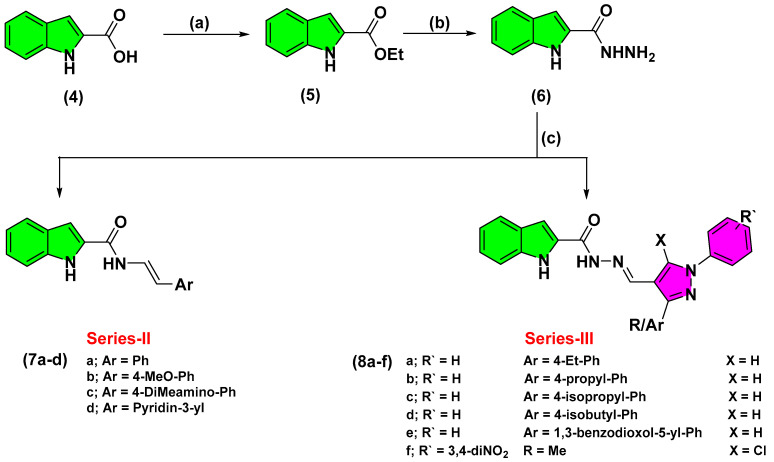
Reagents and conditions for Series-II and -III: (**a**) EtOH, Conc. H_2_SO_4_, reflux, 1.5 h, 92%; (**b**) Hydrazine hydrate, absolute EtOH, reflux, 6 h, 44%; (**c**) Ar-CHO, EtOH, glacial acetic acid, reflux, 1.5–3 h, 30–45%.

**Scheme 3:**
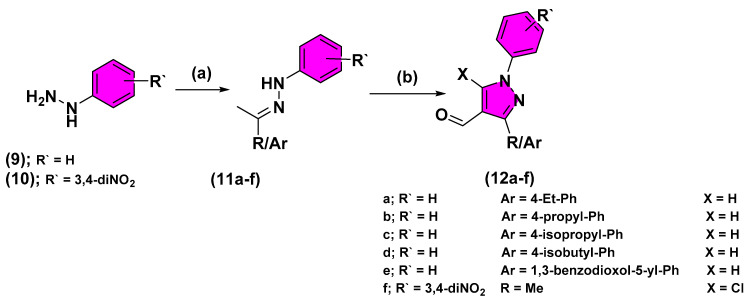
Reagents and conditions for the synthesis of pyrazole aldehydes: (**a**) EtOH, Ar/R-COCH_3_, 80 °C, 1 h, 80–97%; (**b**) POCl_3_, DMF, reflux, 4 h, 61–81%.

The indole-based derivatives (Series-II and -III) were synthesized starting from the commercially available indole-2-carboxylic acid (**4**) (Scheme 2). Throughout Fischer reaction, refluxing **4** with concentrated H_2_SO_4_ and ethanol yielded the corresponding ethyl,1H-indole-2-carboxylate (**5**) [27]. Subsequent, condensation of the obtained ester intermediate with hydrazine hydrate furnished the hydrazide key intermediate (**6**). Finally, Schiff’s base condensation of **6** with different commercially available and synthesized aromatic aldehydes, under acidic conditions, provided the target hydrazone analogues (**7a**–**d** and **8a**–**f**) [28]. Appearance of the characteristic methylene hydrogen at NMR spectra (δ_H_ = 8.16–8.98 ppm; δ_C_ = 146.8–148.9 ppm) confirmed successful Schiff’s base formation. Preparation of several pyrazole-based aldehydes was proceeded through three-step chemical synthesis [29]. Synthesis of the pyrazole aldehydes, that served as the structural building blocks of the Series-III compounds, was proceeded through initial acidic condensation of different ketones with phenylhydrazine derivatives yielded the corresponding imine analogues (**10a**–**f**) (Scheme 3). Subsequently, Vilsmeier-Haack reaction, with the advent of phosphorous oxychloride, provided the target pyrazole-based aldehydes (**12a**–**f**) exhibiting characteristic NMR signals of CHO groups (δ_H_ = 9.97–10.00 ppm; δ_C_ = 185.1–185.3 ppm).

### 2.3. Biological Activity

#### 2.3.1. In-Vitro Cytotoxic Activity

The potential anti-cancer activity of all fourteen final synthesized compounds was investigated through MTT cytotoxic assay against two different human solid cancerous cell lines; A549/lung and MDA-MB-231/breast adenocarcinoma. The adopted cell-proliferation assay assessed % cell viability in relation to the reducing activity of cellular NADPH-dependent cellular oxidoreductases on tetrazolium dye, MTT [2,5-diphenyl-3-(4,5-dimethylthiazol-2-yl)tetrazolium bromide salt], for furnishing an easily quantified insoluble chromogenic formazan [30]. For each drug concentration, 0.1, 1, 10, and 100 μM, the % cell-viability was estimated relative to negative control following a complete 48 h of drug treatment. Both 5-fluorouracil (5-FU) and doxorubicin (DOX) were set as positive index comparators. Moreover, the safety profile of the synthesized compounds was further investigated by examining their in-vitro cytotoxicity on non-cancerous cell line; MDCK/kidney cells.

**Figure 3:**
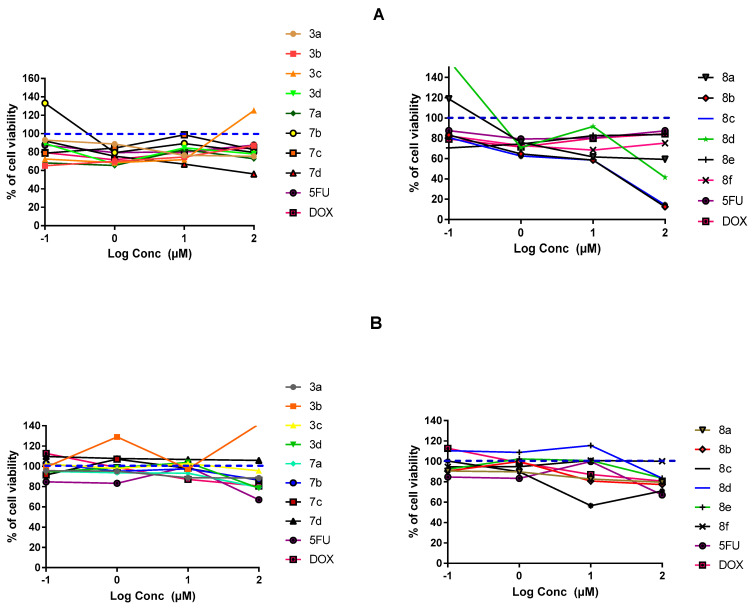

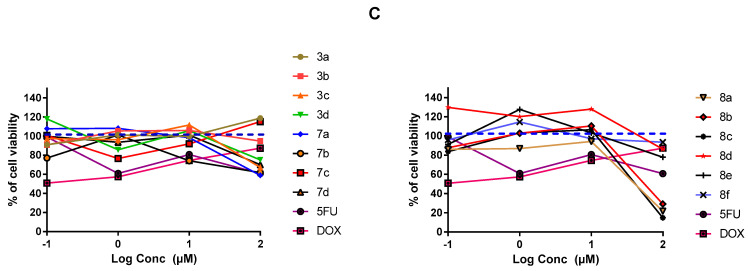
In-vitro cytotoxicity of the fourteen final synthesized compounds on cancerous and non-cancerous cell lines. (**A**) MDA-MB-231/breast cancer cells; (**B**) A549/lung adenocarcinoma cells; (**C**) MDCK/kidney non-cancerous cells. At 1 × 10^4^ cells/well cell density, cells were treated with several drug concentrations (0.1, 1, 10, and 100 µM) of investigated compounds or positive comparator; 5-FU and DOX, for 48 h exposure time. Cytotoxicity was identified via MTT-based colorimetric assay (490 nm) and % cell growth viability was estimated in triplicates as compared to vehicle negative controls representing the untreated cells. Blue dotted line indicates this control.

Findings within Figure 3A illustrate a dose-dependent impact of several synthesized compounds on MDA-MB-231/breast cell proliferation. Rising drug concentration was directly related with elevated cell growth inhibition up to the highest applied concentration (100 µM) (*p* < 0.05). Interestingly, most of the tested compounds had a dose dependent cytotoxicity or a plateau effect, except for compound **3c** on MDA-MB-231/breast cancer where it enhanced cell proliferation at 100 µM drug concentration. The steepest decline within the cell proliferation was seen with **7c**,**d** and **8a**–**d** reaching down to very low % cell viabilities (12% and 14%) at 100 μM concentrations. The latter suggests great susceptibility of MDA-MB-231/breast cancer cells towards indole derivatives, particularly those having pyrazole-based scaffolds (Series-III). The rest of compounds exhibited comparable cytotoxicity patterns as those for positive reference controls, 5-FU and DOX, since minor %viability fluctuations were depicted across the applied drug concentrations. Regarding the drug’s impact on A549/lung adenocarcinoma cell proliferation, both investigated and positive control compounds showed quite lower overall anticancer activity as compared to their actions up on the MDA-MB-231/breast cancer cells. Nevertheless, comparable differential activity profiles were depicted among the investigated compounds, where compounds **8a**–**c** exhibited the highest activity profiles with steepest decrease in % cell viability (70–79%; *p* < 0.001) at 100 µM concentrations (Figure 3B). This again suggests promising anticancer activity of the indole-based compounds on A549/lung cancer cells, particularly for the ones incorporating the pyrazole scaffolds. The rest of the compounds exhibited comparable pattern of cell growth inhibition across their concentration range which was also quite similar to that of positive control drugs. However, it was only for compound **3b** where its anticancer activity on A549/lung cancer cells depicted irregular effects with tendency to increase the cell proliferation. Evaluating drug’s cytotoxicity on non-cancerous kidney cells, Figure 3C showed higher safety profiles for synthesized compounds compared to controls, 5-FU and DOX. Significantly higher vitality patterns were illustrated for all compounds, particularity over the concentration range 0.1–10 µM, as compared to those of reference compounds (*p* < 0.001). 

For gaining more insights about the efficacy/safety profiles of the synthesized compounds, IC_50_ values for promising cytotoxic compounds were estimated over the three cell lines. Only compounds exhibiting significant cytotoxicity, with cut-off value < 85% vitality at 100 µM on both cancerous cell lines, were considered relevant for IC_50_ estimating and warrant further consideration. Interestingly, the selected compounds for MDA-MB-231/breast cancer cells (**3a**, **7b**, **7d**, and **8a**–**d**) illustrated relevant IC_50_ values reaching down to two-digit micromolar activity (Table 1). Members of Series-III showed the lowest IC_50_ (down to 12.69 µM) suggesting the preferential cytotoxicity of their substituted pyrazole arms. Both **8d** and **8f** showed higher IC_50_ (21.64 and 31.46 µM, respectively) suggesting activity preferentiality for aromatic substitution, at C3 pyrazole ring, while tolerating small-sized aliphatic chains. Compound **7d** was comparable to the top-active members of Series-III with IC_50_ 17.38 µM, suggesting Series-II preferentiality for pyridine ring substitution over methoxy phenyl synthon. Regarding IC_50_ on MDA-MB-231/lung adenocarcinoma, only **8b** and **8c** were evaluated suggesting preferential activity for pyrazole substitution with branched aliphatic chains. Finally, the IC_50_ for selected Series-III compounds on non-cancerous/kidney cells were much higher than the other two Series, reaching up to IC_50_ 92 µM activity for **8b**. Based on all the above findings, significant cytotoxic activity has been assigned for members of Series-III which were also presented with high safety profile, particularly **8b**, on non-cancerous cell line. Therefore, compound **8b** was considered promising with double the benefits of high efficacy/safety profiles making it worthy of further investigations.

#### 2.3.2. Morphological Assessment

**Figure 4:**
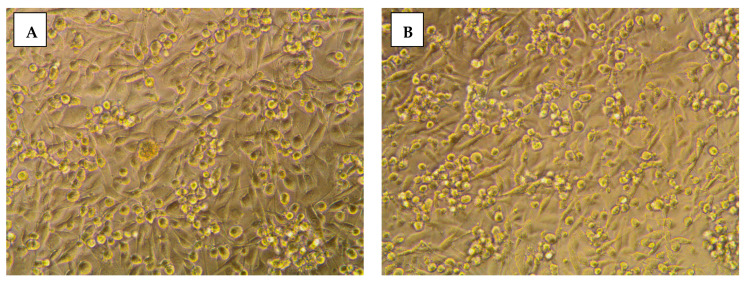

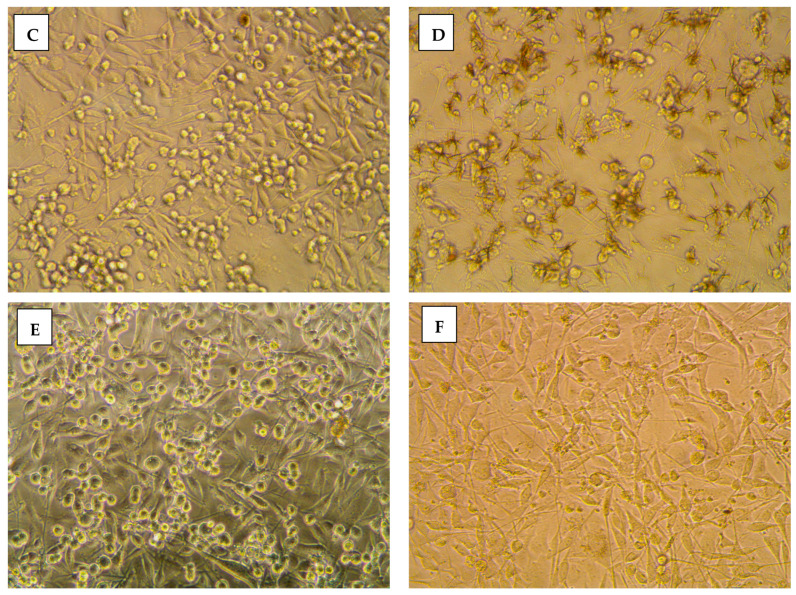
Morphological alterations of MDA-MB-231/breast cancer cells following 48 h incubation and photographed with phase-contrast microscope. (**A**) vehicle control; (**B**) 0.1 µM 8b; (**C**) 1 µM 8b; (**D**) 10 µM 8b; (**E**) 100 µM 8b; (**F**) 100 µM 5-FU; Magnification power ×20.

**Figure 5:**
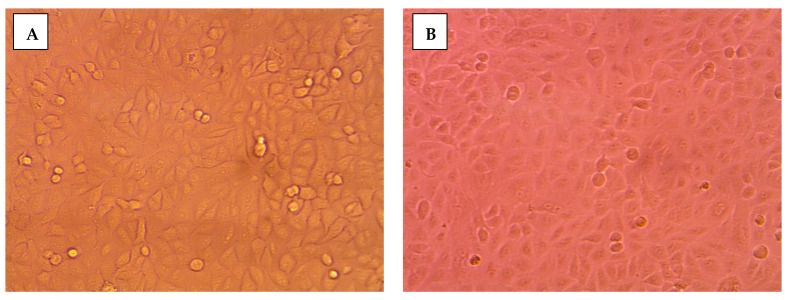

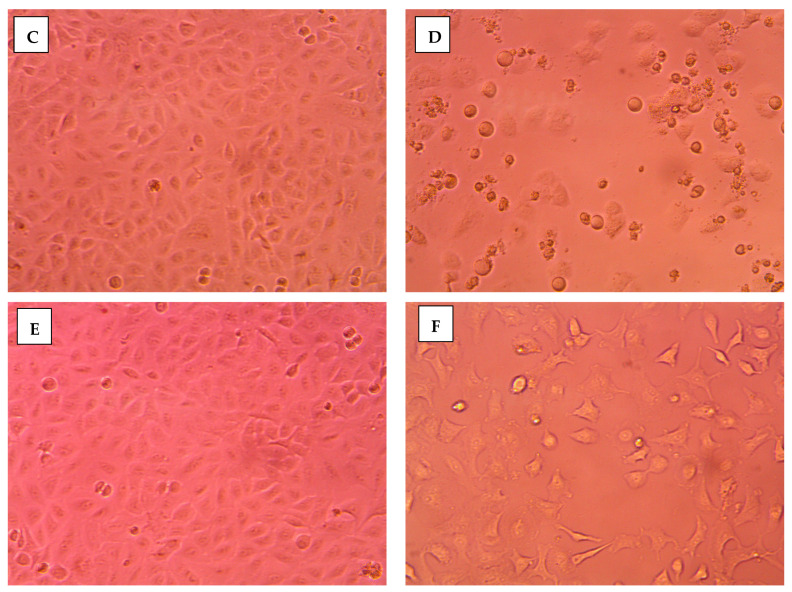
Morphological alterations of A549/lung carcinoma following 48 h incubation and photographed with phase-contrast microscope. (**A**) vehicle control; (**B**) 0.1 µM 8b; (**C**) 1 µM 8b; (**D**) 10 µM 8b; (**E**) 100 µM 8b; (**F**) 100 µM 5-FU; Magnification power ×20.

The impact of **8b** on cancerous cellular morphology was evaluated over different concentrations following 48 h exposure time. In MDA-MB-231/breast cancer cells, normal membrane integrity and control group nucleus morphology was depicted for cells within vehicle negative control group (Figure 4A). Contrarily, cells treated with **8b** exhibited significant cell morphology alteration, intercellular space dilatation, and cellular shrinkage (Figure 4B). Alterations proceeded within a dose-dependent manner as escalated drug concentrations showed cells being gradually shrunken, unable to adhere well, floated, and clustered together (Figure 4C,D). At 100 µM concentration, the cell number became highly declined and great loss of cellular polyhedral shape was observed as cells acquired the tentacled spindle-shaped morphology (Figure 4E). These morphological changes were more drastic with **8b** as compared to 5-FU for similar concentrations (100 µM) (Figure 4F). Comparable cell morphology changes were observed with A549/lung adenocarcinoma (Figure 5A–E). Nevertheless, an epithelial-mesenchymal transition was observed within 5-FU group, rather than **8b**, at same drug concentration (100 µM) (Figure 5F). The later finding presents a significant advantage for **8b** as it did not trigger this evolutionarily-conserved developmental program conferring metastatic properties upon cancerous cells through enhanced mobility, invasion, and apoptotic stimuli resistance [31].

#### 2.3.3. Apoptosis Rate and Cell Cycle Analysis

**Figure 6:**
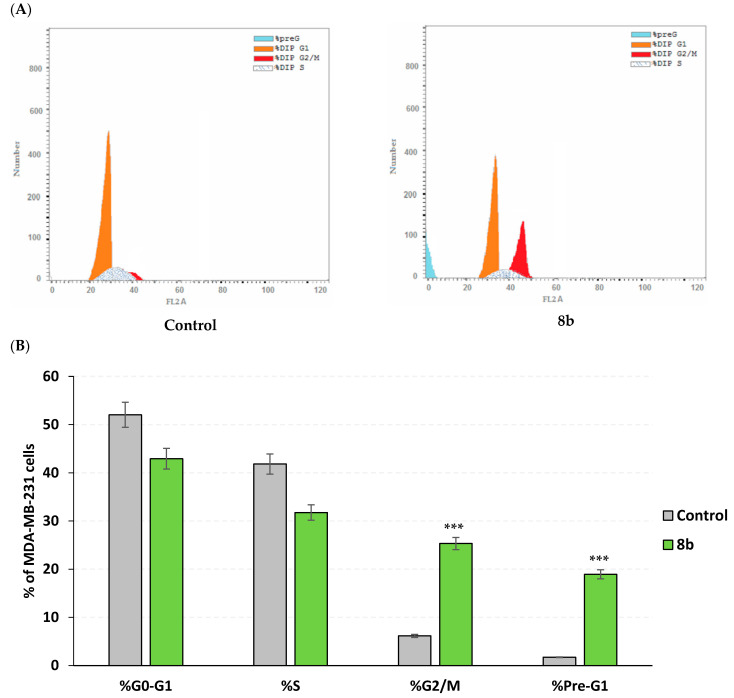
Impact of compound **8b** upon cell cycle distribution within MDA-MB-231/breast cancer cell line. Following 48 h-period incubation of cells with either **8b** (13 µM; approximated IC_50_) or vehicle negative control [0.1% DMSO (*v*/*v*)], cells were fixed, stained with propidium iodide (PI), and assessed via flow cytometry; (**A**) DNA histograms for cell cycle distribution of untreated cells (left panel) and **8b** group (right panel) at in 2N (G0G1), >2N (S), 4N (G2/M), and <2N (pre-G1), where cell number was correlated against gated counts/DNA area (FL2-A) using CellQuest^®^ software; (**B**) Bar-chart representation of cell accumulation percentage within each stage of cell development. All data are represented as mean of three independent experiments ± SEM. Statistical analysis was performed using Student-*t*-test (***, *p* < 0.001).

**Figure 7:**
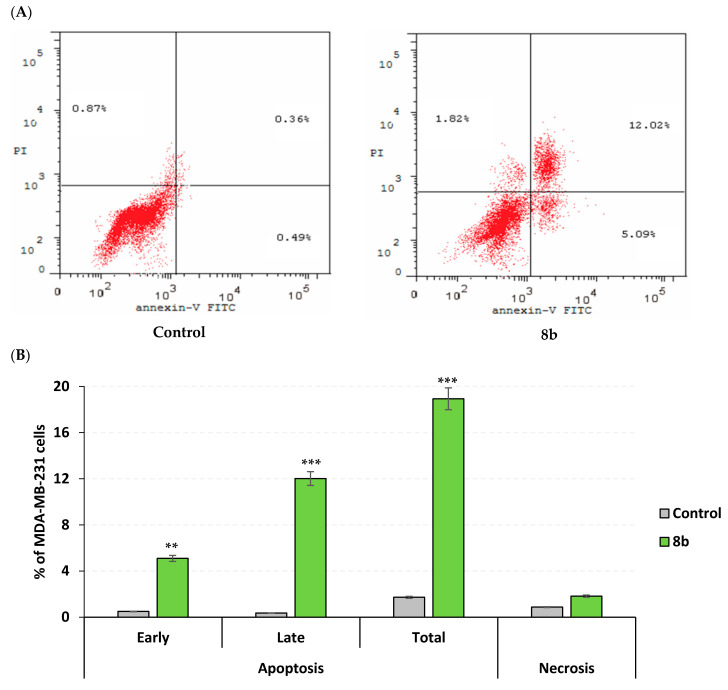
Impact of compound **8b** on apoptosis rate within MDA-MB-231/breast cancer cell line (Annexin-V/FACS). (**A**) Apoptotic impact of **8b** following cell double staining via Annexin-V-FITC then PI, and then 48 h incubation time with either **8b** (13 µM; approximated IC_50_) or vehicle negative control [0.1% DMSO (*v*/*v*)]; (**B**) Bar-chart representation of quantitative analysis of apoptosis, across different stages, as well as necrosis. The four quadrants are recognised as: LL, LR, UR, and UL for viable, early apoptotic, late apoptotic, and necrotic cell populations, respectively. All data are represented as mean of three independent experiments ± SEM. Statistical analysis was conducted via Student-*t*-test (**, *p* < 0.01; ***, *p* < 0.001).

Further investigation of **8b** mechanistic growth inhibitory action on MDA-MB-231/breast cancer, both cell cycle, and apoptosis rate analysis were conducted at the compound’s approximated IC_50_ value (13 µM). Using flow cytometry, apoptosis rate analysis was proceeded through staining the cell surface-translocating phosphatidylserine with Annexin-V fluorescent conjugate, the calcium-dependent phospholipid binding protein [32]. However, cell cycle analysis was achieved using propidium iodine fluorescent dye to stoichiometrically stain cell DNA contents allowing quantitation and identification of all cell phase rates; G0-G1, S, G2/M, and pre-G1 [33,34]. The impact of **8b** on cell cycle distribution depicted predominant cell population at G2/M stage (25.32%) was significantly higher than that of untreated cell line (6.15%; *p* < 0.001) (Figure 6A). The elevated cell population at G2/M stage was complemented by significant reduction at pre-G1 stage of treated cells as compared to negative controls (1.72% vs. 18.93%; Figure 6B). For identifying the mode of cell death promoted by **8b** within MDA-MB-231/breast cancer cells, apoptosis rate analysis was performed following 48 h exposure time. Compound **8b** (13 µM) induced both early and late stages of apoptosis in breast cancer cell line with significantly elevated % apoptotic cell levels as compared to controls (*p* < 0.01 and *p* < 0.001, respectively) (Figure 7). Moreover, the average proportion of Annexin-V stained positive cells (total apoptotic cells) elevated from 1.72% within untreated cells to 18.93% in treated ones (*p* < 0.001). Interestingly, compound **8b** showed no influence on the necrosis of MDA-MB-231/breast cancer cells. The above provided findings are supported by the previous cell cycle analysis confirming the potentiality of **8b** as promising anticancer agent.

#### 2.3.4. DNA Fragmentation Determination

**Figure 8:**
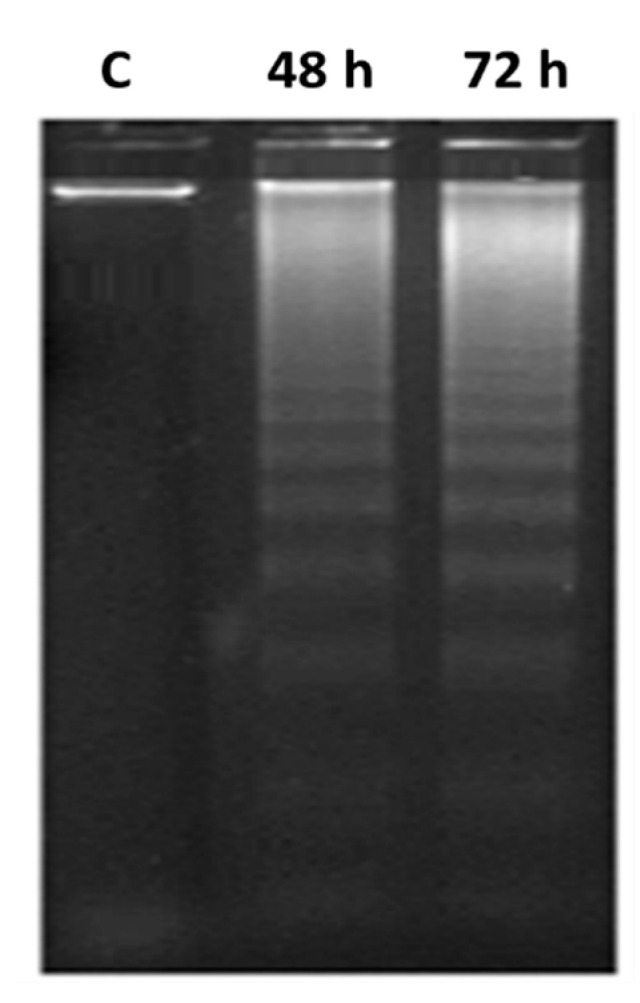
DNA fragmentation of MDA-MB-231/breast cancer cell line treated with **8b**. Fragmentations of genomic DNA in breast cancer cells were treated with **8b** (13 μM) for 48 or 72 h exposure time. Low-molecular-weight DNA from homogenized cells was resolved and visualized on ethidium bromide-stained 1.5% agarose gel while being photographed by UV-illumination electrophoresis; C = DMSO vehicle control.

In order to delineate the mechanistic aspects of MDA-MB-231/breast cancer cell death mediated by **8b**, the DNA fragmentation assay was conducted as being highly characteristic for apoptosis. Significant DNA fragmentation was depicted with **8b** at IC_50_ doses (13 µM) at various time-intervals; 48 h and 72 h following cell treatments. A typical ladder pattern of internucleosomal fragmentation was observed from cell homogenates within both incubation periods (Figure 8). Such findings further confirm the significant activity of 8b as a potent inducer of apoptosis.

#### 2.3.5. Apoptotic Gene Expression and Protein Level Analysis

Compound **8b** demonstrated strong cytotoxic impact upon breast cancer cell lines, as it accomplished significantly low IC_50_ value (12.71 μM) with confirmed apoptosis induction. Dissection of the compound’s proapoptotic activity was proceeded through gene expression analysis for key genes, controlling apoptosis pathway, as well as protein level determination. Following **8b** treatment for 48 h, alterations within MDA-MB-231/breast cancer cell expression of *Bcl-2, Bax*, *caspase-3*, *-8*, and *-9* genes, as well as cytochrome c and class-III β-tubulin proteins levels were determined using real-time polymerase chain reaction (RT-PCR) or ELISA technique, respectively. Typically, Bcl-2, Bax, caspases, and cytochrome c proteins contributes within the regulation of apoptotic signaling. Acting as apoptotic activator (Bax) or inhibitor (Bcl-2), the Bcl-2 family proteins play their significant role in apoptosis [35]. The caspases family are cysteine proteases being classified as either executioners; caspase-3, -6, or -7 or initiators; caspase-8 and -9 [36]. Regrading caspase-8, its activation is proceeded through extrinsic death-receptor dependent apoptotic pathway, while, caspase-9 activation is within the event of intrinsic mitochondrial cytochrome c leakage [37]. Additionally, participation of activated caspase-3 is to be essential for caspase-8 activation [38]. Class-III β-tubulin are pure prognostic biomarker within cancer patients being associated with aggressive phenotypic/drug-resistant cancers and part of complicated pro-survival molecular pathway, being triggered via poor nutrient supply and hypoxia [39].

**Figure 9:**
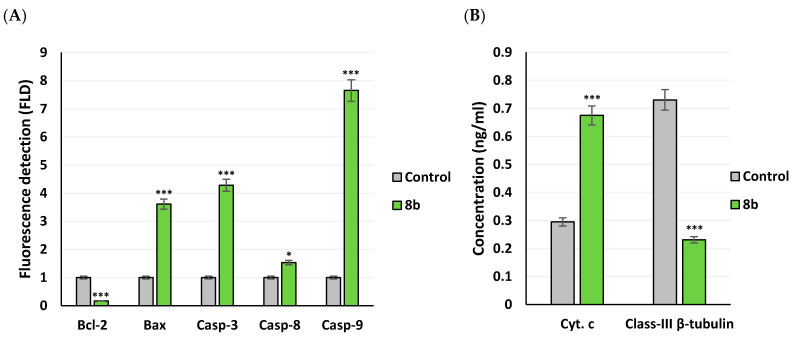
Apoptotic gene expressions and protein level analysis of MDA-MB-231/breast cancer cells exposed to **8b**. (**A**) Expression of pro-apoptotic/anti-apoptotic of genes; *Bax*, *Bcl-2*, *caspase-3*, *-8*, and *-9* using RT-PCR method; (**B**) Evaluation of cytochrome c and class-III β-tubulin protein levels in MDA-MB-231/breast cancer cells using ELISA technique. Within both assays, cultivated MDA-MB-231/breast cancer cells were treated with **8b** at its IC_50_ concentration (13 μM) for 48 h exposure period. All data are represented as average of three independent experiments ± SEM. Statistical analysis was performed using Student-*t*-test (*, *p* < 0.05; ***, *p* < 0.001).

Findings within Figure 9A illustrated strong stimulated expression of pro-apoptotic *BAX* gene (3.61 folds), and apoptotic genes; *Caspase-3* (4.28 folds), *Caspase-8* (1.53 folds), and *Caspase-9* (7.65 folds) as compared to negative controls (*p* < 0.05 and *p* < 0.001). Nevertheless, significant downregulation of anti-apoptotic gene *Bcl-2* (0.165 folds) was depicted and have been translated into elevated *Bax/Bcl* expression ratio (1 → 21.88; *p* < 0.001). The *Bax/Bcl-2* gene expression ratio can serve as early predictor for cancer in patients as well as sensitive monitor of cancer progression [40]. On the other hand, compound **8b** illustrated increased levels of the apoptotic stimulator cytochrome c (0.675 ng/mL vs. 0.295 ng/mL; *p* < 0.01) and down-regulated levels of class-III β-tubulin (0.23 vs. 0.73 ng/mL; 68.36% inhibition; *p* < 0.001) as compared to controls (Figure 9B). Gathering up all the provided evidence, up-regulation of *Caspase-3*, *-8*, *-9* and *Bax*, while down-regulation of *Bcl-2* genes and class-III β-tubulins are suggested believed to be related to compound **8b**-induced apoptosis within breast cancer cell line.

### 2.4. Computational Study

**Figure 10:**
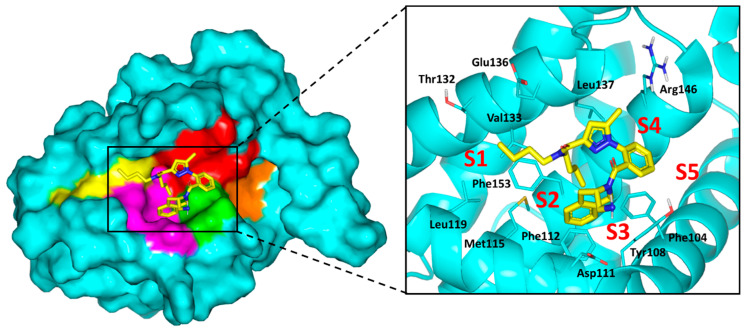
Structure of X-ray crystallized Bcl-2 (PDB ID: 6qgk) bounded to tetrahydroisoquinoline-phenyl pyrazole derivative along the binding site. Five hydrophobic pockets (P1–5), constituting the binding site, are shown in 3D-surface presentation and differently colored; P1 yellow, P2 magenta, P3 green, P4 red, P5 orange. Zoomed image is stereoview of crystallized ligand (yellow sticks) at binding site. Sub-pockets are labeled with red letters and only significant residues (cyan lines) located within 4 Å radius of bound ligand are displayed and labeled with sequence number.

To gain more insights about differential cytotoxic activity of synthesized compounds, molecular docking simulations were conducted for all agents. The binary complex of X-ray crystallized Bcl-2 (PDB ID: 6qgk; resolution 1.80 Å) [41], bounded to tetrahydroisoquinoline-phenyl pyrazole derivative, was adopted for the docking studies. Adopting such complex for the presented in-silico investigation was owing to structural similarities of crystallized ligand with the synthesized compounds as well as its extended orientation within the target protein. Typically, the Bcl-2 pocket is a narrow, long grooved site comprised of two larger nearby sub-pockets (P2 and P4), in addition to, three small hydrophobic pockets (P1, P3 and P5) [9]. The P1 through P4 sub-pockets are the key Bcl-2 binding sites for the class of single BH3-only pro-apoptotic proteins. Owing to the hydrophobic nature of the binding site residues, it has been challenging for inhibitors to competitively overcome the natural substrate-Bcl-2 interactions. However, significant polar residues (Tyr108, Asp111, Glu136, Met115, and Arg146) represent relevant anchoring sites for stabilizing the bounded ligands. The crystallized ligand showed extended accommodation within the target pocket, reaching from P1-to-P4 subsites (Figure 10). Significant non-polar contacts are depicted with the hydrophobic residues of four sub-pockets, while the ligand’s polar functionalities exhibit close proximity towards Tyr108, Asp111, and Arg146. Interactions with these polar residues are suggested to be significant for the ligand/target stability [41].

**Figure 11:**
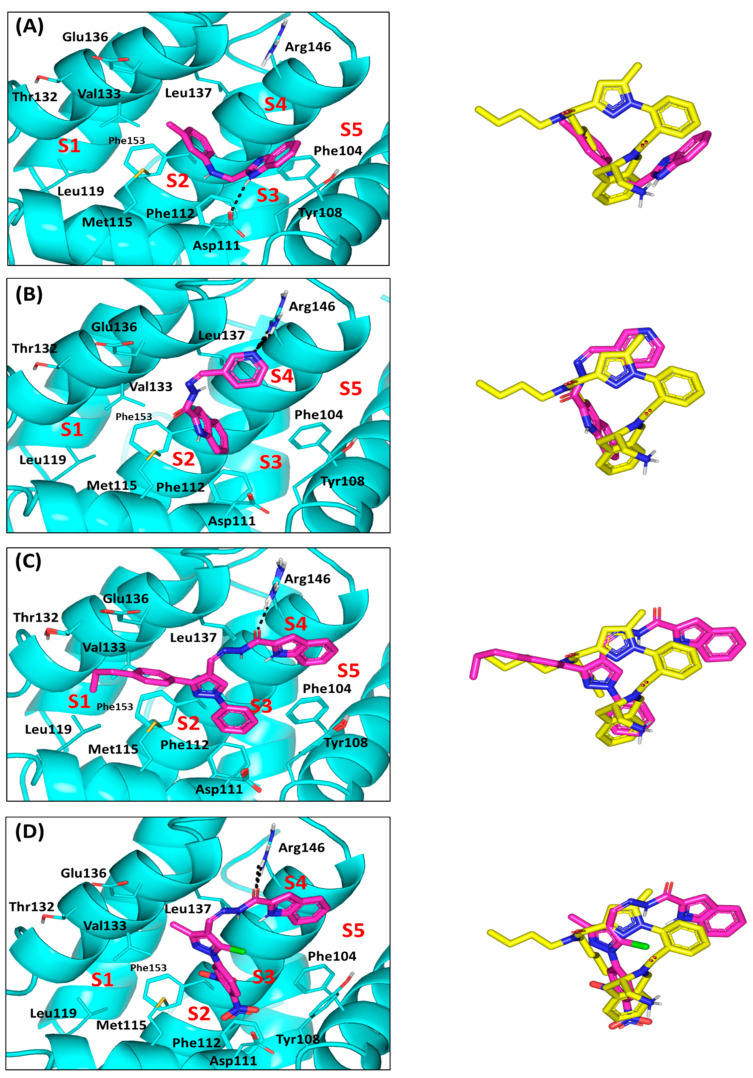
Docking results of synthesized compounds within Bcl-2 protein target (PDB ID: 6qgk). Left panels illustrate the proposed docking poses for the investigated compounds (magenta sticks) at the hydrophobic surface binding site of Bcl-2 target (cyan cartoon); (**A**) **3a**; (**B**) **7d**; (**C**) **8b**; (**D**) **8f**. On the right is the overlay of docked synthesized compounds (magenta sticks) and crystallized ligand (yellow sticks), depicting their comparative orientations within the binding site. Only significant residues (cyan lines) located within 4 Å radius of docked synthesized compounds are displayed and labeled with sequence number and colored. Hydrogen bonds are displayed as black-dashed lines.

Proceeding throughout the docking studies, validation of the adopted docking protocol was achieved through redocking the crystallized ligand (PDB ID: J1Q) showing a root-mean standard deviation (RMSD) below 2 Å. Depicting RMSD values below 2 Å indicates that both the adopted algorithms and parameters were sufficient for determining the best docking pose [42]. Regarding the docking results of the top cytotoxic drugs, limited binding of the Series-I compound (**3b**) towards Bcl-2 pockets was illustrated. The benzimidazole-based ligand adopted V-shaped conformation with limited binding to P2 and P3 hydrophobic pockets. The benzimidazole scaffold predicted relevant anchoring within P3 through H-bonding with Asp111, while the substituted phenyl group finely overlaid at P2 pocket (Figure 11A). This orientation was further stabilized through π-π stacking of the benzimidazole ring with Tyr108 (Supplementary Materials; Table S1). More extended orientations were depicted for the Series-II indole derivatives (**7a** and **7b**). Binding was extended at P2 through P4 sub-pockets showing their indole rings being docked at P2. Higher docking score was assigned for **7d** (*S* = −4.8585 Kcal/mol vs. −4.76959 Kcal/mol for **7b**), where double polar interactions between its pyridine ring and Arg146 side chain justified significant anchoring within pocket P4 (Figure 11B). Moreover, the pyridine ring was further stabilized through double π-hydrogen interaction with Leu137 of P4 subsite suggesting its great affinity to Bcl-2 protein. Owing to its amide linker flexibility, **7d** indole ring showed better overlay with the crystallized ligand than does the **3a** benzimidazole scaffold.

For the pyrazole-based Series-III ligands, comparable binding modes, to the crystallized ligand, were suggested (RMSD = 1.211 to 1.7374 Å) owing to a similar topology of central pyrazole with substituted aromatic arms. Extended orientations and conformations over P1-P4, with much closer proximity towards P5 subsite, have been predicted for Series-III ligands. Compound **8b** showed one of the highest docking scores (*S* = −6.1846 Kcal/mol), which was explained by its exhibiting significant hydrogen bonding between its amide linker and Arg146 side chain at P4 subsite (Figure 10C). The same polar interaction was predicted for the other Series-III members confirming their superior Bcl-2 affinity (*S* = −5.7070 to −6.2460 Kcal/mol) and the importance of Arg146 in ligand binding. Notably, compound **8f** was suggested with the lowest member docking score (***S*** = −4.9429 Kcal/mol) for lacking contacts with P1 due to its short methyl arm instead of the substituted aromatic side chain in all Series-III members. Additionally, the 5-chloro substituent on the pyrazole ring exhibited great solvent exposure suggesting high solvation penalty (Figure 10D).

### 2.5. Solid Lipid Nanoparticle Formulation Studies

In an attempt to the clinical suitability of **8b**, the *in-silico* pharmacokinetic properties and drug-likeness of compound was investigated using free web-based tool SwissADME (http://www.swissadme.ch/). Notably, the predicted poor water-solubility of **8b** (Log*S*_SILICOS-IT = −7.62; 1.06 × 10^−5^ mg/mL; 2.38 × 10^−8^ mol/L) was identified as a significant parameter that might hinder the compound’s full potential cytotoxic activity. Thus, Solid lipid nanoparticles (SLNs) formulation was adopted as a simple cost-effective approach for optimizing the compound’s kinetic parameters. The main target of SLNs is to enhance the drug absorption, enhance the pharmacological response, and decrease the side effects. SLNs are colloidal dispersions made of solid lipid (possessing high melting point) and a hydrophilic surfactant [43]. SLNs are considered a new generation of submicron-sized lipid emulsions where solid lipids are utilized instead of liquid lipids (oil) [44]. Due to their unique characteristics, such as small size, large surface area, and increased drug loading capacity, SLNs are attracting great attention of formulators world-wide to improve performance and bioavailability of pharmaceuticals [45]. It was reported that SLNs have the ability to release entrapped drug in controlled manner and enhance stability of the entrapped drug [46]. Therefore, formulating **8b** as SLNs was expected to enhance its bioavailability at the tumor site and hence improve its cytotoxic activity.

#### 2.5.1. Design, Preparation and Optimization of Drug-SLN

Eight drug-SLNs formulations were prepared via hot-melting homogenization technique. The formulations were designed by 2^3^-factorial design using Stat-Ease^®^ V.11 software (Design-Expert^TM^; Minneapolis, MN, USA) (Table 2). The selected factors (independent variables) were type of lipid (A; X_1_) and Surfactant (B; X_2_), as well as the concentration of surfactant (C; X_3_). According to the adopted 2^3^-factorial design, eight formulations were prepared and evaluated for encapsulation efficiency (Y_1_: EE%), particle size (Y_2_: PS) and zeta potential (Y_3_: ZP). Compositions of eight drug-SLNs formulations are presented in Table 3. Drug analysis at different concentration was done using HPLC at λ_max_ 254 nm, showing linear relationship between the drug concentration and peak area, obeying Beer-Lambert’s law (R^2^ = 0.999) (Supplementary Materials; Figure S1).

#### 2.5.2. The Effect of Formulation Factors in the Responses 

The prepared formulations of drug-SLNs were evaluated for the preselected responses; Encapsulation Efficiency (Y1: EE%), the particle size (Y2: PS) and zeta potential (Y3: ZP). As represented in Table 4, it was found that there was a difference in the results of the responses which gave an indication that the formulation factors have a great effect of the responses.

With the prepared formulations of drug-SLNs, significant impact on the preselected responses was depicted in response to the formulation factors. The EE% of all prepared drug-SLNs ranged from 37.1 ± 2.45% for F1 to 95.3 ± 1.34% for F3. As represented by model Equation (1), the EE% was increased by increasing the level of X1 and X2 from −1 to +1. These results infer that the formulations prepared by GMS and Poloxamer 188 had a higher EE% than those prepared using COMP or Cremophor RH40. Moreover, EE% showed an inverse relationship with X3: surfactant concentration where higher EE% was seen with 1.5% *w*/*v* surfactant.

Y_1_ (EE%) = 76.31 + 8.16 X_1_ + 16.06 X_2_ − 5.21 X_3_ (1)

**Figure 12:**
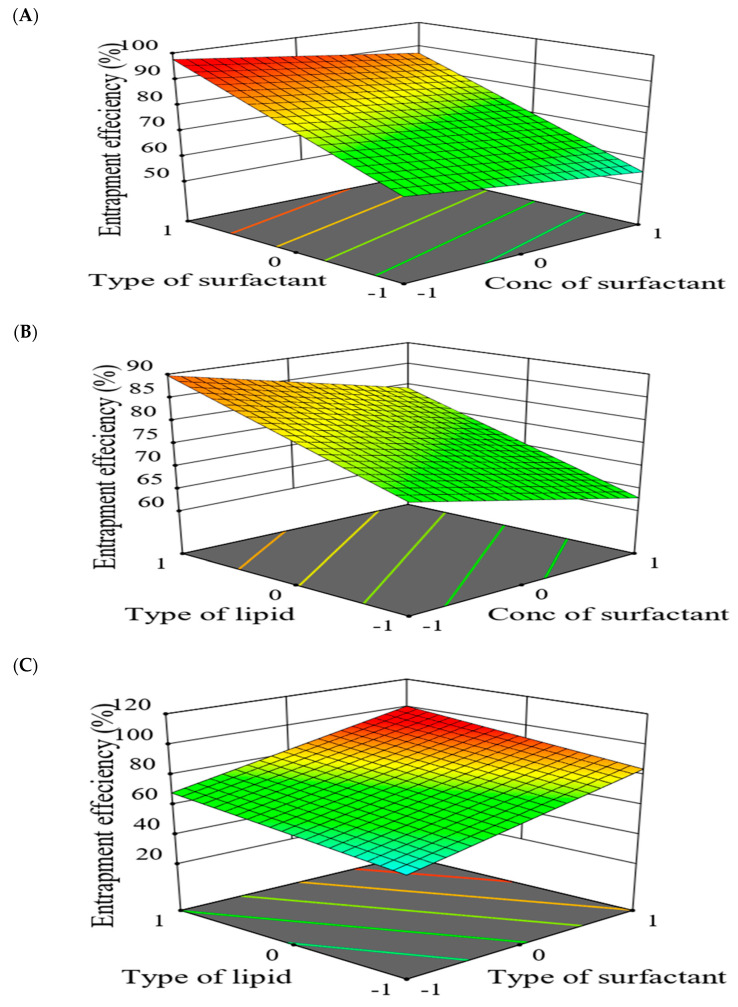
3D-response surface plots showing combined effect of formulation factors on encapsulation efficiency (EE%; Y_1_). The adopted 2^3^ model analysis evaluated two variable parameters while keeping (**A**) type of lipid (X_1_); (**B**) type of surfactant (X_2_); (**C**) concentration of surfactant (X_3_) constant.

For better illustration, a 3D-response surface plot showing the impact of formulation factors on EE% was constructed (Figure 12). Interestingly, GMS-prepared SLNs exhibited higher EE% than those constituted by COMP. These findings might be for the large encapsulation space in case of GMS resulting from less-ordered SLNs structure by the virtue of their long carbon chains (C21) [10,30]. Additionally, higher EE% was assigned for Poloxamer 188-prepared SLNs as compared to Cremophor RH40 which may be correlated to the higher hydrophilic-lipophilic balance (HLB) value proposed by Poloxamer 188 [47]. Latter findings are in good agreement with Qushawy et al., where the prepared carbamazepine-SLNs depicted increased EE% through using GMS and Poloxamer 188 as solid lipid and surfactant, respectively [48]. The increase of surfactant concentration from 1% to 1.5% resulted in decreased EE% which may be for increased drug solubility within the aqueous phase [49]. The same results were depicted by Joseph et al. where olanzapine-SLNs EE% showed an inverse relationship with surfactant concentration [50].

**Figure 13:**
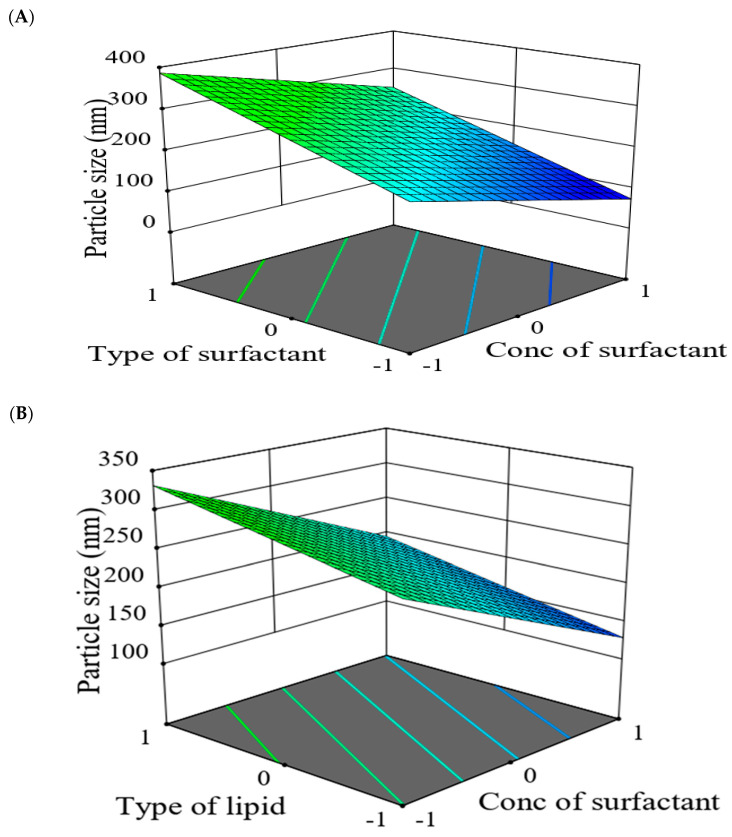

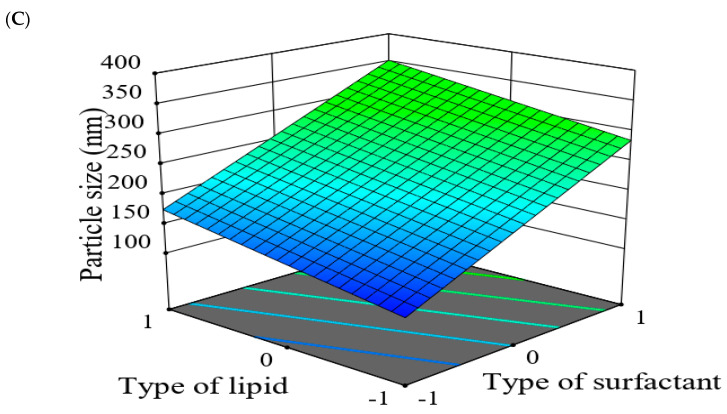
3D-response surface plots showing combined effect of formulation factors on particle size (PP; Y_2_). The adopted 2^3^ model analysis evaluated two variable parameters while keeping (**A**) type of lipid (X_1_); (**B**) type of surfactant (X_2_); (**C**) concentration of surfactant (X_3_) constant.

Regarding the PS of all prepared drug-SLNs, values ranged from 135.1 ± 1.0 nm for F2 to 537.3 ± 10.4 nm for F7. The model Equation (2) of PS, revealed that PS had a direct relationship with X1 and X2 while, an inverse relationship with X3. As shown by Figure 13, the 3D response surface plot studied the impact of formulation factors (X1, X2 and X3) on PS of the prepared SLNs. It was found that PS was increased in case of CMS than in case of COMP which might be correlated to the fact of using solid lipid with high melting point, resulted in slow crystallization and large particle size [51]. The results in good agreement with Priyanka and Hasan found that the particle size of prepared montelukast SLNs was influenced by lipid type and the decreasing order of particle size for the three lipids was Compritol < GMS < stearic acid [52].

Y2 (PS) = 250.06 + 33.34 X_1_ + 88.51 X_2_ − 67.84 X_3_ (2)

The PS of the prepared SLNs was also affected by surfactant type, where using Poloxamer 188 resulted in SLNs larger sizes than those prepared by Cremophor RH40. This may be reasoned to the conception of using surfactant with higher HLB can result in preparation of SLNs with larger size [53]. Additionally, as the surfactant concentration elevated from 1% to 1.5%, the particle size of SLNs decreased. This might be assigned to reduction within interfacial tension between the emulsion phases by increasing the concentration of surfactant which led to smaller PS of SLNs after congealing [50,52].

Moving towards the final parameter, The ZP of all prepared drug-SLNs was ranged from −12.3 ± 0.77 mV for F1 to −39.4 ± 0.095 mV for F6. zeta potential (ZP) is defined as potential difference existing within the stationary layer between the dispersion medium and the dispersed phase (solid particles) [54]. The value of ZP provides indication for the preparation stability, where higher values correlate to higher formulation stability [55]. Within the presented model Equation (3), the ZP of prepared drug-SLNs increased by increasing the level of lipid and surfactant types yet decreased by increasing the level of surfactant concentration.

Y_3_ (ZP) = 25.79 + 9.79 X_1_ + 0.4375 X_2_ − 1.44 X_3_ (3)

**Figure 14:**
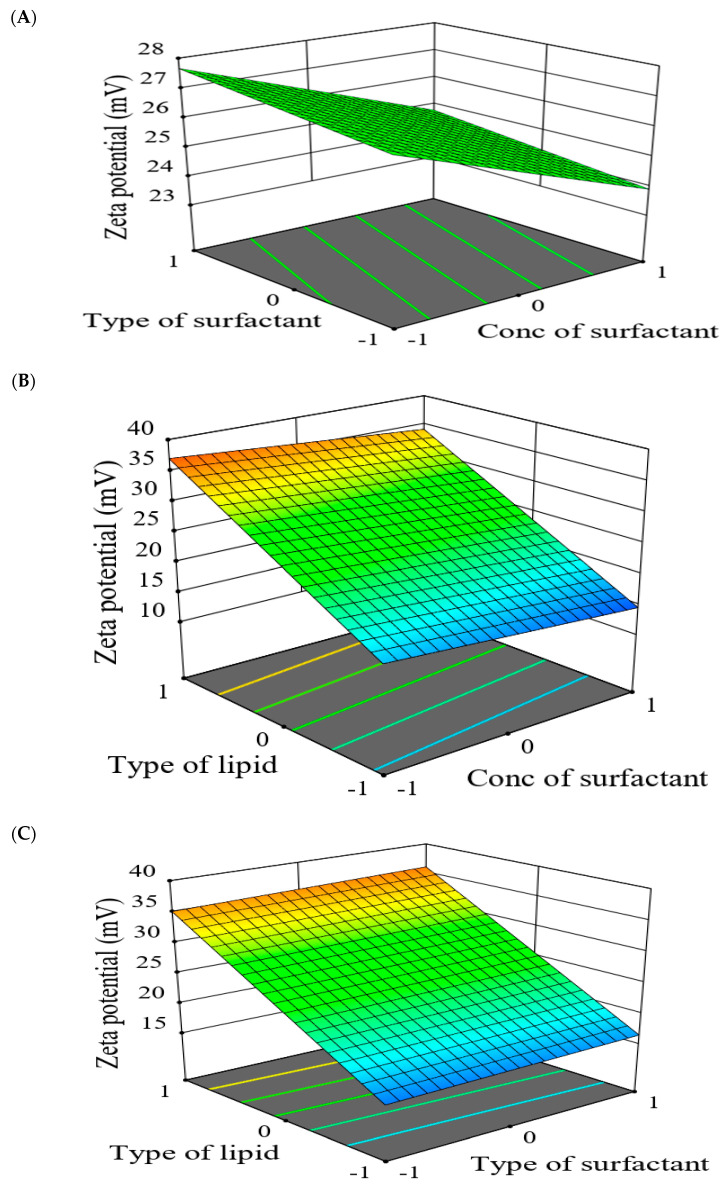
3D-response surface plots showing combined effect of formulation factors on zeta potential (ZP; Y_3_). The adopted model analysis evaluated two variable parameters while keeping (**A**) type of lipid (X_1_); (**B**) type of surfactant (X_2_); (**C**) concentration of surfactant (X_3_) constant.

Corresponding 3D-response surface plot showed the effect factors on ZP responses (Figure 14). The type of lipid had a significant impact on negative value of ZP. The value was increased by using GMS than in case of COMP which might be related to longer carbon-chain of GMS resulted in the larger size of SLNs and larger surface area. It was found that negative zeta potential of the prepared SLNs was slightly decreased by using Cremophor RH40 while increased in case of Poloxamer 188 which may be attributed to differences within respective HLB values [56]. Moreover, ZP was decreased with increased surfactant concentration which may be due to the masking of surface charge by increasing the surfactant concentration [50,57]. It worth mentioning that the PDI values of all prepared drug-SLNs ranged from 0.282 ± 0.01 to 0.639 ± 0.12 for F2 and F8, respectively. Findings from PDI values indicated a narrow size distribution [54].

#### 2.5.3. Optimization of Formulation Variables to Select the Best Formula

**Figure 15:**
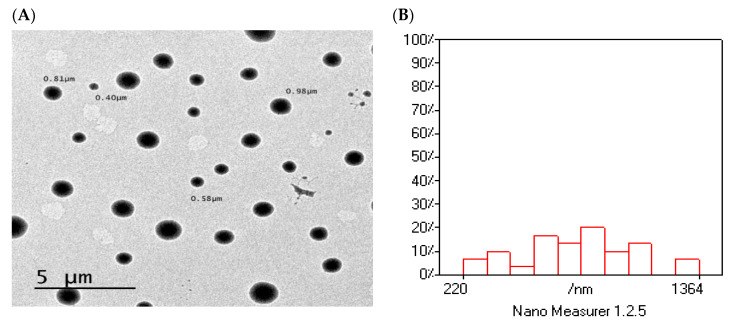
The surface morphology of the optimized formulation (F8). (**A**) TEM image; (**B**) size distribution analysis of TEM image.

Purpose of optimization was to maximize EE%, minimize PS, as well as maximize values of the ZP. The Design-Expert V.11 software was used for obtaining an optimum level of each single formulation factor to achieve the desired goals for every response and obtain the optimized formulation of drug-SLNs. According to 2^3^-factorial design, formulation F8 was the optimized formulation in which prepared using GMS as a solid lipid and poloxamer 188 as a surfactant in concentration 1.5%. The predicted values of responses for the optimized formulation were 95.325% for EE% (Y1), 284.075 nm for PS (Y2), and −34.575 mV for ZP (Y3). Interestingly, these predicted values were close to the actual values of responses with desirability value 0.781 which indicate the validity of 2^3^-factorial design. The transmission electron microscopic (TEM) image of F8 illustrated the spherical shape of the prepared SLNs within the nano size (Figure 15A). Size analysis for TEM image, using Nano-Measurer^®^ V.1.2 software (Shanghai, China), exhibited narrow size distribution (Figure 15B). These findings were in good agreement with Qushawy et al. preparing carbamazepine-SLNs using GMS and stearic acid where the prepared SLNs were spherical in shape with nano size [44].

#### 2.5.4. In-Vitro Release Study of Optimized Formulation (F8) in Comparison with **8b**

**Figure 16:**
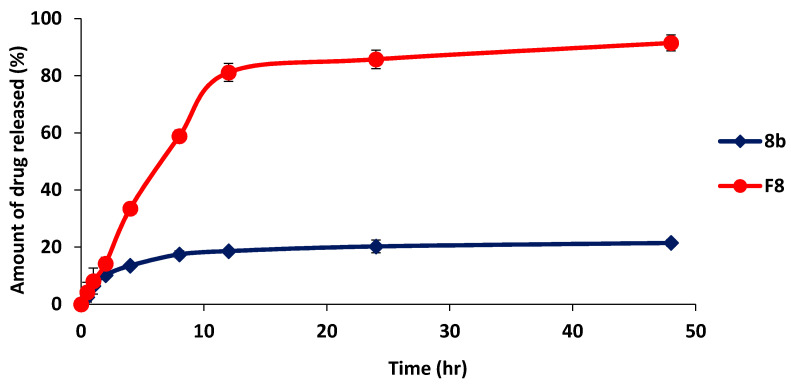
In-vitro drug release analysis of drug-optimized SLNs formula (F8) in comparison with **8b** as free drug form. All data are represented as mean of three independent experiments ± SEM.

The in-vitro drug release profile of drug-SLNs was conducted for evaluating the stability and release behavior of drug-SLNs. The drug release from F8 was prolonged over 48 h, where the total released amount of drug from F8 was 91.47 ± 2.78%, while being 21.48 ± 1.17% for **8b** (*p* < 0.001) (Figure 16). Thus, drug-SLNs may be expected to assist as stable nanoparticles for prolonged time and help in increasing the accumulation of drug in tumor site [58]. These results may be attributed to presence of the drug in more solubilized form and the prolonged effect might be the result of the drug diffusion from the lipid matrix [59].

#### 2.5.5. Cytotoxicity Study of Optimized Formulation

**Figure 17:**
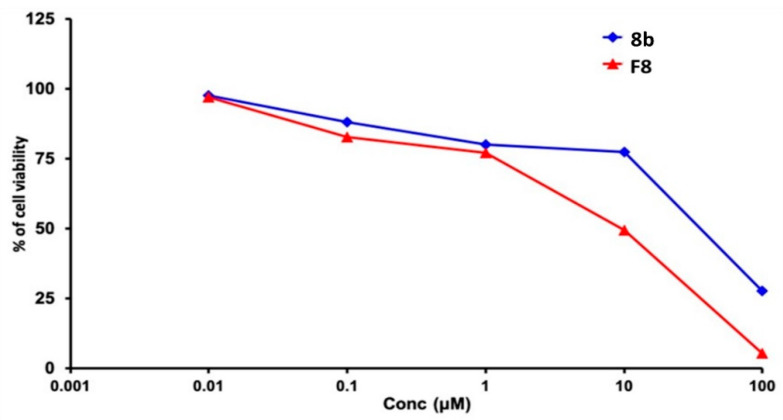
The Sulforhodamine B (SRB) cytotoxicity assay of the tested **8b** as compared to drug-optimized SLNs formula (F8) on MDA-MB-231/breast cancer cells. At 5 × 10^3^ cells/well cell density, cells were treated with various 8b concentrations (0.01, 0.1, 1, 10, and 100 µM) for 72 h exposure time frame. Cytotoxic effect was detected via protein-bound SRB colorimetric assay (540 nm) and % cell growth viability was estimated in triplicates as compared to vehicle negative controls representing the untreated cells.

The cytotoxic efficacy of compound **8b** was assayed in its pure form and in optimized SLN formulation (F8). From the Sulforhodamine B (SRB) assay, the compound’s IC_50_ within MDA-MB-231/breast cancer cell line was significantly decreased from 12.43 ± 0.50 μM to 9.27 ± 0.34 μM for **8b** and formulated form, respectively (*p* < 0.05) (Figure 17). Based on the fact that **8b** is lipophilic (Consensus Log*P_o/w_* = 5.16) with poor aqueous solubility, it was predicted to possess low bioavailability [60]. Incorporation of the drug into lipid-based nanoparticles might offer significant improvement in its anticancer efficacy as the lipid nature of the SLN increases its solubility and allows the presence of the drug in its amorphous form. The latter might cause an increase within the drug **8b** penetration into the tumor cells [61]. Wang and colleagues found similar results when evaluated the cytotoxic effect of Resveratrol on MDA-MB-231/breast cancer cell line [62]. The authors mentioned that the enhanced cytotoxic effect of Resveratrol SLNs as compared to pure resveratrol may be due to the carrier hydrophobic nature facilitating the intra-cellular uptake.

## 3. Materials and Methods

### 3.1. General Experimental

All chemicals, reagents and solvents were purchased from Sigma-Aldrich, Fisher Scientific, Alfa-Aesar, Fluka and Acros Chemicals. Whenever required, solvents were dried prior to use as described by the handbook Purification of Laboratory Chemicals and stored over 4Å molecular sieves under nitrogen. Flash column chromatography was performed with silica gel (230–400 mesh) (Merck) and TLC was performed on pre-coated silica gel plates (Merck Kiesel gel 60F254, BDH). Melting points were determined on an electrothermal instrument (Gallenkamp) and are uncorrected. Compounds were visualized by irradiation with UV light at 254 nm and 365 nm. The NMR spectra of all new compounds were recorded on a Bruker AVANCE DPX500 spectrometer operating at 500 and 125 MHz for ^1^H and ^13^C NMR, respectively, and auto calibrated to the deuterated solvent reference peak (Supplementary Materials; Figure S2). Chemical shifts are given in *δ* relative to tetramethylsilane (TMS); the coupling constants (*J*) are given in Hertz. TMS was used as an internal standard (*δ* = 0 ppm) for ^1^H NMR and CDCl_3_ served as an internal standard (*δ* = 77.0 ppm) for ^13^C NMR. Multiplicity is denoted as s (singlet), d (doublet), t (triplet), q (quartet), m (multiplet) or combinations thereof. The compounds imines 11a–f and aldehydes 12a–f were prepared according to Kishk et al. [29]. The DI Analysis Shimadzu QP2010-Plus^®^ GC/MS (Shimadzu^TM^, Tokyo, Japan) was adopted for recording the low-resolution mass spectra (MS) of the synthesized compounds at electron impact (EI^+^) mode. Regarding purity analysis, the elemental analyses were recorded on Vario^®^ EL-CHNS Elemental Analyzer (GmbH^TM^, Hanau, Germany). The results of elemental analyses (C, H, N) were found to be in good agreement (±0.45%) with the calculated values. All compounds were >95% pure.

### 3.2. Chemical Synthesis 

#### 3.2.1. Synthesis of 2-(chloromethyl)-1H-Benzimidazole (**2**) 

A mixture of ortho-phenylene diamine (1.0 g, 9.25 mmol) and chloroacetic acid (1.32 g, 13.97 mmol) in 4 N HCl (60 mL) was refluxed for 24 h. The purified product was obtained by re-crystallization from water after neutralization with 6 N NH_4_OH, yield 0.4 g (40%). The product was used in the following step without further purification [63]. M.p. 149–151 °C (Lit. 147.8–148.2 °C). ^1^H NMR (DMSO-d_6_): *δ* 4.91 (s, 2H, CH_2_), 7.25–7.29 (m, 2H, CH, Ar), and 7.57–7.59 (m, 2H, CH, Ar).

#### 3.2.2. Synthesis of 2-Aminomethyl-Benzimidazole Derivatives (**3a**–**d**) 

A mixture of compound (**2**) (1 mmol) and the appropriate aromatic amine (1 mmol) in the presence of potassium iodide (1 mmol) were refluxed in absolute ethanol for 6 h, then potassium iodide (1 mmol) dissolved in 5 mL water was added and refluxed overnight. After cooling to room temperature, the solution was poured into ice, filtered and crude product was purified by flash column chromatography using gradient EtOAc: *n*-hexane [26].

*N*-((1*H*-benzo[*d*]imidazol-2-yl)methyl)-3-methylaniline (**3a**).

Prepared from 3-methylaniline, yield 0.07 g (71%), as an off-white powder. M.p. 139–141 °C. TLC (EtOAc: *n*-Hexane 2:3), R*_f_*: 0.23. ^1^H NMR (DMSO-d_6_): *δ* 2.39 (m, 3H, CH_3_), 4.46 (d, *J* = 5.38 Hz, 2H, CH_2_), 6.17 (t, *J* = 7.2 Hz, 1H, NH), 6.50 (s, 1H, NH), 6.96 (s, 1H, CH, Ar), 7.28 (d, *J* = 7.2 Hz, 2H, CH, Ar), 7.49 (m, 1H, CH, Ar), 7.56 (t, *J* = 7.4 Hz, 2H, CH, Ar), 7.76 (d, *J* = 7.4 Hz, 2H, CH, Ar). ^13^C NMR (DMSO-d_6_): *δ* 21.79 (CH_3_), 42.29 (CH_2_), 110 (2 × CH, Ar), 113.6 (2 × CH, Ar), 117.8 (CH, Ar), 129.2 (2 × CH, Ar), 129.75 (CH, Ar), 138.34 (3C, Ar), 143.4 (C, Ar). MS (EI^+^) *m*/*z*: 237.38 [M^+^]. Anal. Calcd for C_15_H_15_N_3_ (237.31): C, 75.92; H, 6.38; N, 17.72. Found: C, 75.81; H, 6.22; N, 17.60.

*N*-((1*H*-benzo[*d*]imidazol-2-yl)methyl)-2,6-dimethylaniline (**3b**). 

Prepared from 3,5-dimethylaniline, yield 0.06 g (61%), as an off-white powder. M.p.159–161 °C. TLC (EtOAc: *n*-Hexane 1:2), R*_f_*: 0.5. ^1^H NMR (DMSO-d_6_): *δ* 2.30 (m, 6H, 2 × CH_3_), 4.36 (s, 2H, CH_2_), 4.54 (s, 1H, NH), 6.81–6.89 (m, 1H, Ar), 6.94 (d, *J* = 7.34 Hz, 2H, Ar), 7.16–7.19 (m, 2H, Ar), 7.54 (d, *J* = 7.5 Hz, 2H, Ar). ^13^C NMR (DMSO-d_6_): *δ* 18.9 (2 × CH_3_), 46.2 (CH_2_), 111.8 (2 × CH, Ar), 118.8 (CH, Ar), 122 (2 × CH, Ar), 129.3 (2 × CH, Ar), 129.6 (2C, Ar), 138.8 (2C, Ar), 146.1 (C, Ar), 154.4 (C, Ar). MS (EI^+^) *m*/*z*: 251.17 [M^+^]. Anal. Calcd for C_16_H_17_N_3_ (251.33): C, 76.46; H, 6.82; N, 16.72. Found: C, 76.37; H, 6.89; N, 16.73

*N*-((1*H*-benzo[*d*]imidazol-2-yl)methyl)-3-bromoaniline (**3c**) [64]. 

Prepared from 3-bromoaniline, yield 0.07 g (73%), as a light brown powder. M.p. 139–141 °C. TLC (EtOAc: *n*-hexane 1:2), R*_f_* 0.27. ^1^H NMR (DMSO-d_6_): *δ* 4.16 (s, 2H, CH_2_), 7.21–7.24 (m, 2H, Ar), 7.57 (d, *J* = 7.4 Hz, 2H, Ar), 7.59 (s, 1H, NH), 7.75 (d, *J* = 7.5 Hz, 1H, Ar), 7.91–8.12 (m, 3H, Ar). ^13^C NMR (DMSO-d*_6_*): δ 41.9 (CH_2_), 111.7 (2 × CH, Ar), 115.1 (2 × CH, Ar), 119.1 (CH, Ar), 121.8 (CH, Ar), 122.7 (2 × CH, Ar), 131.1 (1C, Ar), 150.5 (2C, Ar), 153.5 (1C, Ar), 169. (1C, Ar). MS (EI^+^) *m*/*z*: 302.72 [M^+^]. Anal. Calcd for C_14_H_12_BrN_3_ (302.18): C, 55.65; H, 4.01; N, 13.92. Found: C, 55.62; H, 3.93; N, 13.51.

4-(((1*H*-benzo[*d*]imidazol-2-yl)methyl)amino)phenol (**3d**). 

Prepared from 4-hydroxyaniline, yield 0.05 g (51%), as a brown powder. M.p.139–141 °C. TLC (EtOAc: *n*-hexane 1:2), R*_f_* 0.4. ^1^H NMR (DMSO-d_6_): *δ* 4.14 (s, 2H, CH_2_), 7.22 (d, *J* = 7.5 Hz, 2H, Ar), 7.32 (d, *J *= 7.4 Hz, 2H, Ar), 7.47–7.49 (m, 1H, CH, Ar), 7.57 (d, *J* = 7.5 Hz, 2H, Ar), 7.59 (s, 1H, NH), 7.75 (d, *J* = 7.5 Hz, 1H, Ar), 9.45 (s, 1H, OH), 12.18 (s, 1H, NH-indole). ^13^C NMR (DMSO-d*_6_*): *δ* 43.6 (CH_2_), 115.2 (2 × CH, Ar), 116.3 (2 × CH, Ar), 116.7 (2 × CH, Ar), 124.1 (2 × CH, Ar), 139.1 (2C, Ar), 141.5 (1C, Ar), 142.1 (1C, Ar), 146.9 (1C, Ar). MS (EI^+^) *m*/*z*: 239.11 [M^+^]. Anal. Calcd for C_14_H_13_N_3_O (239.28): C, 70.28; H, 5.48; N, 17.56. Found: C, 70.29; H, 5.49; N, 17.45.

#### 3.2.3. Synthesis of Ethyl, 1H-Indole-2-Carboxylate (**5**) 

To a stirred solution of 1*H*-indole-2-carboxylic acid (**4**) (1.5 g, 9.31 mmol) in dry ethanol (25 mL), sulfuric acid (0.5 mL) was added as catalyst. The reaction mixture was refluxed for 1.5 h. After the reaction was complete, water (25 mL) was added, and the mixture was extracted with ethyl acetate (3 × 50 mL). The combined organic phases were washed with brine, water, and dried over anhydrous Na_2_SO_4_. The solvent was evaporated under vacuum to give ethyl-1*H*-indole-2-carboxylate (**6**) as a white powder [65]. M.p. 126–127 °C (Lit. M.p. 123–124 °C). ^1^H NMR (CDCl_3_) *δ* 1.43 (t, *J* = 7.4 Hz, 3H, CH_3_), 4.51 (q, *J* = 7.2 Hz, 2H, CH_2_), 6.96–6.99 (m, 1H, Ar), 7.12 (d, *J* = 7.0 Hz, 2H, Ar), 7.35 (d, *J* = 7.2 Hz, 2H, Ar), 9.31 (s, 1H, NH).

#### 3.2.4. Synthesis of 1H-Indole-2-Carbohydrazide (**6**) 

To a stirred solution of ethyl 1*H*-indole-2-carboxylate (**5**) (0.46 g, 7.93 mmol) was added hydrazine monohydrate (4 mL, 79.90 mmol) in the presence of 15 mL of absolute ethanol. The reaction mixture was refluxed for 6 h. After the reaction was complete, the solution was evaporated under vacuum, the remaining residue after evaporation (0.50 g) was washed with dichloromethane (0.5 mL × 3). The product was obtained as colorless crystals and was used directly in the following reaction without any further purifications [66]. M.p. 245–246 °C (Lit. M.p. 247–248 °C), ^1^H-NMR (DMSO-d_6_): *δ* 4.49 (s, 2H, NH_2_), 7.13 (d, *J* = 4.6 Hz, 1H, Ar), 7.19 (s, 1H, Ar), 7.25 (d, *J* = 7.4 Hz, 1H, Ar), 7.49 (d, *J* = 7.4 Hz, 1H, Ar), 7.65 (d, *J* = 7.5 Hz, 1H, Ar), 9.79 (s, 1H, NH), 11.68 (s, 1H, NH, indole).

#### 3.2.5. Synthesis of Different Carbohydrazide Derivatives (**7**, **8**)

Equimolar amount of appropriate aromatic aldehyde was added to a solution of the hydrazide compound (**6**) (10 mmol) in absolute ethanol (5 mL), in presence of catalytic amount of glacial acetic acid (0.4 mL). Reaction mixture was allowed to reflux with continuous stirring for about 1.5 h and poured into ice/water mixture. The precipitate was filtered, washed with cold water and purified by flash column chromatography using gradient elution of EtOAc: *n*-hexane to give the corresponding carbohydrazide [67,68,69].

*N′*-benzylidene-1*H*-indole-2-carbohydrazide (**7a**) [67]. 

Prepared from benzaldehyde, yield 0.045 g (45%), as an off-white powder. M.p. 111–113 °C. TLC (EtOAc: *n*-Hexane 1:2), R*_f_*: 0.40. ^1^H NMR (DMSO-d_6_): *δ* 7.08 (d, *J* = 7.34 Hz, 2H, CH, Ar), 7.28 (s, 1H, CH, indole), 7.54–7.59 (m, 5H, CH, Ar), 7.78 (d, *J* = 6.11 Hz, 2H, CH, Ar), 8.47 (s, 1H, CH=N), 11.76 (s, 1H, NH, indole), 11.92 (s, 1H, NH, amide). ^13^C NMR (DMSO-d_6_): *δ* 111.1 (CH, Ar), 114.9 (CH, Ar), 119.8 (CH, Ar), 120.7 (CH, Ar), 121.7 (CH, Ar), 128.8 (2 × CH, Ar), 129.3 (2 × CH, Ar), 131.3 (C, Ar), 133.7 (C, Ar), 138.5 (C, Ar), 139.8 (C, Ar), 146.8 (2 × CH, Ar), 157.6 (C=O). MS (EI^+^) *m*/*z*: 263.55 [M^+^]. Anal. Calcd for C_16_H_13_N_3_O (263.30): C, 72.99; H, 4.98; N, 15.96. Found: C, 72.92; H, 5.03; N, 16.14

*N′*-(4-methoxybenzylidene)-1*H*-indole-2-carbohydrazide (**7b**) [69]. 

Prepared from 4-methoxybenzaldehyde, yield 0.03 g (30%), as an off-white powder. M.p. 104–106 °C (Lit. M.p. 98.9 °C). TLC (EtOAc: *n*-Hexane 1:2), R*_f_*: 0.35. ^1^H NMR (DMSO-d_6_): *δ* 4.33 (s, 3H, CH_3_), 7.18 (d, *J* = 7.33 Hz, 2H, CH, Ar), 7.29 (s, 1H, CH, indole), 7.56–7.63 (m, 5H, CH, Ar), 7.82 (d, *J* = 6.15 Hz, 2H, CH, Ar), 8.49 (s, 1H, CH=N), 11.79 (s, 1H, NH, indole), 11.97 (s, 1H, NH, amide). ^13^C NMR (DMSO-d_6_): *δ* 55.3 (CH_3_), 111.3 (CH, Ar), 115.2 (CH, Ar), 120.1 (CH, Ar), 120.8 (CH, Ar), 121.6 (CH, Ar), 129.1 (2 × CH, Ar), 129.5 (2 × CH, Ar), 131.7 (C, Ar), 133.5 (C, Ar), 138.4 (C, Ar), 140.1 (C, Ar), 146.5 (2 × CH, Ar), 157.4 (C=O). MS (EI^+^) *m*/*z*: 293.87 [M^+^]. Anal. Calcd for C_17_H_15_N_3_O_2_ (293.30): C, 69.61; H, 5.15; N, 14.30. Found: C, 69.9; H, 5.11; N, 14.35

*N′*-(4-(dimethylamino)benzylidene)-1*H*-indole-2-carbohydrazide (**7c**) [69]. 

Prepared from 4-(dimethylamino)benzaldehyde, yield 0.04 g (35%), as a yellow powder. M.p. 90–92 °C (Lit. M.p. 82 °C). TLC (EtOAc: *n*-Hexane 1:2), R*_f_*: 0.35. ^1^H NMR (DMSO-d_6_): *δ* 3.05 (m, 6H, 2 × CH_3_), 6.77 (t, *J* = 7.33 Hz, 1H, CH, Ar), 7.07 (s, 1H, CH, indole), 7.19 (d, *J* = 7.34 Hz, 2H, CH, Ar), 7.24 (d, *J* = 7.34 Hz, 2H, CH, Ar), 7.55 (d, *J* = 7.35 Hz, 2H, CH, Ar), 7.63–7.65 (m, 1H, CH, Ar), 8.31 (s, 1H, CH=N), 11.64 (s, 1H, NH, indole), 11.82 (s, 1H, NH, amide). ^13^C NMR (DMSO-d_6_): *δ* 41.3 (2 × CH_3_), 103.1 (CH, Ar), 111.8 (2 × CH, Ar), 112.3 (CH, Ar), 119.9 (CH, Ar), 121.6 (CH, Ar), 121.8 (CH, Ar), 123.6 (C, Ar), 127.1 (CH, Ar), 128.4 (CH, Ar), 130.4 (C, Ar), 136.8 (2C, Ar), 148.1 (CH, Ar), 151.4 (C, Ar), 157.2 (C=O). MS (EI^+^) *m*/*z*: 306.82 [M^+^]. Anal. Calcd for C_18_H_18_N_4_O (306.37): C, 70.57; H, 5.92; N, 18.29. Found: C, 70.51; H, 5.94; N, 17.91. 

*N′*-(pyridin-3-ylmethylene)-1*H*-indole-2-carbohydrazide (**7d**) [68]. 

Prepared from nicotinaldehyde, yield 0.03 g (29%), as an off-white powder. M.p. 252–254 °C (Lit. M.p. 250–251 °C). TLC (EtOAc: *n*-Hexane 1:2), R*_f_*: 0.29. ^1^H NMR (DMSO-d_6_): *δ* 6.23 (t, *J* = 7.56 Hz, 2H, CH, Ar), 7.44–7.64 (m, 4H, CH, Ar), 7.33 (d, *J* = 7.56 Hz, 1H, CH, Ar), 7.66 (s, 1H, CH, Ar), 7.78 (d, *J* = 7.44 Hz, 2H), 8.34 (s, 1H, CH=N), 11.66 (s, 1H, NH, indole), 11.83 (s, 1H, NH, amide). ^13^C NMR (DMSO-d_6_): *δ* 103.8 (CH, Ar), 112.4 (CH, Ar), 120.1 (CH, Ar), 121.9 (CH, Ar), 124.1 (CH, Ar), 126.9 (CH, Ar), 129.8 (C, Ar), 130.2 (C, Ar), 133.4 (CH, Ar), 136.9 (C, Ar), 144.3 (C, Ar), 148.7 (CH, Ar), 150.7 (2 × CH, Ar), 157.7 (C=O). MS (EI^+^) *m*/*z*: 264.75 [M^+^]. Anal. Calcd for C_15_H_12_N_4_O (264.29): C, 68.17; H, 4.58; N, 21.2. Found: C, 68.21; H, 4.55; N, 20.90.

*N′*-((3-(4-ethylphenyl)-1-phenyl-1*H*-pyrazol-4-yl)methylene)-1*H*-indole-2-carbohydrazide (**8a**). 

Prepared from 3-(4-ethylphenyl)-1-phenyl-1*H*-pyrazole-4-carbaldehyde (**12a**), yield 0.04 g (41%), as an off-white powder. M.p. 254–256 °C. TLC (EtOAc: *n*-Hexane 1:1), R*_f_*: 0.42. ^1^H NMR (DMSO-d_6_): *δ* 1.24 (t, *J* = 7.56 Hz, 3H, CH_3_), 2.69 (q, *J* = 7.56 Hz, 2H, CH_2_), 6.23 (t, *J* = 7.22 Hz, 1H, Ar), 6.35 (t, 1H, Ar), 6.43 (s, 1H, Ar), 6.51–6.59 (m, 3H, CH, Ar), 6.71 (d, *J* = 8.25 Hz, 2H, CH, Ar), 6.89 (d, *J* = 7.56 Hz, 2H, CH, Ar), 6.95 (d, *J* = 8.25 Hz, 2H, CH, Ar), 7.20 (d, *J* = 7.56 Hz, 2H, CH, Ar), 7.71 (s, 1H, CH, Ar), 8.33 (s, 1H, CH=N), 11.76 (s, 1H, NH, indole), 11.85 (s, 1H, NH, amide). ^13^C NMR (DMSO-d_6_): *δ* 15.6 (CH_3_), 28.2 (CH_2_), 103.2 (CH, Ar), 112.3 (C, Ar), 116.8 (CH, Ar), 118.8 (2 × CH, Ar), 119.9 (CH, Ar), 121.7 (CH, Ar), 123.7 (CH, Ar), 126.8 (2 × CH, Ar), 126.9 (CH, Ar), 128.2 (CH), 128.5 (CH, Ar), 129.4 (3 × CH, Ar), 129.6 (C, Ar), 130.2 (C, Ar), 136.7 (2C, Ar), 139.1 (CH, Ar), 140.4 (C, Ar), 144.3 (C, Ar), 152.1 (C, Ar), 157.3 (C=O). MS (EI^+^) *m*/*z*: 233.14 [M^+^]. Anal. Calcd for C_27_H_23_N_5_O (433.52): C, 74.81; H, 5.84; N, 15.35. Found: C, 74.85; H, 5.49; N, 15.24.

*N′*-((1-phenyl-3-(4-propylphenyl)-1*H*-pyrazol-4-yl)methylene)-1*H*-indole-2-carbohydrazide (**8b**). 

Prepared from 1-phenyl-3-(4-propylphenyl)-1*H*-pyrazole-4-carbaldehyde (**12b**), yield 0.04 g (39%), as an off-white powder. M.p. 219–221 °C. TLC (EtOAc: *n*-Hexane 1:2), R*_f_*: 0.51. ^1^H NMR (DMSO-d_6_): *δ* 0.93 (t, *J* = 7.4 Hz, 3H, CH_3_), 1.65 (sext, *J* = 7.3 Hz, 2H, CH_2_), 2.63 (t, *J* = 7.4 Hz, 2H, CH_2_), 6.22 (t, *J* = 7.56 Hz, 1H, CH, Ar), 6.33 (t, *J* = 7.56 Hz, 1H, CH, Ar), 6.44 (s, 1H, CH, Ar), 6.55–6.61 (m, 3H, CH, Ar), 6.69 (d, *J* = 8.25 Hz, 2H, CH, Ar), 6.75 (t, *J* = 7.90 Hz, 2H, CH, Ar), 6.81 (d, *J* = 8.25 Hz, 2H, CH, Ar), 7.19 (d, *J* = 7.56Hz, 2H, CH, Ar), 7.73 (s, 1H, CH, Ar), 8.33 (s, 1H, CH=N), 11.76 (s, 1H, NH, indole), 11.85 (s, 1H, NH, amide). ^13^C NMR (DMSO-d_6_): *δ* 13.7 (CH_3_), 24.1 (CH_2_), 37.9 (CH_2_), 103.4 (CH, Ar), 112.5 (C, Ar), 116.8 (CH, Ar), 118.8 (2 × CH, Ar), 119.9 (CH, Ar), 121.7 (CH, Ar), 123.8 (CH, Ar), 126.8 (CH, Ar), 126.9 (CH, Ar), 127.1 (CH, Ar), 128.3 (CH), 128.7 (CH, Ar), 129.4 (2 × CH, Ar), 129.5 (2 × CH, Ar), 130.2 (C, Ar), 137.8 (2C, Ar), 139.5 (C, Ar), 140.3 (C, Ar), 143.5 (C, Ar), 152.5 (C, Ar), 156.5 (C=O). MS (EI^+^) *m*/*z*: 447.79 [M^+^]. Anal. Calcd for C_28_H_25_N_5_O (447.54): C, 75.15; H, 5.63; N, 15.65. Found: C, 75.11; H, 5.89; N, 15.64. 

*N′*-((3-(4-isopropylphenyl)-1-phenyl-1*H*-pyrazol-4-yl)methylene)-1*H*-indole-2-carbohydrazide (**8c**). 

Prepared from 3-(4-isopropylphenyl)-1-phenyl-1*H*-pyrazole-4-carbaldehyde (**12c**), yield 0.03 g (39%), as an off-white powder. M.p. 219–221 °C. TLC (EtOAc: *n*-Hexane 1:2), R*_f_*: 0.52. ^1^H NMR (DMSO-d_6_): *δ* 1.26 (d, *J* = 6.9 Hz, 6H, 2 × CH_3_), 2.97 (sept, *J* = 6.8 Hz, 1H, CH), 7.07 (t, *J* = 7.56 Hz, 1H, CH, Ar), 7.16 (t, *J* = 7.56 Hz, 1H, CH, Ar), 7.29 (s, 1H, CH, Ar), 7.32–7.39 (m, 3H, CH, Ar), 7.43–7.52 (m, 3H, CH, Ar), 7.61 (d, *J* = 7.61 Hz, 2H, CH, Ar), 7.73 (d, *J* = 7.58 Hz, 2H, CH, Ar), 7.94 (d, *J* = 7.57 Hz, 2H, CH, Ar), 8.52 (s, 1H, CH, Ar), 8.64 (s, 1H, CH=N), 11.79 (s, 1H, NH, indole), 11.93 (s, 1H, NH, amide). ^13^C NMR (DMSO-d_6_): *δ* 23.8 (2 × CH_3_), 33.3 (CH), 103.7 (CH, Ar), 113.1 (C, Ar), 116.9 (CH, Ar), 118.8 (2 × CH, Ar), 119.9 (CH, Ar), 121.1 (CH, Ar), 123.7 (CH, Ar), 126.7 (2 × CH, Ar), 126.9 (2 × CH, Ar), 127.0 (2 × CH, Ar), 128.5 (2 × CH, Ar), 129.6 (C, Ar), 130.1 (C, Ar), 136.8 (2C, Ar), 139.1 (CH, Ar), 140.4 (C, Ar), 149.9 (C, Ar), 153.3 (C, Ar), 157.8 (C=O). MS (EI^+^) *m*/*z*: 447.45 [M^+^]. Anal. Calcd for C_28_H_25_N_5_O (447.54): C, 75.15; H, 5.63; N, 15.65. Found: C, 75.11; H, 5.89; N, 15.85.

*N′*-((3-(4-isobutylphenyl)-1-phenyl-1*H*-pyrazol-4-yl)methylene)-1*H*-indole-2-carbohydrazide (**8d**). 

Prepared from 3-(4-isobutylphenyl)-1-phenyl-1H-pyrazole-4-carbaldehyde (**12d**), yield 0.04 g (42%), as an off-white powder. M.p. 229–231 °C. TLC (EtOAc: Hexane 1:2), R*_f_*: 0.45. ^1^H NMR (DMSO-d_6_): *δ* 0.88 (d, *J* = 6.7 Hz, 6H, 2 × CH_3_) 1.88 (m, 1 H, CH), 2.51 (d, *J* = 6.8 Hz, 2H, CH_2_), 7.07 (t, *J* = 6.6 Hz, 1H, CH, Ar), 7.21 (t, *J* = 7.5 Hz, 1H, CH, Ar), 7.29 (s, 1H, CH, Ar), 7.35 (t, *J* = 6.9 Hz, 2H, CH, Ar), 7.49–7.56 (m, 2H, CH, Ar), 7.63 (d, *J* = 7.4 Hz, 2H, CH, Ar), 7.68–7.72 (m, 3H, CH, Ar), 8.03 (d, *J* = 7.5 Hz, 2H, CH, Ar), 8.53 (s, 1H, CH, Ar), 8.69 (s, 1H, CH=N), 11.78 (s, 1H, NH, indole), 11.92 (s, 1H, NH, amide). ^13^C NMR (DMSO-d_6_): *δ* 22.1 (2 × CH_3_), 29.6 (CH), 44.3 (CH_2_), 103.2 (CH, Ar), 112.4 (C, Ar), 116.8 (CH, Ar), 118.8 (CH, Ar), 119.9 (CH, Ar), 121.7 (CH, Ar), 123.7 (2 × CH, Ar), 126.8 (CH, Ar), 126.9 (CH, Ar), 127 (CH, Ar), 128.2 (CH, Ar), 129.4 (C, Ar), 129.5 (2 × CH, Ar), 129.6 (2 × CH, Ar), 130.1 (C, Ar), 136.8 (C, Ar), 139.1 (C, Ar), 141.3 (C, Ar), 141.8 (CH, Ar), 151.8 (2C, Ar), 157.2 (C=O). MS (EI^+^) *m*/*z*: 461 [M^+^]. Anal. Calcd for C_29_H_27_N_5_O (461.57): C, 75.46; H, 5.90; N, 15.17. Found: C, 75.30; H, 6.11; N, 15.20.

*N′*-((3-(benzo[*d*][1,3]dioxol-5-yl)-1-phenyl-1*H*-pyrazol-4-yl)methylene)-1*H*-indole-2-carbohydrazide (**8e**). 

Prepared from 3-(benzo[*d*][1,3]dioxol-5-yl)-1-phenyl-1*H*-pyrazole-4-carbaldehyde (**12e**), yield 0.03 g (33%), as an off-white powder. M.p. 239–241 °C. TLC (EtOAc: *n*-Hexane 1:2), R*_f_*: 0.35. ^1^H NMR (DMSO-d_6_): *δ* 5.29 (s, 2H, CH_2_), 6.31–6.37 (m, 2H, CH, Ar), 6.49 (s, 1H, CH, Ar), 6.57–6.62 (m, 3H, CH, Ar), 6.62 (d, *J* = 8.25 Hz, 1H, CH, Ar), 6.73 (d, *J* = 7.90 Hz, 4H, CH, Ar), 6.89 (d, *J* = 8.25 Hz, 1H, CH, Ar), 7.20 (s, 1H, CH, Ar), 7.71 (s, 1H, CH, Ar), 8.33 (s, 1H, CH=N), 11.76 (s, 1H, NH, indole), 11.85 (s, 1H, NH, amide). ^13^C NMR (DMSO-d_6_): *δ* 101.3 (CH_2_), 103.3 (CH, Ar), 108.6 (CH, Ar), 108.7 (CH, Ar), 112.3 (C, Ar), 116.7 (2 × CH, Ar), 118.8 (C, Ar), 119.9 (CH, Ar), 121.7 (CH, Ar), 122.5 (CH, Ar), 123.7 (CH, Ar), 124.1 (CH, Ar), 126.9 (2C, Ar), 127.2 (CH, Ar), 127.5 (CH, Ar), 130.2 (CH, Ar), 130.5 (CH, Ar), 136.8 (C, Ar), 139.0 (C, Ar), 140.3 (CH, Ar), 147.6 (C, Ar), 147.7 (C, Ar), 151.6 (C, Ar), 157.2 (C=O). MS (EI^+^) *m*/*z*: 449 [M^+^]. Anal. Calcd for C_29_H_27_N_5_O (461.57): C, 69.48; H, 4.26; N, 15.58. It was Found: H, 4.16; C, 69.03; N, 15.22.

*N′*-((5-chloro-1-(3,4-dinitrophenyl)-3-methyl-1*H*-pyrazol-4-yl)methylene)-1*H*-indole-2-carbohydrazide (**8f**). 

Prepared from 5-chloro-1-(3,4-dinitrophenyl)-3-methyl-1*H*-pyrazole-4-carbaldehyde, yield 0.04 g (37%), as an off-white powder. M.p. 204–206 °C. TLC (EtOAc: *n*-Hexane 1:2), R*_f_*: 0.37. ^1^H NMR (DMSO-d_6_): *δ* 1.65 (s, 3H, CH_3_), 6.24–6.26 (m, 2H, CH, Ar), 6.49 (s, 1H, CH, Ar), 6.59 (d, *J* = 7.4 Hz, 2H, CH, Ar), 6.78 (d, *J* = 8.26 Hz, 2H, CH, Ar), 8.02 (s, 1H, CH, Ar), 8.63 (s, 1H, CH=N), 11.76 (s, 1H, NH, indole), 11.85 (s, 1H, NH, amide). ^13^C NMR (DMSO-d_6_): *δ* 18.4 (CH_3_), 101.8 (CH, Ar), 103.1 (CH, Ar), 112.2 (CH, Ar), 112.3 (C, Ar), 119.6 (C, Ar), 119.8 (C, Ar), 121.3 (CH, Ar), 121.6 (CH, Ar), 123.0 (CH, Ar), 123.6 (CH, Ar), 127.0 (CH, Ar), 127.1 (CH, Ar), 130.1 (C, Ar), 130.4 (C, Ar), 136.2 (C, Ar), 136.6 (C, Ar), 147.7 (C, Ar), 157.3 (C, Ar), 161.3 (C=O). MS (EI^+^) *m*/*z*: 467.79 [M^+^]. Anal. Calcd for C_20_H_14_ClN_7_O_5_ (467.83): C, 51.35; H, 3.02; N, 20.96. Found: C, 51.34; H, 3.04; N, 20.91

### 3.3. MTT-Cell Proliferation Assay and Morphological Evaluation

Evaluating the cytotoxicity of tested compounds on two cancer cell lines (A549/lung and MDA-MB-231/breast adenocarcinoma) and non-cancerous (MDCK/kidney cells) was performed while adopting the formerly described method with small alterations [70]. Cancer cell lines were propagated within Dulbecco’s Modified Eagle Medium-High Glucose (Cat.#: P0103; DMEM_High Glucose with Na.Pyruvate and stable Glutamine, Biowest^TM^, Nuaillé, France), while MDA-MB-231 cells were propagated within RPMI-1640 L-Glutamine medium (Cat.#:12-604F; Lonza-Verviers SPRL^TM^, Verviers, Belgium). The culturing media were supplemented via 1% antibiotic-antimycotic 100X (Cat.#: L0010; Biowest^TM^, Nuaillé, France) and 10% fetal bovine serum (FBS) (Cat.#: EU-000-H; Seralab^TM^, West Sussex, UK). Cells were seeded as triplicates within 96-well plate, at 1 × 10^4^ cells per well density, after being counted and viability checked using the trypan blue staining solution (Cat.# ab233465; Abcam^TM^, Cambridge, MA, USA). Seeded cells were permitted to adhere for 24 h under 5% CO_2_ and at 37 °C incubating conditions. The assigned compounds were dissolved in 500 µL DMSO affording the stock solution (100 mM) being ready for more diluting within the whole medium to obtain the compound’s final concentrations; 0.1, 1, 10, 100 µM for cell treatments. Notably, the final DMSO-culture medium concentration was not allowed to exceed 0.2% (*v*/*v*) [71]. Following 24 h compound-cell treatment, the medium was substituted by fresh one and cells were permitted to develop for 48 h. At four hours prior the end of incubation, 10 μL MTT Sigma-Aldrich^TM^ (5 mg/mL in PBS 1X without magnesium and calcium; Cat.# 17-516F, Lonza-Verviers^TM^, Basel, Switzerland) were added to all wells. Following the complete 48 h incubation, 100 μL DMSO was all added to all wells where they were subsequently centrifuged at 4000 rpm for 5 min allowing the formazan crystals of formazan to precipitate. Color was established and intensities were recorded at 490 nm using Synergy-Neo2^®^ Hybrid MultiMode Plate Reader (BioTek^TM^, Winooski, VT, USA), while subtracting the multi-well plates background absorbance at 690 nm. Percentage cell viability was estimated using the subsequent formula: % cell-viability = (average absorbance of treated wells/average absorbance of controls) × 100.

### 3.4. SRB Cytotoxicity Assay

MDA-MB-231: Breast Cancer cell line was obtained from Nawah^TM^ Scientific Inc., (Cairo, Egypt). Cells were maintained within DMEM provided by 100 units/mL of penicillin, 100 mg/mL streptomycin, and 10% of heat-inactivated FBS within 5% (*v*/*v*) CO_2_ humidified atmosphere (37 °C). The validity of cell line was evaluated adopting the previously reported approach [72]. Briefly, an accurate volume (100 μL) of cell line suspension (5 × 10^3^ cells) was placed in 96-well plates and incubated for 24 h within complete media [73]. Cell line suspensions were then treated with another 100 μL media spiked with various drug concentrations (0.01, 0.1, 1, 10, 100 μM/mL) and kept for 72 h. Then the media were replaced with 150 μL of 10% trichloroacetic acid (TCA) for fixing cells through subsequent 1 h incubation at 4 °C [74]. Following incubation, TCA was removed, and cells were washed via distilled water 5 times. Aliquots of 70 μL SRB solution (0.4% *w*/*v*; Cat.# S1402; Sigma-Aldrich^TM^, Taufkirchen, Germany) were added and cell were then incubated in dark for 10 min at 25 °C. Plates were subjected to triplicate washing via 1% acetic acid and then permitted to be air-dried overnight [62]. Protein-bound SRB stain was extracted by addition of tris base (150 μL, 10 mM) and the absorbance was measured at 540 nm using FLUOstar-Omega^®^ microplate reader (BMG-Labtech^TM^ GmbH, Ortenberg, Germany) [75].

### 3.5. Morphological Evaluation

The impact of this tested final compounds on the morphology of treated MDA-MB-231/breast cancer cell line was investigated, through planting cells within 6-well plates and subsequently incubated with 0.1, 1, 10, 100 µM of **8b** for 24 h. Variations within the cells’ morphology were identified via Olympus^®^-CKX53 Inverted Metallurgical light microscope (Olympus^TM^, Center Valley, PA, USA), snapped by Olympus^®^ Digital Camera, and analyzed by OLYMPUS^®^ Stream image analysis software [76].

### 3.6. Flow Cytometer Analysis

Apoptotic assay and cell cycle analysis were proceeded according to previous literatures [77]. In brief, cells were planted in 1.0 × 10^6^ cells/flask density for 24 h. Subsequently, **8b** was added at its IC_50_ value and incubated for 48 h. Following incubation, MDA-MB-231 cells were trypsinized, harvested, and fixed according to information cited within Annexin^®^ V-FITC Detection Kit (Cat.#: K101-25, BioVision^TM^, Milpitas, CA, USA), for quantifying cell’s DNA contents being treated with **8b** relative to control sample using propidium iodide stain (Cat.#: ab139418; Abcam^TM^, Cambridge, MA, USA). Finally, flow cytometry analysis was done using BD-FACSCalibur^®^ cell analyzer platform (Becton Dickinson-Biosciences-SG^TM^, Singapore, Thailand) to estimate which cell cycle phase, the treated cells would be arrested in, as well as computing the percentage of apoptotic cells.

### 3.7. DNA Fragmentation Assay

DNA isolation was performed through planting MDA-MB-231/breast cells at 0.16x10^6^ density, prior to incubation at 37 °C/humidified 5% CO_2_ overnight [78]. Following 48 h exposure time, cells were harvested, washed and lysed via DNA-extraction buffer at 37 °C overnight. The lysate was incubated after that with 100 μg/mL Micro-pestle^®^ DNase/RNase-free (Cat.#: 9097.1; Carl-Roth^TM^, Karlsruhe, Germany) at 37 °C for 2 h, which was followed by three extraction processes using phenol:CHCl_3_ (1:1 *v*/*v*) (Bioflux^TM^, Selangor, Malaysia). A consequent re-extraction was done using CHCl_3_ and then the organic solvent was centrifuged for 5 min at 12,000 rpm within 4 °C conditions. Extracted DNA was precipitated via ice-cold 3 M sodium acetate and absolute ethanol for 1 h at –20 °C, which was then proceeded through centrifugation (15 min—at 12,000 rpm—4 °C). After washed with 70% ethyl alcohol, DNA pellets were air-dried, dissolved within 40 μL Tris-HCl/EDTA (pH 8.0), and then electrophoresed on 1.5% agarose gel for their final staining using ethidium bromide within Tris/acetate/EDTA buffer. DNA fragments were photographed using GelDoc-Go^®^ System (Bio-Rad^TM^, Hercules, CA, USA).

### 3.8. Real-Time PCR Analysis

Quantitative Real-time PCR was operated on Rotor-Gene Q^®^ PCR system as a reader (Cat.#: 204774; Qiagen^TM^, Milan, Italy) via GenElute^®^ RNA extraction/SIGMA PCR kit (Cat.#: REI10; Qiagen^TM^, Milan, Italy)^86^. Cells were treated with IC_50_ of **8b** for 48 h and total RNA was extracted from the non-treated and treated cells. RNA purity was assessed via Nanodrop^®^ 2000/2000c UV-Vis spectrophotometer (Thermo-Scientific^TM^, Bilbao, Spain). Synthesis of cDNA was proceeded using QuantiNova^®^ Reverse Transcription Kit (Cat.#: RTN30; Qiagen^TM^, Milan, Italy) and the subsequent PCR tests were conducted via single tubes. Specific forward/reverse primer pairs were selected for investigated (*Casp-3*, *-8*, *-9*, *BAX*, and *Bcl2*). Obtained results were expressed within Cycle threshold (Ct) values, while relative quantitation of each measured gene was assessed based on ΔΔCt calculations as represented in Table 5 [79].

### 3.9. ELISA Assay

Quantikine^®^ Colorimetric Sandwich-ELISA Kit (Cat.#: DCTC0; R&D Systems, Minneapolis, MN, USA) was used for quantitating the targeted human cytochrome c protein through immunoassay protocol [80]. Based on manufacturer’s instructions, MDA-MB-231/breast cancer cells were incubated with **8b** at its approximated IC_50_, for 48 h. Following incubation, both non-treated and treated cells were subjected to lysis via cell extraction buffer. Lysates were then diluted by standard diluent buffer over the assay range, and then estimated for human cytochrome-c protein.

### 3.10. Molecular Docking Studies

Molecular docking experiments were performed on the 14 investigated using Molecular Operating Environment (MOE) software as reported in previous work with few modifications [81,82,83]. In brief, ligands were constructed via the MOE “builder” tool, and then proceeded through the minimization step, adopting MMFF94x forcefield and 2000 steps of conjugate-gradient approach till a gradient of 0.001 Kcal/Å was reached 0.1 RMS Kcal/mol/Å^2^. The atomic structure of the target protein was prepared by the MOE through 3D-protonation, at physiological pH (7), temperature of 300 K, and 0.1 mol/L salt within implicit solvent at Generalized-Born/Volumn-Integral implicit solvent model. Moreover, the protein was auto-corrected for partial charges, types of atoms, and bond connectivity [11]. The adopted protocol was proceeded through the rigid receptor docking approach since the RMSD of the superimposed apo (PDB ID: 1g5m) and complexed states of Bcl-2 (PDB ID: 6qgk) showed RMSD of 0.934 Å. Thus, ligand binding would suggest non-significant impact on the protein conformational change either on local or global aspects. Throughout the adopted docking protocol, the ligand conformations were developed through the method of bond rotation, lodged within in the defined active site guided by triangular-matching approach, and then conformations were ranked via the London_dG scores. The top ten docked poses were retained for subsequent refinement and then an energy minimization stage, within the target pocket, before they were rescored using GBVI/WSA forcefield [84]. High docking energy, RMSD values (2.0 Å threshold), and ligand interaction with relevant pocket residues, all were considered for selecting the best docking pose for each investigated ligand. Analysis and visual inspection of ligand-protein interactions was achieved using PyMol v2.0.6 Graphics System (Schrödinger^TM^, NY, USA) [85]. The hydrophobic interactions were determined via MOE ligand interactions tool as well as manual measurements using PyMol bond distance measurement tools.

### 3.11. Preparation of Drug-Loaded SLNs

Eight formulations of drug-SLNs were successfully prepared by the hot melting homogenization technique [48,61]. An accurate amount (450 mg) of selected lipid; either COMP (Cat.#: 3123; Gattefossé^TM^, Lyon, France) or GMS (Cat.#: 23A70; Sasol^TM^, Hamburg, Germany), was melted in a small glass vial at temperature 80 °C which exceed the melting point of both lipid. Consequently, 10 mg of drug was added and dissolved completely forming oily phase. Considering the aqueous phase, it was prepared via addition of accurate amount of surfactant (COMP or Poloxamer 188; Cat.#: 9003-11-6; Sigma-Aldrich^TM^, St. Louis, MO, USA) to distilled water and allowed to heat at the same temperature of oily phase. Subsequently, aqueous phase was added slowly to oily phase with stirring while keeping the temperature to yield coarse emulsion using a hot plate with stirrer (Brandstead/Thermolyne^TM^, Ramsey, MN, USA). The resultant preparation was homogenized at 15,000 rpm for 15 min to form fine o/w emulsion using Heidolph^®^ silent crusher homogenizer (Heidolph^TM^, Schwabach, Germany) [86]. The final preparation was allowed to cool at *r.t.* to solidify the SLNs the stored at refrigerator for further study.

### 3.12. HPLC Analysis

Drug stock solution of 1 mg/mL in mobile phase:Buffer (0.1% Triflouroacetic acid/water) and acetonitrile was prepared, and seven dilutions were prepared in concentrations of 5, 10, 25, 50, 100, 200, and 300 µg/mL. All solutions were filtered using 0.22 µm syringe filter and then 10 µl was subjected to HPLC analysis using Waters-2690 Alliance^®^ HPLC system (Waters^TM^, Milford, MA, USA) [87]. Peak area of drug was noted at 254 nm. Each experiment was performed within a triplicate manner and mean peak area was plotted against the drug concentration.

### 3.13. Determination of the Drug-SLNs Parameters (Y1–3)

For estimating the encapsulation efficiency (EE%; Y1), samples of the eight formulations of drug-SLNs were subjected to vortexing for 1 min followed by centrifugation at 15,000 rpm for 30 min using Biofuge^®^ centrifuge (Primo-Heraeus^TM^, Germany) [61]. For each sample, the clear supernatant collected and filtered using 0.22 µm syringe filter then 10 µl of each sample was injected and subjected to HPLC analysis at 254 nm [62]. Equation (4) was used to calculate the EE% of drug [62,88]. Dynamic light scattering (DLS) technique was applied to determine PS (Y2), ZP (Y3), and PDI of all prepared drug-SLNs using Zetasizer^®^ (Malvern^TM^ Instruments Ltd., Malvern, UK) [86]. The measurements were done at 25 °C after appropriate dilution of each sample [49].

EE% = (Total amount of drug-Unentrapped amount of drug)/(Total amount of drug) × 100(4)

### 3.14. Surface Morphology of Optimized Formulation

Morphology of optimized formulation was determined by JTEM^®^ transmission electron microscope (TEM; model 1010, JEOL^®^, Tokyo, Japan). A small sample of optimized formulation was spread upon collodion-coated copper grid then left to be dried for nearly 2 min allowing the SLNs-collodion adherence [74,89]. The samples were subjected to stain by addition of one drop of uranyl acetate solution. Following drying, samples were examined with TEM.

### 3.15. In-Vitro Release Study of the Optimized Formulation

The in-vitro drug-release behavior of SLNs was determined through the dialysis method [58]. The release of drug from SLNs was done using phosphate buffer saline (pH 7.4) as dissolution medium. An accurate amount of SLNs (equivalent to 5 mg drug) was taken and placed within the dialysis bag (MWCO: 12 kDa). The dialysis bag was closed from both ends and placed within 75 mL Phosphate Buffer Saline (pH 7.4) as dissolution medium. The analysis was conducted while maintaining the dissolution medium at 37 ± 1 °C and being stirred via magnetic stirrer at 100 rpm. Dissolution medium samples (2 mL) were withdrawn at pre-determined time intervals (0.5, 1, 2, 4, 8, 12, 24 and 48 h). Equal volumes of fresh dissolution medium were added to replace the withdrawn volumes for keeping sink condition [90]. The experiment was done in triplicate and the amount of drug release was measured following the same procedure as in the entrapment efficiency section.

## 4. Conclusions

The presented manuscript reported indole- and benzimidazole-based compounds with promising pro-apoptotic activity targeting the Bcl-2 biological targets. The furnished synthesized compounds owned structural diversity and chemical scaffolds that mimic the market released Bcl-2 family pan-antagonist, obatoclax. Either through replacing the pyrrole ring of obatoclax with benzimidazole or extending the structure after the indole ring, fourteen compounds were afforded belonging to three chemical series. Biological evaluation through MTT-proliferation on two solid tumor cell lines, A549/lung and MDA-MB-231/breast adenocarcinoma, provided solid evidence concerning the compounds’ cytotoxic activity. Compound **8b** was considered promising for further evaluation as it possessed impressive balanced cytotoxic/safety profile on non-cancerous MDCK/kidney cell line. Further, cell cycle analysis, DNA fragmentation, apoptotic gene expression, and protein level analysis have confirmed the compound’s promising anti-cancer activity and provided valuable insights regarding its molecular mechanisms. Moreover, a validated molecular docking investigation, on Bcl-2 crystallized protein, has thoroughly investigated the differential compounds’ bindings at the active site, while showing good correlation between the IC_50_ values and furnished docking scores. Finally, the introduction of compound **8b** within a well-optimized solid lipid nanoparticle formulation has improved the compound’s pharmaceutical profile leading to a significant improvement at the compound’s cytotoxic activity. All the above findings have introduced **8b** as a promising anti-cancer lead candidate that is worthy of future fine-tuned lead optimization and development studies and exploration of its potentiality through in-vivo preclinical investigation.

## Figures and Tables

**Table 1:** In-vitro ^a^ data of MTT-cell proliferation assay for promising synthesized compounds on both cancerous and non-cancerous cell lines.

Compound ^b^ No.	MDA-MB-231 IC_50_ (µM) ^c^	A549 IC_50_ (µM) ^c^	MDCK IC_50_ (µM) ^c^
**3a**	29.68 ± 4.03	ND	ND
**7b**	43.77 ± 1.09	ND	28.92 ± 1.49
**7d**	17.38 ± 3.23	ND	ND
**8a**	12.69 ± 0.84	ND	73.86 ± 2.30
**8b**	12.71 ± 2.48	23.05 ± 1.45	92.75 ± 0.43
**8c**	12.83 ± 3.50	11.63 ± 2.57	87.29 ± 3.00
**8d**	31.46 ± 4.66	ND	ND
**8f**	21.64 ± 0.28	ND	ND

^a^ In-vitro data are reported as mean of three independent experiments ± SEM; ^b^ Tested compounds were those depicted % cell viability < 85%, on both cancerous cell lines, at their respective 100 µM concentrations; ^c^ IC_50_ values represent effective concentration of given small molecule being capable of inducing 50% inhibitory response of its own intrinsic maximum response following 48 h of drug exposure; ND = Not-detected.

**Table 2:** The formulation factors and responses of 23 factorial design for drug-solid lipid nanoparticles (SLNs).

**Factors and Responses**		**Level Used**	
**Factor**	**Name**	**Low (−1)**	**High (+1)**
**A: X_1_**	Type of lipid	Compritol 888 ATO (COMP)	Glyceryl Monostearate (GMS)
**B: X_2_**	Type of surfactant	Cremophor RH40	Poloxamer 188
**C: X_3_**	Surfactant conc. (% *w*/*v*)	1%	1.5%
**Response**	**Name**	**Goal**	
**Y_1_**	EE (%)	**Maximize**	
**Y_2_**	PS (nm)	**Minimize**	
**Y_3_**	ZP (mV)	**Maximize**	

**Table 3:** The eight designed drug-SLNs according to 2^3^-factorial design.

SNP Formulation No.	X_1_	X_2_	X_3_
	A: Lipid Type	B: Surfactant Lipid	C: Surfactant Conc. (% *w*/*v*)
**1**	COMP	Cremophor RH40	1.5
**2**	COMP	Cremophor RH40	1
**3**	COMP	Poloxamer 188	1
**4**	GMS	Cremophor RH40	1
**5**	COMP	Poloxamer 188	1.5
**6**	GMS	Cremophor RH40	1.5
**7**	GMS	Poloxamer 188	1
**8**	GMS	Poloxamer 188	1.5

**Table 4:** The measured responses of prepared drug-SLNs according to 2^3^ factorial design.

SNP Formulation No.	(Y1) EE%	(Y2) PS (nm)	(Y3) ZP (mV)	Polydispersity Index (PDI)
F1	37.1 ± 2.45	140.9 ± 3.2	−12.3 ± 0.77	0.352 ± 0.03
F2	53.4 ± 1.65	135.1 ± 1.0	−13.6 ± 0.32	0.282 ± 0.01
F3	95.3 ± 1.34	329.4 ± 6.1	−21.7 ± 0.15	0.563 ± 0.03
F4	82.8 ± 2.55	189.8 ± 3.3	−36.1 ± 1.90	0.432 ± 0.04
F5	86.8 ± 3.32	181.5 ± 2.1	−16.4 ± 1.10	0.442 ± 0.02
F6	67.7 ± 1.52	100.4 ± 0.4	−39.4 ± 0.095	0.433 ± 0.01
F7	94.6 ± 2.67	537.3 ± 10.4	−37.5 ± 0.58	0.582 ± 0.04
F8	92.8 ± 2.38	226.1 ±10.5	−29.3 ± 1.17	0.639 ± 0.12

**Table 5:** Specific forward/reverse primer sequence of Casp-3, -8, -9, BAX and Bcl2.

Gene	Forward	Reverse
*BAX*	5′-AGTGGCAGCTGACATGTTTT-3′	5′-GGAGGAAGTCCAATGTCCAG-3′
*Casp-3*	5′-GGCCCTGAAATACGAAGTC-3′	5′-GGCAGTAGTCGACTCTGAAG-3′
*Casp-8*	5′-GCCTCCCTCAAGTTCCT-3′	5′-CCTGGAGTCTCTGGAATAACA-3′
*Casp-9*	5′-CGAACTAACAGGCAAGCAG-3′	5′-ACCTCACCAAATCCTCCAGAAC-3′
*BcL-2*	5′-CCTGTGGATGACTGAGTACC-3′	5′-GAGACAGCCAGGAGAAATCA-3′

## Data Availability

The data presented in this study are available within the article or on request from the corresponding author.

## Supplementary Materials

The following are available online at https://www.mdpi.com/1424-8247/14/2/113/s1, Figure S1: Standard calibration curve of **8b** using HPLC-UV analysis, Figure S2: NMR charts of all new compounds, Table S1: The Data of Ligand-Docking Studies on Bcl-2 anti-apoptotic protein target.

## References

[1] Portt L., Norman G., Clapp C., Greenwood M., Greenwood M.T. (2011). Anti-apoptosis and cell survival: A review. Biochim. Biophys. Acta.

[2] Okada H., Mak T.W. (2004). Pathways of apoptotic and non-apoptotic death in tumour cells. Nat. Rev. Cancer.

[3] Hengartner M.O. (2000). The biochemistry of apoptosis. Nature.

[4] Cory S., Adams J.M. (2002). The Bcl2 family: Regulators of the cellular life-or-death switch. Nat. Rev. Cancer.

[5] Youle R.J., Strasser A. (2008). The BCL-2 protein family: Opposing activities that mediate cell death. Nat. Rev. Mol. Cell Biol..

[6] Garner T.P., Lopez A., Reyna D.E., Spitz A.Z., Gavathiotis E. (2017). Progress in targeting the BCL-2 family of proteins. Curr. Opin. Chem. Biol..

[7] Thomas S., Quinn B.A., Das S.K., Dash R., Emdad L., Dasgupta S., Wang X.Y., Dent P., Reed J.C., Pellecchia M. (2013). Targeting the Bcl-2 family for cancer therapy. Expert Opin. Ther. Targets.

[8] Yip K.W., Reed J.C. (2008). Bcl-2 family proteins and cancer. Oncogene.

[9] Birkinshaw R.W., Gong J.-N., Luo C.S., Lio D., White C.A., Anderson M.A., Blombery P., Lessene G., Majewski I.J., Thijssen R. (2019). Structures of BCL-2 in complex with venetoclax reveal the molecular basis of resistance mutations. Nat. Commun..

[10] Besbes S., Mirshahi M., Pocard M., Billard C. (2015). New dimension in therapeutic targeting of BCL-2 family proteins. Oncotarget.

[11] Souers A.J., Leverson J.D., Boghaert E.R., Ackler S.L., Catron N.D., Chen J., Dayton B.D., Ding H., Enschede S.H., Fairbrother W.J. (2013). ABT-199, a potent and selective BCL-2 inhibitor, achieves antitumor activity while sparing platelets. Nat. Med..

[12] Iyer D., Vartak S.V., Mishra A., Goldsmith G., Kumar S., Srivastava M., Hegde M., Gopalakrishnan V., Glenn M., Velusamy M. (2016). Identification of a novel BCL2-specific inhibitor that binds predominantly to the BH1 domain. Febs J..

[13] Vartak S.V., Hegde M., Iyer D., Gaikwad S., Gopalakrishnan V., Srivastava M., Karki S.S., Choudhary B., Ray P., Santhoshkumar T.R. (2016). A novel inhibitor of BCL2, Disarib abrogates tumor growth while sparing platelets, by activating intrinsic pathway of apoptosis. Biochem. Pharmacol..

[14] Kang M.H., Reynolds C.P. (2009). Bcl-2 inhibitors: Targeting mitochondrial apoptotic pathways in cancer therapy. Clin. Cancer Res. Off. J. Am. Assoc. Cancer Res..

[15] Paik P.K., Rudin C.M., Pietanza M.C., Brown A., Rizvi N.A., Takebe N., Travis W., James L., Ginsberg M.S., Juergens R. (2011). A phase II study of obatoclax mesylate, a Bcl-2 antagonist, plus topotecan in relapsed small cell lung cancer. Lung Cancer.

[16] Li J., Viallet J., Haura E.B. (2008). A small molecule pan-Bcl-2 family inhibitor, GX15–070, induces apoptosis and enhances cisplatin-induced apoptosis in non-small cell lung cancer cells. Cancer Chemother. Pharmacol..

[17] Zhai D., Jin C., Satterthwait A.C., Reed J.C. (2006). Comparison of chemical inhibitors of antiapoptotic Bcl-2-family proteins. Cell Death Differ..

[18] Tse C., Shoemaker A.R., Adickes J., Anderson M.G., Chen J., Jin S., Johnson E.F., Marsh K.C., Mitten M.J., Nimmer P. (2008). ABT-263: A potent and orally bioavailable Bcl-2 family inhibitor. Cancer Res..

[19] Koehler B.C., Jassowicz A., Scherr A.L., Lorenz S., Radhakrishnan P., Kautz N., Elssner C., Weiss J., Jaeger D., Schneider M. (2015). Pan-Bcl-2 inhibitor Obatoclax is a potent late stage autophagy inhibitor in colorectal cancer cells independent of canonical autophagy signaling. BMC Cancer.

[20] Schwartz-Roberts J.L., Shajahan A.N., Cook K.L., Wärri A., Abu-Asab M., Clarke R. (2013). GX15–070 (obatoclax) induces apoptosis and inhibits cathepsin D- and L-mediated autophagosomal lysis in antiestrogen-resistant breast cancer cells. Mol. Cancer Ther..

[21] Stamelos V.A., Fisher N., Bamrah H., Voisey C., Price J.C., Farrell W.E., Redman C.W., Richardson A. (2016). The BH3 mimetic obatoclax accumulates in lysosomes and causes their alkalinization. PLoS ONE.

[22] Basit F., Cristofanon S., Fulda S. (2013). Obatoclax (GX15–070) triggers necroptosis by promoting the assembly of the necrosome on autophagosomal membranes. Cell Death Differ..

[23] Zhong D., Gu C., Shi L., Xun T., Li X., Liu S., Yu L. (2014). Obatoclax induces G1/G0-phase arrest via p38/p21(waf1/Cip1) signaling pathway in human esophageal cancer cells. J. Cell Biochem..

[24] Zacarías-Lara O.J., Correa-Basurto J., Bello M. (2016). Exploring the conformational and binding properties of unphosphorylated/phosphorylated monomeric and trimeric Bcl-2 through docking and molecular dynamics simulations. Biopolymers.

[25] Cornils B., Herrmann W., Cornils B., Zanthoff H., Xu J.-H. (2020). Philips Reaction. Catalysis from A to Z.

[26] Achar K.C., Hosamani K.M., Seetharamareddy H.R. (2010). In-vivo analgesic and anti-inflammatory activities of newly synthesized benzimidazole derivatives. Eur. J. Med. Chem..

[27] Colella M., Degennaro L., Luisi R. (2020). Continuous flow synthesis of heterocycles: A recent update on the flow synthesis of indoles. Molecules.

[28] Kumar S., Kumar P., Sati N. (2012). Synthesis and biological evaluation of Schiff bases and azetidinones of 1-naphthol. J. Pharm. Bioallied Sci..

[29] Kishk S.M., McLean K.J., Sood S., Smith D., Evans J.W.D., Helal M.A., Gomaa M.S., Salama I., Mostafa S.M., de Carvalho L.P.S. (2019). Design and synthesis of imidazole and triazole pyrazoles as mycobacterium tuberculosis CYP121A1 inhibitors. ChemistryOpen.

[30] Berridge M.V., Herst P.M., Tan A.S. (2005). Tetrazolium dyes as tools in cell biology: New insights into their cellular reduction. Biotechnol. Ann. Rev..

[31] Kalluri R., Weinberg R.A. (2009). The basics of epithelial-mesenchymal transition. J. Clin. Investig..

[32] Van den Eijnde S.M., Luijsterburg A.J., Boshart L., De Zeeuw C.I., van Dierendonck J.H., Reutelingsperger C.P., Vermeij-Keers C. (1997). In situ detection of apoptosis during embryogenesis with annexin V: From whole mount to ultrastructure. Cytometry.

[33] Darzynkiewicz Z. (2011). Critical aspects in analysis of cellular DNA content. Curr. Protoc. Cytom..

[34] Darzynkiewicz Z., Juan G. (2001). DNA content measurement for DNA ploidy and cell cycle analysis. Curr. Protoc. Cytom..

[35] Goldsmith K.C., Gross M., Peirce S., Luyindula D., Liu X., Vu A., Sliozberg M., Guo R., Zhao H., Reynolds C.P. (2012). Mitochondrial Bcl-2 family dynamics define therapy response and resistance in neuroblastoma. Cancer Res..

[36] Kumar S. (1999). Regulation of caspase activation in apoptosis: Implications in pathogenesis and treatment of disease. Clin. Exp. Pharmacol. Physiol..

[37] Ouyang L., Shi Z., Zhao S., Wang F.T., Zhou T.T., Liu B., Bao J.K. (2012). Programmed cell death pathways in cancer: A review of apoptosis, autophagy and programmed necrosis. Cell Prolif..

[38] Ferreira K.S., Kreutz C., Macnelly S., Neubert K., Haber A., Bogyo M., Timmer J., Borner C. (2012). Caspase-3 feeds back on caspase-8, Bid and XIAP in type I Fas signaling in primary mouse hepatocytes. Apoptosis.

[39] Mariani M., Karki R., Spennato M., Pandya D., He S., Andreoli M., Fiedler P., Ferlini C. (2015). Class III β-tubulin in normal and cancer tissues. Gene.

[40] Sharifi S., Barar J., Hejazi M.S., Samadi N. (2014). Roles of the Bcl-2/Bax ratio, caspase-8 and 9 in resistance of breast cancer cells to paclitaxel. Asian Pac. J. Cancer Prev..

[41] Murray J.B., Davidson J., Chen I., Davis B., Dokurno P., Graham C.J., Harris R., Jordan A., Matassova N., Pedder C. (2019). Establishing drug discovery and identification of hit series for the anti-apoptotic proteins, Bcl-2 and Mcl-1. ACS Omega.

[42] Kontoyianni M., McClellan L.M., Sokol G.S. (2004). Evaluation of docking performance:  Comparative data on docking algorithms. J. Med. Chem..

[43] Severino P., Andreani T., Macedo A.S., Fangueiro J.F., Santana M.H.A., Silva A.M., Souto E.B. (2012). Current state-of-art and new trends on lipid nanoparticles (SLN and NLC) for oral drug delivery. J. Drug Deliv..

[44] Pardeshi C., Rajput P., Belgamwar V., Tekade A., Patil G., Chaudhary K., Sonje A. (2012). Solid lipid based nanocarriers: An overview. Acta Pharm..

[45] Mukherjee S., Ray S., Thakur R.S. (2009). Solid lipid nanoparticles: A modern formulation approach in drug delivery system. Indian J. Pharm. Sci..

[46] Müller R.H., Mäder K., Gohla S. (2000). Solid lipid nanoparticles (SLN) for controlled drug delivery—A review of the state of the art. Eur. J. Pharm. Biopharm..

[47] Ekambaram P., Abdul H.S.A. (2011). Formulation and evaluation of solid lipid nanoparticles of ramipril. J. Young Pharm. JYP.

[48] Qushawy M., Prabahar K., Abd-Alhaseeb M., Swidan S., Nasr A. (2019). Preparation and evaluation of carbamazepine solid lipid nanoparticle for alleviating seizure activity in pentylenetetrazole-kindled mice. Molecules.

[49] Fernandes R.S., Silva J.O., Seabra H.A., Oliveira M.S., Carregal V.M., Vilela J.M.C., Andrade M.S., Townsend D.M., Colletti P.M., Leite E.A. (2018). α-Tocopherol succinate loaded nano-structed lipid carriers improves antitumor activity of doxorubicin in breast cancer models in vivo. Biomed. Pharmacother..

[50] Joseph E., Reddi S., Rinwa V., Balwani G., Saha R. (2017). Design and in vivo evaluation of solid lipid nanoparticulate systems of Olanzapine for acute phase schizophrenia treatment: Investigations on antipsychotic potential and adverse effects. Eur. J. Pharm. Sci..

[51] Sznitowska M., Wolska E., Baranska H., Cal K., Pietkiewicz J. (2017). The effect of a lipid composition and a surfactant on the characteristics of the solid lipid microspheres and nanospheres (SLM and SLN). Eur. J. Pharm. Biopharm..

[52] Priyanka K., Sathali A.A. (2012). Preparation and evaluation of montelukast sodium loaded solid lipid nanoparticles. J. Young Pharm..

[53] El-Housiny S., Shams Eldeen M.A., El-Attar Y.A., Salem H.A., Attia D., Bendas E.R., El-Nabarawi M.A. (2018). Fluconazole-loaded solid lipid nanoparticles topical gel for treatment of pityriasis versicolor: Formulation and clinical study. Drug Deliv..

[54] Zielińska A., Ferreira N.R., Durazzo A., Lucarini M., Cicero N., Mamouni S.E., Silva A.M., Nowak I., Santini A., Souto E.B. (2019). Development and optimization of alpha-pinene-loaded solid lipid nanoparticles (SLN) using experimental factorial design and dispersion analysis. Molecules.

[55] Gardouh A.R., Attia M.A., Enan E.T., Elbahaie A.M., Fouad R.A., El-Shafey M., Youssef A.M., Alomar S.Y., Ali Z.A.-E., Zaitone S.A. (2020). Synthesis and antitumor activity of doxycycline polymeric nanoparticles: Effect on tumor apoptosis in solid ehrlich carcinoma. Molecules.

[56] Hong I.K., Kim S.I., Lee S.B. (2018). Effects of HLB value on oil-in-water emulsions: Droplet size, rheological behavior, zeta-potential, and creaming index. J. Ind. Eng. Chem..

[57] Schwarz C., Mehnert W. (1999). Solid lipid nanoparticles (SLN) for controlled drug delivery. II. Drug incorporation and physicochemical characterization. J. Microencapsul..

[58] Fasehee H., Dinarvand R., Ghavamzadeh A., Esfandyari-Manesh M., Moradian H., Faghihi S., Ghaffari S.H. (2016). Delivery of disulfiram into breast cancer cells using folate-receptor-targeted PLGA-PEG nanoparticles: In vitro and in vivo investigations. J. Nanobiotechnol..

[59] Jain A., Agarwal A., Majumder S., Lariya N., Khaya A., Agrawal H., Majumdar S., Agrawal G.P. (2010). Mannosylated solid lipid nanoparticles as vectors for site-specific delivery of an anti-cancer drug. J. Control. Release.

[60] Kalepu S., Nekkanti V. (2015). Insoluble drug delivery strategies: Review of recent advances and business prospects. Acta Pharm. Sin. B.

[61] Swidan S., Ghonaim H., Samy A., Ghorab M. (2016). Efficacy and in vitro cytotoxicity of nanostructured lipid carriers for paclitaxel delivery. J. Appl. Pharm. Sci..

[62] Wang W., Zhang L., Chen T., Guo W., Bao X., Wang D., Ren B., Wang H., Li Y., Wang Y. (2017). Anticancer effects of resveratrol-loaded solid lipid nanoparticles on human breast cancer cells. Molecules.

[63] Gutierréz-Hernández A., Galván-Ciprés Y., Domínguez-Mendoza E.A., Aguirre-Vidal Y., Estrada-Soto S., Almanza-Pérez J.C., Navarrete-Vázquez G. (2019). Design, Synthesis, Antihyperglycemic studies, and docking simulations of benzimidazole-thiazolidinedione hybrids. J. Chem..

[64] Wang W., Pang J., Ha E.H., Zhou M., Li Z., Tian S., Li H., Hu Q. (2020). Development of novel NLRP3-XOD dual inhibitors for the treatment of gout. Bioorg Med Chem Lett..

[65] Basceken S., Kaya S., Balci M. (2015). Intramolecular gold-catalyzed and NaH-supported cyclization reactions of N-propargyl indole derivatives with pyrazole and pyrrole rings: Synthesis of pyrazolodiazepinoindole, pyrazolopyrazinoindole, and pyrrolopyrazinoindole. J. Org. Chem..

[66] Demurtas M., Baldisserotto A., Lampronti I., Moi D., Balboni G., Pacifico S., Vertuani S., Manfredini S., Onnis V. (2019). Indole derivatives as multifunctional drugs: Synthesis and evaluation of antioxidant, photoprotective and antiproliferative activity of indole hydrazones. Bioorganic Chem..

[67] Mirfazli S.S., Kobarfard F., Firoozpour L., Asadipour A., Esfahanizadeh M., Tabib K., Shafiee A., Foroumadi A. (2014). N-substituted indole carbohydrazide derivatives: Synthesis and evaluation of their antiplatelet aggregation activity. DARU.

[68] Boraei A.T., El Ashry S.H., Barakat A., Ghabbour H.A. (2016). Synthesis of new functionalized indoles based on ethyl indol-2-carboxylate. Molecules.

[69] Al-Ebaisat H.S., Ababne T.S., Al Shboul T.M., Jazzazi T.M. (2016). Synthesis, characterization and antifungal activity of some substituted 4-thiazolidinone derivatives. J. Pure Appl. Chem. Res..

[70] Maurya D.K., Nandakumar N., Devasagayam T.P.A. (2011). Anticancer property of gallic acid in A549, a human lung adenocarcinoma cell line, and possible mechanisms. J. Clin. Biochem. Nutr..

[71] Ranganathan S., Halagowder D., Sivasithambaram N.D. (2015). Quercetin suppresses twist to induce apoptosis in MCF-7 breast cancer cells. PLoS ONE.

[72] Koulaouzidou E.A., Papazisis K.T., Economides N.A., Beltes P., Kortsaris A.H. (2005). Antiproliferative effect of mineral trioxide aggregate, zinc oxide-eugenol cement, and glass-ionomer cement against three fibroblastic cell lines. J. Endod..

[73] Dey N.S., Mukherjee B., Maji R., Satapathy B.S. (2016). Development of linker-conjugated nanosize lipid vesicles: A strategy for cell selective treatment in breast cancer. Curr. Cancer Drug Targets.

[74] Wang W., Chen T., Xu H., Ren B., Cheng X., Qi R., Liu H., Wang Y., Yan L., Chen S. (2018). Curcumin-loaded solid lipid nanoparticles enhanced anticancer efficiency in breast cancer. Molecules.

[75] Jung B., Shim M.K., Park M.J., Jang E.H., Yoon H.Y., Kim K., Kim J.H. (2017). Hydrophobically modified polysaccharide-based on polysialic acid nanoparticles as carriers for anticancer drugs. Int. J. Pharm..

[76] Hegazy M.G.A., Imam A.M., Abdelghany B.E. (2020). Evaluation of cytotoxic and anticancer effect of Orobanche crenata methanolic extract on cancer cell lines. Tumor Biol..

[77] Tantawy M.A., Sroor F.M., Mohamed M.F., El-Naggar M.E., Saleh F.M., Hassaneen H.M., Abdelhamid I.A. (2020). Molecular docking study, cytotoxicity, cell cycle arrest and apoptotic induction of novel chalcones incorporating thiadiazolyl isoquinoline in cervical cancer. Anticancer Agents Med. Chem..

[78] Zhao Y., Xiang S., Dai X., Yang K. (2013). A simplified diphenylamine colorimetric method for growth quantification. Appl. Microbiol. Biotechnol..

[79] Preusse M., Tantawy M.A., Klawonn F., Schughart K., Pessler F. (2013). Infection- and procedure-dependent effects on pulmonary gene expression in the early phase of influenza A virus infection in mice. BMC Microbiol..

[80] Petersen H., Mostafa A., Tantawy M.A., Iqbal A.A., Hoffmann D., Tallam A., Selvakumar B., Pessler F., Beer M., Rautenschlein S. (2018). NS segment of a 1918 influenza a virus-descendent enhances replication of H1N1pdm09 and virus-induced cellular immune response in mammalian and avian systems. Front. Microbiol..

[81] El Raey M.A., El-Hagrassi A.M., Osman A.F., Darwish K.M., Emam M. (2019). Acalypha wilkesiana flowers: Phenolic profiling, cytotoxic activity of their biosynthesized silver nanoparticles and molecular docking study for its constituents as Topoisomerase-I inhibitors. Biocatal. Agric. Biotechnol..

[82] Malebari A., Ibrahim T., Salem I., Salama I., Khayyat A., Mostafa S., El-Sabbagh O., Darwish K. (2020). The anticancer activity for the bumetanide-based analogs via targeting the tumor-associated membrane bound human carbonic anhydrase-IX enzyme. Pharmaceuticals.

[83] Wadie M.A., Kishk S.M., Darwish K.M., Mostafa S.M., Elgawish M.S. (2020). Simultaneous determination of losartan and rosuvastatin in rat plasma using liquid chromatography–tandem mass spectrometric technique for application into pharmacokinetic and drug–drug interaction studies. Chromatographia.

[84] Vilar S., Cozza G., Moro S. (2008). Medicinal chemistry and the molecular operating environment (MOE): Application of QSAR and molecular docking to drug discovery. Curr. Top. Med. Chem..

[85] (2016). The PyMOL Molecular Graphics System, 2.0.6.

[86] Souto E.B., Doktorovova S., Zielinska A., Silva A.M. (2019). Key production parameters for the development of solid lipid nanoparticles by high shear homogenization. Pharm. Dev. Technol..

[87] Da Rocha Lindner G., Khalil N.M., Mainardes R.M. (2013). Resveratrol-loaded polymeric nanoparticles: Validation of an HPLC-PDA method to determine the drug entrapment and evaluation of its antioxidant activity. Sci. World J..

[88] Nasr A.M., Qushawy M.K., Elkhoudary M.M., Gawish A.Y., Elhady S.S., Swidan S.A. (2020). Quality by design for the development and analysis of enhanced in-situ forming vesicles for the improvement of the bioavailability of fexofenadine HCl in vitro and in vivo. Pharmaceutics.

[89] Aldawsari H.M., Singh S. (2020). Rapid microwave-assisted cisplatin-loaded solid lipid nanoparticles: Synthesis, characterization and anticancer study. Nanomaterials.

[90] Mona Q., Ali N. (2019). Solid lipid nanoparticles (SLNs) as nano drug delivery carriers: Preparation, characterization and application. Int. J. Appl. Pharm..

