# Supplementary material for: Design, Synthesis, Anticancer Activity, and Solid Lipid Nanoparticle Formulation of Indole- and Benzimidazole-Based Compounds as Pro-Apoptotic Agents Targeting Bcl-2 Protein
Source: Pharmaceuticals (Basel). 2021 Feb 1;14(2):113. doi: 10.3390/ph14020113 (PMC7912796; doi:10.3390/ph14020113)
Supplement: Supplementary file 1 [file pharmaceuticals-14-00113-s001.pdf]

Supplementary materials

Design, Synthesis, Anticancer Activity, and Solid Lipid Nanoparticle Formulation of Indole-Based Compounds as Novel Pro-Apoptotic Agents Targeting Bcl-2 Protein

Manar I. Nagy[1], Khaled M. Darwish[1], Safaa M. Kishk[1], Mohamed A. Tantawy[2], Ali Nasr[3,4], Mona Qushawy[4,5], Shady Swidan[6,7], Samia M. Mostafa[1], and Ismail Salama[1*]

[1] Department of Medicinal Chemistry, Faculty of Pharmacy, Suez Canal University, Ismailia 41522, Egypt; manar.nagy@pharm.suez.edu.eg (M.I.N.); khaled_darwish@pharm.suez.edu.eg (K.M.D.); safaa_keshk@pharm.suez.edu.eg (S.M.K.); samiamostafa2010@hotmail.com (S.M.M.); ismail_mohamed@pharm.suez.edu.eg (I.S.)

[2] Hormones Department, Medical Research Division, National Research Center, Dokki, Giza,12622, Egypt; mohamed_tantawy@daad-alumni.de (M.T.)

[3] Department of Pharmaceutics, Faculty of Pharmacy, Port Said University, Port Said 42526, Egypt; a.nasr@pharm.psu.edu.eg (A.N.)

[4] Department of Pharmaceutics, Faculty of Pharmacy, Sinai University, Alarish, North Sinai 45511, Egypt

[5] Department of Pharmaceutics, Faculty of Pharmacy, University of Tabuk, Tabuk 71491, Saudi Arabia; mqushawy@ut.edu.sa (M.Q.)

[6] Department of Pharmaceutics, Faculty of Pharmacy, The British University in Egypt, El-Sherouk city, Cairo 11837, Egypt; shady.swidan@bue.edu.eg (S.S.)

[7] The Center for Drug Research and Development (CDRD), Faculty of Pharmacy, The British University in Egypt, El-Sherouk city, Cairo 11837, Egypt

* Correspondence: ismail_mohamed@pharm.suez.edu.eg (I.S.); Tel.: +02-0102-225-7643

Additional Experimental Detail

1) **Table S1:** The Data of Ligand-Docking Studies on Bcl-2 anti-apoptotic protein target.

2) **Figure S1:** Standard calibration curve of 8b using HPLC-UV analysis. All data are represented as mean of three independent experiments ± SEM.

3) **Figure S2:** NMR spectra of all new compounds

Table S1: The Data of Ligand-Docking Studies on Bcl-2 anti-apoptotic protein target

Ligand name	MOE docking Scores (*S*) [a] (Kcal moL^{-1})	RMSD [b] (Å)	Ligand-target interaction description [Type; Length (Å); Angle (°); Binding Residues]
**3a**	-4.2200	1.7838	Hydrogen bonding ; 2.51 **Å** ; 148.0° ; Asp111-C=**O** side chain with benzimidazole (N**H**) ring<br>π-π stacking ; 3.85 **Å** ; Tyr108 side chain with benzimidazole ring
**7b**	-4.7696	1.5547	Hydrogen bonding ; 2.98 **Å** ; 112.5° ; Arg146-=N**H**H side chain with 4-Me**O**
**7d**	-4.5808	1.7670	Hydrogen bonding ; 2.10 **Å** ; 149.9° ; Arg146-=N**H**H side chain with pyridine (**N**) ring<br>π-Hydrogen contact ; 4.15 **Å** ; Leu137 side chain with pyridine ring<br>π-Hydrogen contact ; 4.17 **Å** ; Leu137 side chain with pyridine ring
**8a**	-4.7696	1.2117	Hydrogen bonding ; 2.10 **Å** ; 158.7° ; Arg146–=N**H**H side chain with NHC=**O** linker
**8b**	-4.8585	1.6829	Hydrogen bonding ; 2.15 **Å** ; 175.5° ; Arg146–=N**H**H side chain with NHC=**O** linker
**8c**	-6.2460	1.7374	Hydrogen bonding ; 2.66 **Å** ; 129.0° ; Arg146–=N**H**H side chain with indole (**N**H) ring<br>π-Hydrogen interaction ; 3.33 **Å** ; Arg146-N**H** side chain with indole ring
**8d**	-6.1846	1.3960	Hydrogen bonding ; 2.08 **Å** ; 156.0° ; Arg146–=N**H**H side chain with NHC=**O** linker<br>Hydrogen bonding ; 2.53 **Å** ; 142.5° ; Arg146-N**H** side chain with NHC=**O** linker
**8f**	-5.7070	2.6217	Hydrogen bonding ; 2.30 **Å** ; 160.1° ; Arg146–=N**H**H side chain with NHC=**O** linker<br>π-Hydrogen interaction ; 3.10 **Å** ; Arg146-N**H** side chain with indole ring

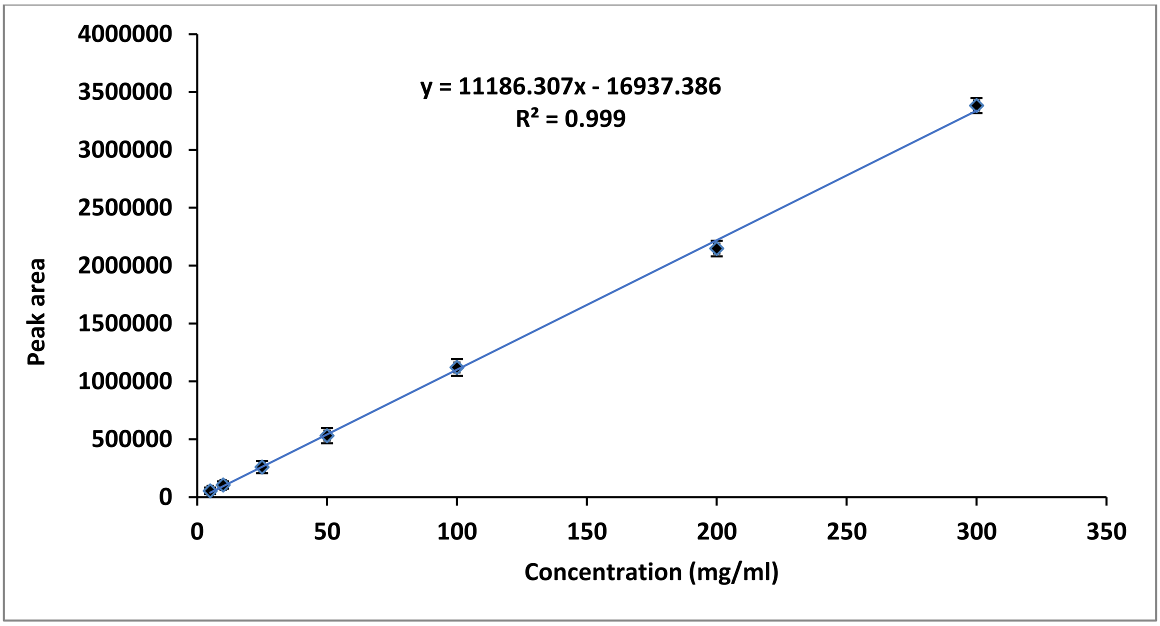

Figure S1: Standard calibration curve of 8b using HPLC-UV analysis. All data are represented as mean of three independent experiments ± SEM.

Figure S2: NMR spectra of all new compounds

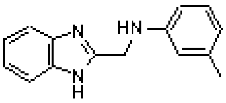

3a

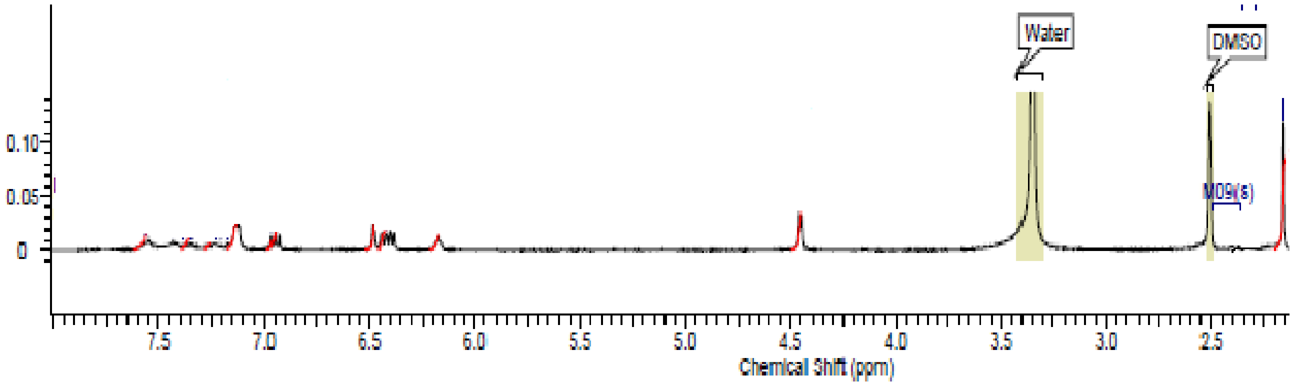

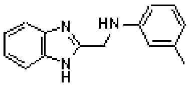

3a

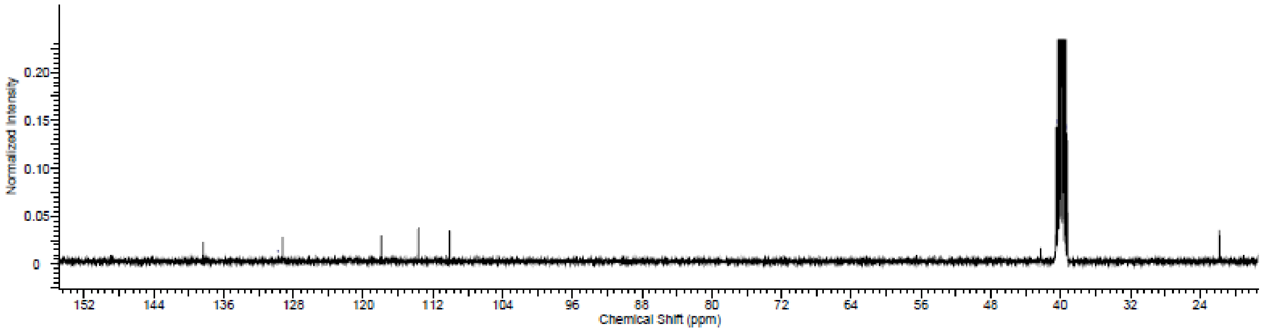

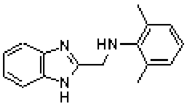

3b

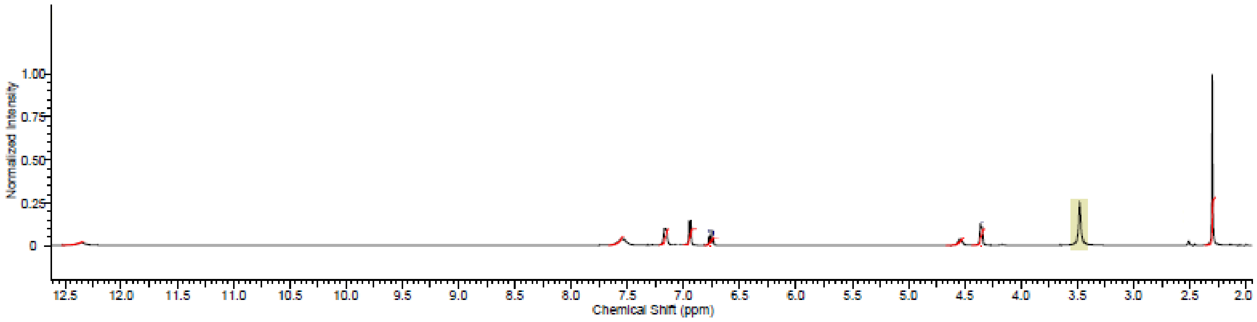

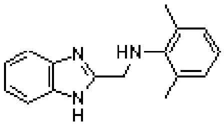

3b

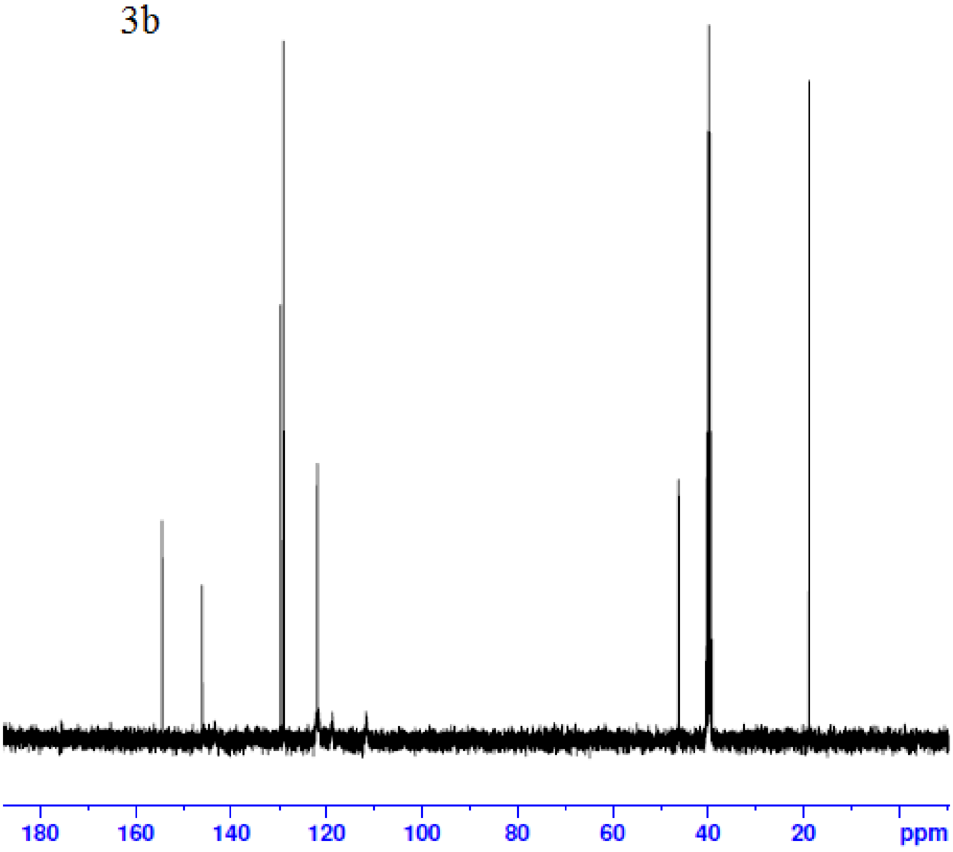


```
Current Data Parameters
NAME              manar-3
EXPNO                   2
PROCNO                  1

F2 - Acquisition Parameters
Date_            20180812
Time                10.05
INSTRUM             spect
PROBHD   5 mm PABBO BB/
PULPROG           zgpg30
TD                  65536
SOLVENT              DMSO
NS                    245
DS                      4
SWH             24038.461 Hz
FIDRES           0.366798 Hz
AQ              1.3631488 sec
RG                 205.37
DW                 20.800 usec
DE                   6.50 usec
TE                  299.0 K
D1             2.00000000 sec
D11            0.03000000 sec
TD0                     1

======== CHANNEL f1 ========
SFO1         100.6278588 MHz
NUC1                  13C
P1                  10.00 usec
PLW1          47.00000000 W

======== CHANNEL f2 ========
SFO2         400.1516006 MHz
NUC2                   1H
CPDPRG[2          waltz16
PCPD2               90.00 usec
PLW2          18.00000000 W
PLW12          0.34722000 W
PLW13          0.28125000 W

F2 - Processing parameters
SI                  32768
SF           100.6177975 MHz
WDW                    EM
SSB          0
LB                   1.00 Hz
GB           0
PC                   1.40
```

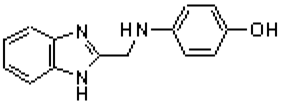

3d

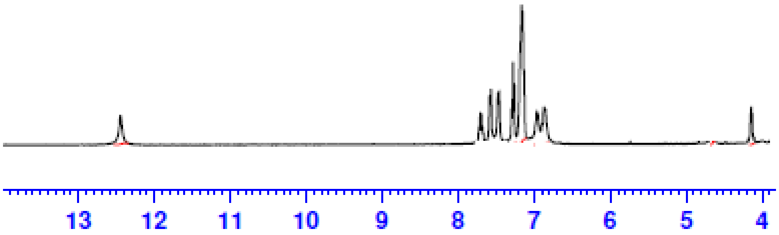


```
Current Data Parameters
NAME          manar-nagy-5
EXPNO                    1
PROCNO                   1

F2 - Acquisition Parameters
Date_             20180812
Time                 12.55
INSTRUM              spect
PROBHD     5 mm PABBO BB/
PULPROG               zg30
TD                   65536
SOLVENT               DMSO
NS                      64
DS                       2
SWH               8012.820 Hz
FIDRES            0.122266 Hz
AQ               4.0894465 sec
RG                  205.37
DW                  62.400 usec
DE                    6.50 usec
TE                   299.0 K
D1              1.00000000 sec
TD0                      1

======== CHANNEL f1 ========
SFO1           400.1524711 MHz
NUC1                    1H
P1                   12.00 usec
PLW1           18.00000000 W

F2 - Processing parameters
SI                   65536
SF             400.1500000 MHz
WDW                     EM
SSB      0
LB                    0.30 Hz
GB       0
PC                    1.00
```

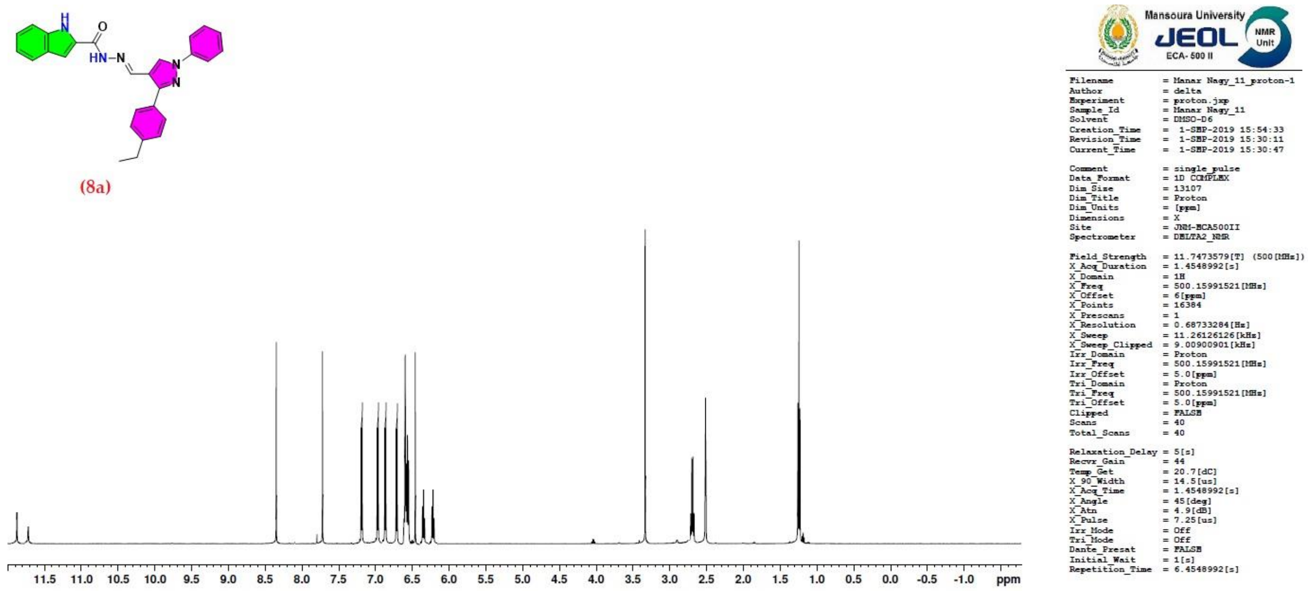
(8a)
Mansoura University
JEOL
ECA- 500 II
NMR Unit
Filename = Manar Nagy_11_proton-1
Author = delta
Experiment = proton.jxp
Sample_Id = Manar Nagy_11
Solvent = DMSO-D6
Creation_Time = 1-SEP-2019 15:54:33
Revision_Time = 1-SEP-2019 15:30:11
Current_Time = 1-SEP-2019 15:30:47
Comment = single_pulse
Data_Format = 1D COMPLEX
Dim_Size = 13107
Dim_Title = Proton
Dim_Units = [ppm]
Dimensions = X
Site = JNM-ECA500II
Spectrometer = DELTA2_NMR
Field_Strength = 11.7473579[T] (500[MHz])
X_Acq_Duration = 1.4548992[s]
X_Domain = 1H
X_Freq = 500.15991521[MHz]
X_Offset = 6[ppm]
X_Points = 16384
X_Prescans = 1
X_Resolution = 0.68733284[Hz]
X_Sweep = 11.26126126[kHz]
X_Sweep_Clipped = 9.00900901[kHz]
Irr_Domain = Proton
Irr_Freq = 500.15991521[MHz]
Irr_Offset = 5.0[ppm]
Tri_Domain = Proton
Tri_Freq = 500.15991521[MHz]
Tri_Offset = 5.0[ppm]
Clipped = FALSE
Scans = 40
Total_Scans = 40
Relaxation_Delay = 5[s]
Recvr_Gain = 44
Temp_Get = 20.7[dC]
X_90_Width = 14.5[us]
X_Acq_Time = 1.4548992[s]
X_Angle = 45[deg]
X_Atn = 4.9[dB]
X_Pulse = 7.25[us]
Irr_Mode = Off
Tri_Mode = Off
Dante_Presat = FALSE
Initial_Wait = 1[s]
Repetition_Time = 6.4548992[s]
11.5 11.0 10.5 10.0 9.5 9.0 8.5 8.0 7.5 7.0 6.5 6.0 5.5 5.0 4.5 4.0 3.5 3.0 2.5 2.0 1.5 1.0 0.5 0.0 -0.5 -1.0 ppm

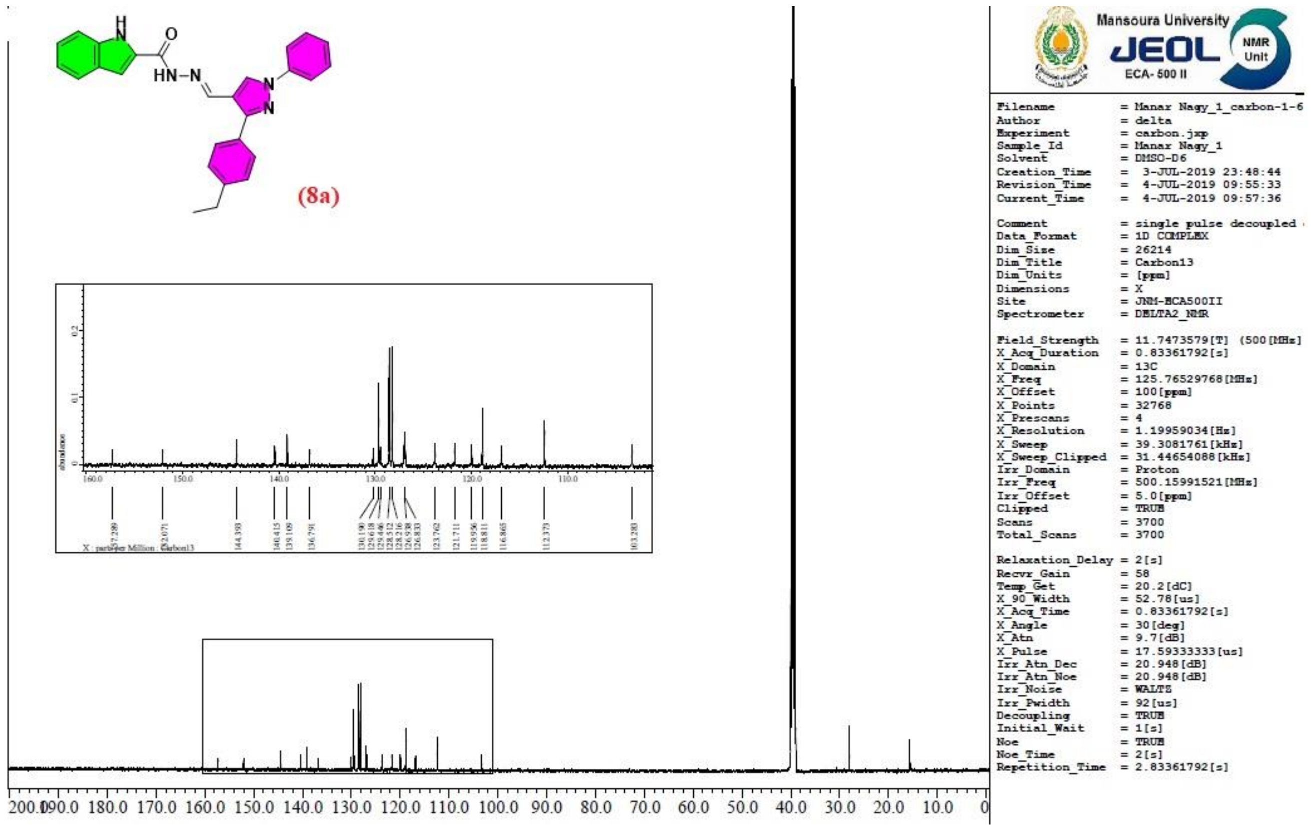
(8a)
Mansoura University
JEOL
ECA- 500 II
NMR Unit
Filename = Manar Nagy_1_carbon-1-6
Author = delta
Experiment = carbon.jxp
Sample_Id = Manar Nagy_1
Solvent = DMSO-D6
Creation_Time = 3-JUL-2019 23:48:44
Revision_Time = 4-JUL-2019 09:55:33
Current_Time = 4-JUL-2019 09:57:36
Comment = single pulse decoupled
Data_Format = 1D COMPLEX
Dim_Size = 26214
Dim_Title = Carbon13
Dim_Units = [ppm]
Dimensions = X
Site = JNM-ECA500II
Spectrometer = DELTA2_NMR
Field_Strength = 11.7473579[T] (500[MHz]
X_Acq_Duration = 0.83361792[s]
X_Domain = 13C
X_Freq = 125.76529768[MHz]
X_Offset = 100[ppm]
X_Points = 32768
X_Prescans = 4
X_Resolution = 1.19959034[Hz]
X_Sweep = 39.3081761[kHz]
X_Sweep_Clipped = 31.44654088[kHz]
Irr_Domain = Proton
Irr_Freq = 500.15991521[MHz]
Irr_Offset = 5.0[ppm]
Clipped = TRUE
Scans = 3700
Total_Scans = 3700
Relaxation_Delay = 2[s]
Recvr_Gain = 58
Temp_Get = 20.2[dC]
X_90_Width = 52.78[us]
X_Acq_Time = 0.83361792[s]
X_Angle = 30[deg]
X_Atn = 9.7[dB]
X_Pulse = 17.59333333[us]
Irr_Atn_Dec = 20.948[dB]
Irr_Atn_Noe = 20.948[dB]
Irr_Noise = WALTZ
Irr_Pwidth = 92[us]
Decoupling = TRUE
Initial_Wait = 1[s]
Noe = TRUE
Noe_Time = 2[s]
Repetition_Time = 2.83361792[s]
abundance
0 0.1 0.2
160.0 150.0 140.0 130.0 120.0 110.0
144.393
140.415
139.109
136.791
130.190
129.618
129.486
128.512
128.216
126.938
126.833
123.762
121.711
119.956
118.811
116.865
112.373
103.280
X : parts per Million : Carbon13
200.0 190.0 180.0 170.0 160.0 150.0 140.0 130.0 120.0 110.0 100.0 90.0 80.0 70.0 60.0 50.0 40.0 30.0 20.0 10.0 0

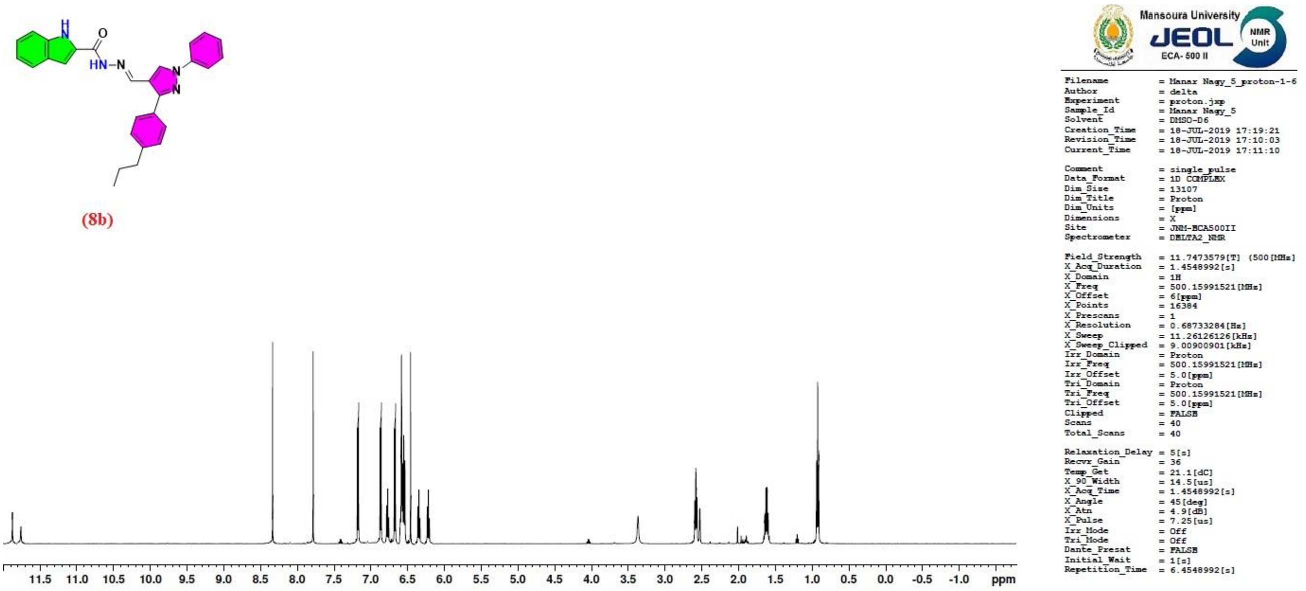

NH
O
HN–N
N
N
(8b)
Mansoura University
JEOL
ECA- 500 II
NMR Unit
Filename = Manar Nagy_5_proton-1-6
Author = delta
Experiment = proton.jxp
Sample_Id = Manar Nagy_5
Solvent = DMSO-D6
Creation_Time = 18-JUL-2019 17:19:21
Revision_Time = 18-JUL-2019 17:10:03
Current_Time = 18-JUL-2019 17:11:10
Comment = single_pulse
Data_Format = 1D COMPLEX
Dim_Size = 13107
Dim_Title = Proton
Dim_Units = [ppm]
Dimensions = X
Site = JNM-ECA500II
Spectrometer = DELTA2_NMR
Field_Strength = 11.7473579[T] (500[MHz]
X_Acq_Duration = 1.4548992[s]
X_Domain = 1H
X_Freq = 500.15991521[MHz]
X_Offset = 6[ppm]
X_Points = 16384
X_Prescans = 1
X_Resolution = 0.68733284[Hz]
X_Sweep = 11.26126126[kHz]
X_Sweep_Clipped = 9.00900901[kHz]
Irr_Domain = Proton
Irr_Freq = 500.15991521[MHz]
Irr_Offset = 5.0[ppm]
Tri_Domain = Proton
Tri_Freq = 500.15991521[MHz]
Tri_Offset = 5.0[ppm]
Clipped = FALSE
Scans = 40
Total_Scans = 40
Relaxation_Delay = 5[s]
Recvr_Gain = 36
Temp_Get = 21.1[dC]
X_90_Width = 14.5[us]
X_Acq_Time = 1.4548992[s]
X_Angle = 45[deg]
X_Atn = 4.9[dB]
X_Pulse = 7.25[us]
Irr_Mode = Off
Tri_Mode = Off
Dante_Presat = FALSE
Initial_Wait = 1[s]
Repetition_Time = 6.4548992[s]
11.5 11.0 10.5 10.0 9.5 9.0 8.5 8.0 7.5 7.0 6.5 6.0 5.5 5.0 4.5 4.0 3.5 3.0 2.5 2.0 1.5 1.0 0.5 0.0 -0.5 -1.0 ppm

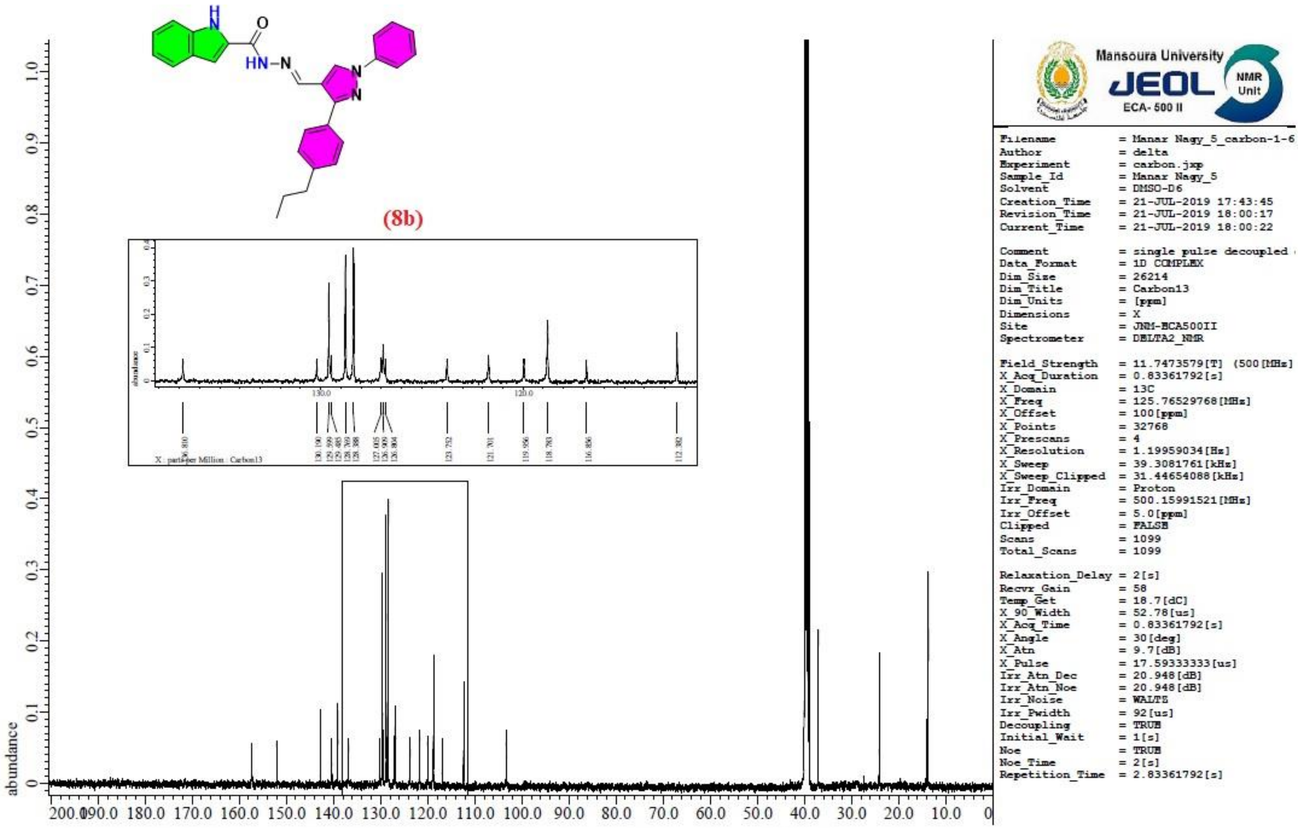

NH
O
HN–N
N
N
(8b)
36.810
130.190
129.599
129.485
128.769
128.388
127.005
126.909
126.804
123.752
121.701
119.956
118.783
116.856
112.382
130.0
120.0
abundance
X : parts per Million : Carbon13
Mansoura University
JEOL
ECA- 500 II
NMR Unit
Filename = Manar Nagy_5_carbon-1-6
Author = delta
Experiment = carbon.jxp
Sample_Id = Manar Nagy_5
Solvent = DMSO-D6
Creation_Time = 21-JUL-2019 17:43:45
Revision_Time = 21-JUL-2019 18:00:17
Current_Time = 21-JUL-2019 18:00:22
Comment = single pulse decoupled
Data_Format = 1D COMPLEX
Dim_Size = 26214
Dim_Title = Carbon13
Dim_Units = [ppm]
Dimensions = X
Site = JNM-ECA500II
Spectrometer = DELTA2_NMR
Field_Strength = 11.7473579[T] (500[MHz]
X_Acq_Duration = 0.83361792[s]
X_Domain = 13C
X_Freq = 125.76529768[MHz]
X_Offset = 100[ppm]
X_Points = 32768
X_Prescans = 4
X_Resolution = 1.19959034[Hz]
X_Sweep = 39.3081761[kHz]
X_Sweep_Clipped = 31.44654088[kHz]
Irr_Domain = Proton
Irr_Freq = 500.15991521[MHz]
Irr_Offset = 5.0[ppm]
Clipped = FALSE
Scans = 1099
Total_Scans = 1099
Relaxation_Delay = 2[s]
Recvr_Gain = 58
Temp_Get = 18.7[dC]
X_90_Width = 52.78[us]
X_Acq_Time = 0.83361792[s]
X_Angle = 30[deg]
X_Atn = 9.7[dB]
X_Pulse = 17.59333333[us]
Irr_Atn_Dec = 20.948[dB]
Irr_Atn_Noe = 20.948[dB]
Irr_Noise = WALTZ
Irr_Pwidth = 92[us]
Decoupling = TRUE
Initial_Wait = 1[s]
Noe = TRUE
Noe_Time = 2[s]
Repetition_Time = 2.83361792[s]
abundance
0 0.1 0.2 0.3 0.4 0.5 0.6 0.7 0.8 0.9 1.0
200.0 190.0 180.0 170.0 160.0 150.0 140.0 130.0 120.0 110.0 100.0 90.0 80.0 70.0 60.0 50.0 40.0 30.0 20.0 10.0 0

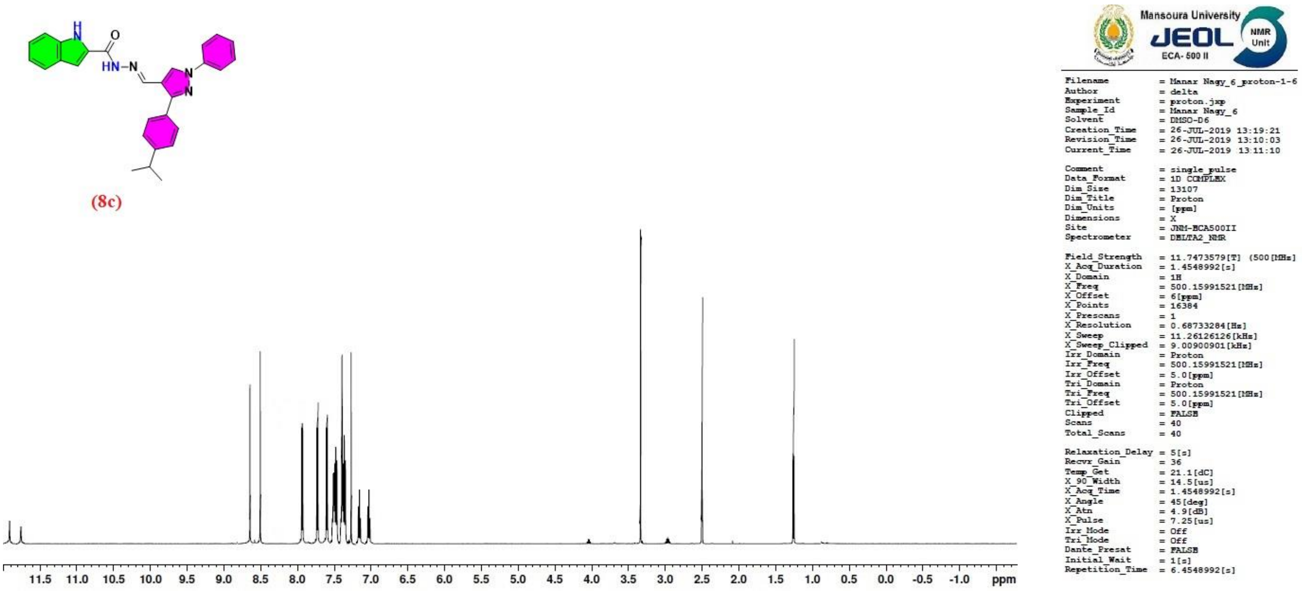

HN–N
O
NH
N
N
(8c)
Mansoura University
JEOL
NMR Unit
ECA- 500 II
Filename = Manar Nagy_6_proton-1-6
Author = delta
Experiment = proton.jxp
Sample_Id = Manar Nagy_6
Solvent = DMSO-D6
Creation_Time = 26-JUL-2019 13:19:21
Revision_Time = 26-JUL-2019 13:10:03
Current_Time = 26-JUL-2019 13:11:10
Comment = single_pulse
Data_Format = 1D COMPLEX
Dim_Size = 13107
Dim_Title = Proton
Dim_Units = [ppm]
Dimensions = X
Site = JNM-ECA500II
Spectrometer = DELTA2_NMR
Field_Strength = 11.7473579[T] (500[MHz]
X_Acq_Duration = 1.4548992[s]
X_Domain = 1H
X_Freq = 500.15991521[MHz]
X_Offset = 6[ppm]
X_Points = 16384
X_Prescans = 1
X_Resolution = 0.68733284[Hz]
X_Sweep = 11.26126126[kHz]
X_Sweep_Clipped = 9.00900901[kHz]
Irr_Domain = Proton
Irr_Freq = 500.15991521[MHz]
Irr_Offset = 5.0[ppm]
Tri_Domain = Proton
Tri_Freq = 500.15991521[MHz]
Tri_Offset = 5.0[ppm]
Clipped = FALSE
Scans = 40
Total_Scans = 40
Relaxation_Delay = 5[s]
Recvr_Gain = 36
Temp_Get = 21.1[dC]
X_90_Width = 14.5[us]
X_Acq_Time = 1.4548992[s]
X_Angle = 45[deg]
X_Atn = 4.9[dB]
X_Pulse = 7.25[us]
Irr_Mode = Off
Tri_Mode = Off
Dante_Presat = FALSE
Initial_Wait = 1[s]
Repetition_Time = 6.4548992[s]
11.5 11.0 10.5 10.0 9.5 9.0 8.5 8.0 7.5 7.0 6.5 6.0 5.5 5.0 4.5 4.0 3.5 3.0 2.5 2.0 1.5 1.0 0.5 0.0 -0.5 -1.0 ppm

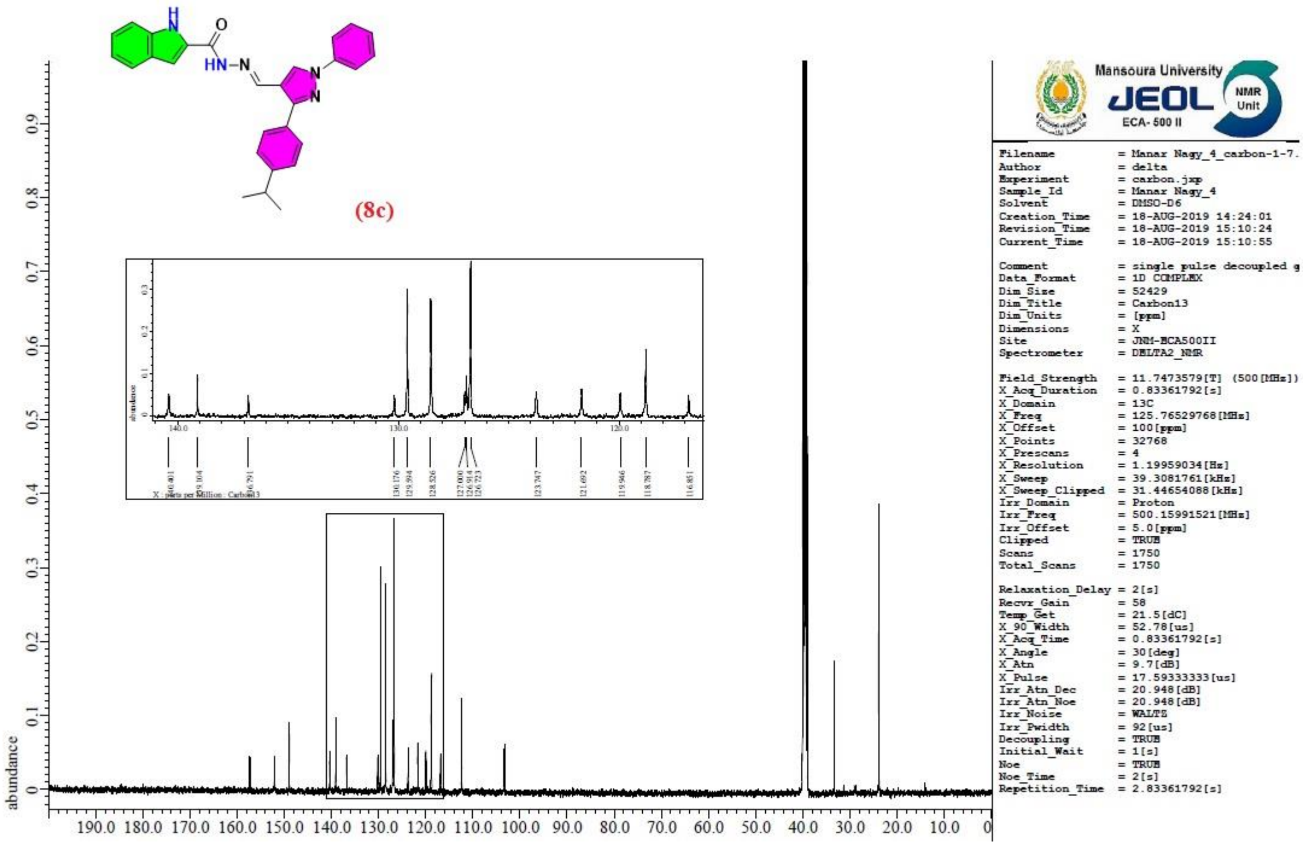

HN–N
O
NH
N
N
(8c)
abundance
0 0.1 0.2 0.3 0.4 0.5 0.6 0.7 0.8 0.9
140.0 130.0 120.0
140.401
130.176
129.994
128.526
127.000
126.914
126.723
123.747
121.692
119.946
118.787
116.851
X : parts per Million : Carbon13
190.0 180.0 170.0 160.0 150.0 140.0 130.0 120.0 110.0 100.0 90.0 80.0 70.0 60.0 50.0 40.0 30.0 20.0 10.0 0
Mansoura University
JEOL
NMR Unit
ECA- 500 II
Filename = Manar Nagy_4_carbon-1-7.
Author = delta
Experiment = carbon.jxp
Sample_Id = Manar Nagy_4
Solvent = DMSO-D6
Creation_Time = 18-AUG-2019 14:24:01
Revision_Time = 18-AUG-2019 15:10:24
Current_Time = 18-AUG-2019 15:10:55
Comment = single pulse decoupled g
Data_Format = 1D COMPLEX
Dim_Size = 52429
Dim_Title = Carbon13
Dim_Units = [ppm]
Dimensions = X
Site = JNM-ECA500II
Spectrometer = DELTA2_NMR
Field_Strength = 11.7473579[T] (500[MHz])
X_Acq_Duration = 0.83361792[s]
X_Domain = 13C
X_Freq = 125.76529768[MHz]
X_Offset = 100[ppm]
X_Points = 32768
X_Prescans = 4
X_Resolution = 1.19959034[Hz]
X_Sweep = 39.3081761[kHz]
X_Sweep_Clipped = 31.44654088[kHz]
Irr_Domain = Proton
Irr_Freq = 500.15991521[MHz]
Irr_Offset = 5.0[ppm]
Clipped = TRUE
Scans = 1750
Total_Scans = 1750
Relaxation_Delay = 2[s]
Recvr_Gain = 58
Temp_Get = 21.5[dC]
X_90_Width = 52.78[us]
X_Acq_Time = 0.83361792[s]
X_Angle = 30[deg]
X_Atn = 9.7[dB]
X_Pulse = 17.59333333[us]
Irr_Atn_Dec = 20.948[dB]
Irr_Atn_Noe = 20.948[dB]
Irr_Noise = WALTZ
Irr_Pwidth = 92[us]
Decoupling = TRUE
Initial_Wait = 1[s]
Noe = TRUE
Noe_Time = 2[s]
Repetition_Time = 2.83361792[s]

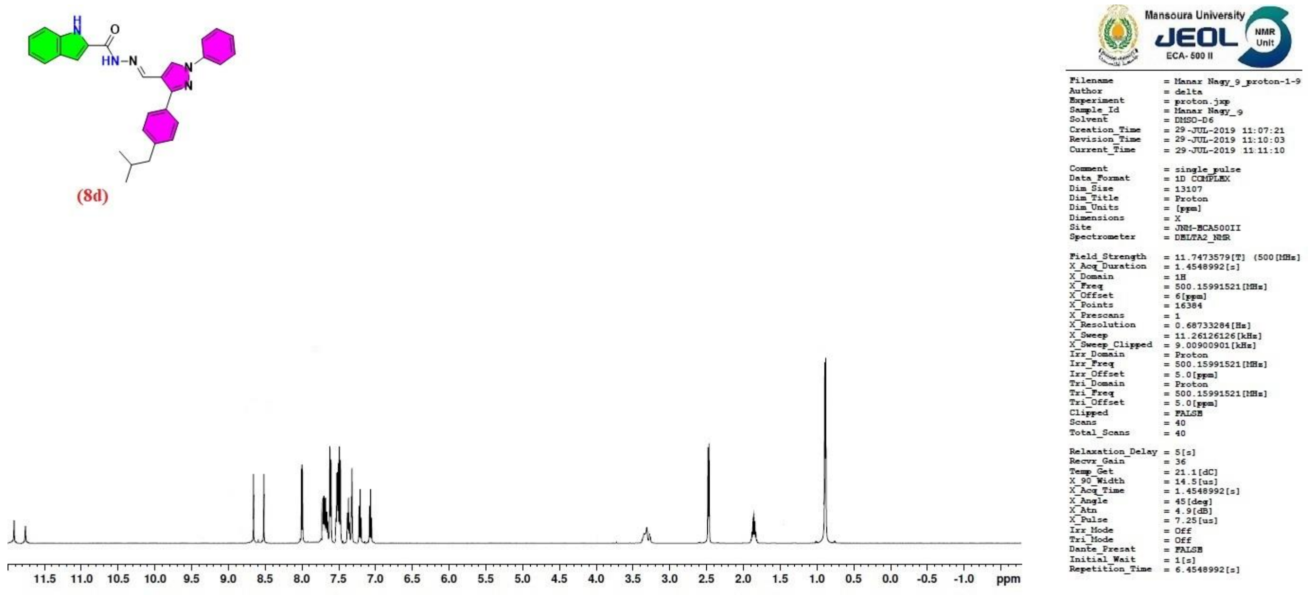
(8d)
Mansoura University
JEOL
NMR Unit
ECA- 500 II
Filename = Manar Nagy_9_proton-1-9
Author = delta
Experiment = proton.jxp
Sample_Id = Manar Nagy_9
Solvent = DMSO-D6
Creation_Time = 29-JUL-2019 11:07:21
Revision_Time = 29-JUL-2019 11:10:03
Current_Time = 29-JUL-2019 11:11:10
Comment = single_pulse
Data_Format = 1D COMPLEX
Dim_Size = 13107
Dim_Title = Proton
Dim_Units = [ppm]
Dimensions = X
Site = JNM-ECA500II
Spectrometer = DELTA2_NMR
Field_Strength = 11.7473579[T] (500[MHz])
X_Acq_Duration = 1.4548992[s]
X_Domain = 1H
X_Freq = 500.15991521[MHz]
X_Offset = 6[ppm]
X_Points = 16384
X_Prescans = 1
X_Resolution = 0.68733284[Hz]
X_Sweep = 11.26126126[kHz]
X_Sweep_Clipped = 9.00900901[kHz]
Irr_Domain = Proton
Irr_Freq = 500.15991521[MHz]
Irr_Offset = 5.0[ppm]
Tri_Domain = Proton
Tri_Freq = 500.15991521[MHz]
Tri_Offset = 5.0[ppm]
Clipped = FALSE
Scans = 40
Total_Scans = 40
Relaxation_Delay = 5[s]
Recvr_Gain = 36
Temp_Get = 21.1[dC]
X_90_Width = 14.5[us]
X_Acq_Time = 1.4548992[s]
X_Angle = 45[deg]
X_Atn = 4.9[dB]
X_Pulse = 7.25[us]
Irr_Mode = Off
Tri_Mode = Off
Dante_Presat = FALSE
Initial_Wait = 1[s]
Repetition_Time = 6.4548992[s]
11.5 11.0 10.5 10.0 9.5 9.0 8.5 8.0 7.5 7.0 6.5 6.0 5.5 5.0 4.5 4.0 3.5 3.0 2.5 2.0 1.5 1.0 0.5 0.0 -0.5 -1.0 ppm

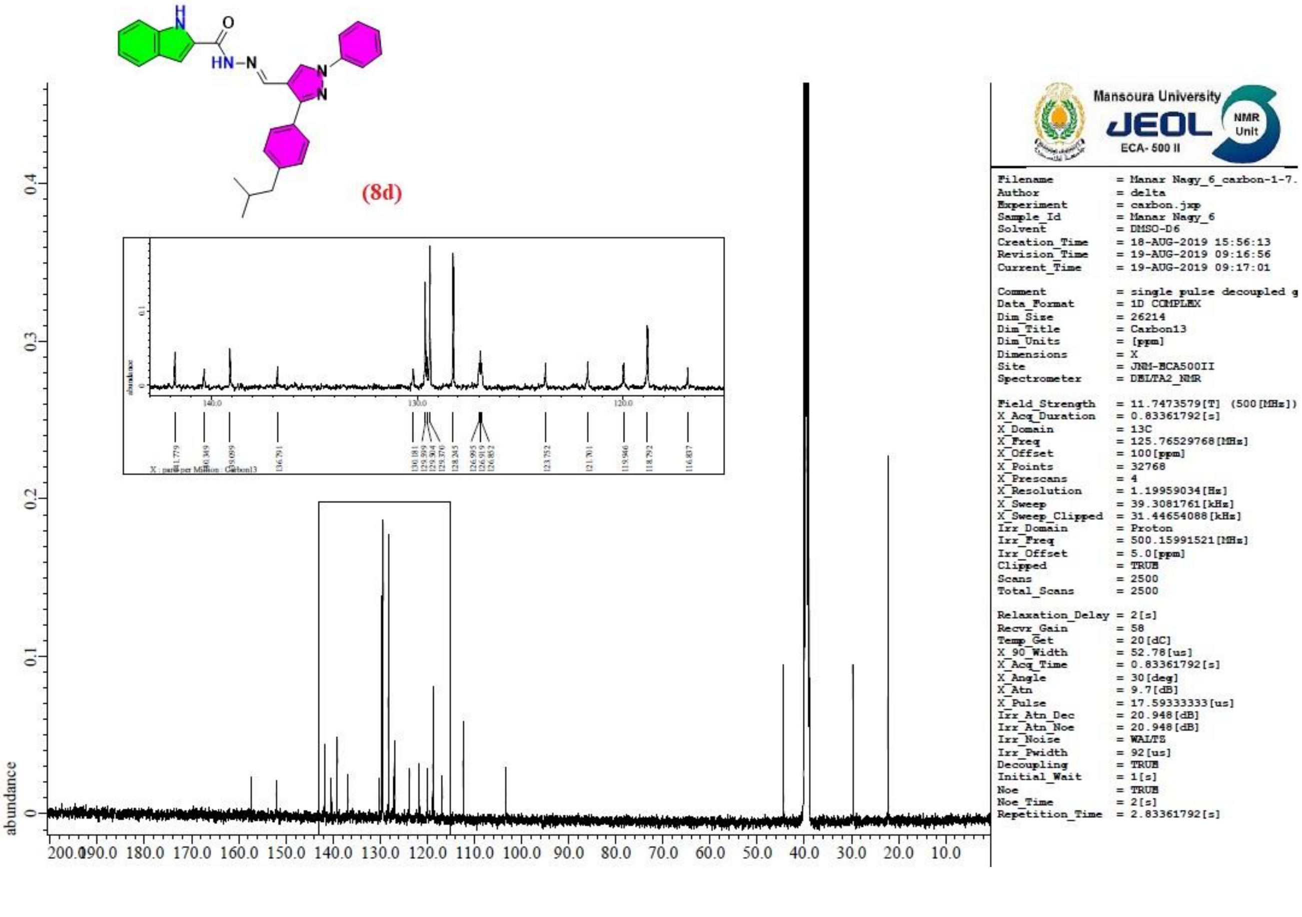
(8d)
Mansoura University
JEOL
NMR Unit
ECA- 500 II
Filename = Manar Nagy_6_carbon-1-7.
Author = delta
Experiment = carbon.jxp
Sample_Id = Manar Nagy_6
Solvent = DMSO-D6
Creation_Time = 18-AUG-2019 15:56:13
Revision_Time = 19-AUG-2019 09:16:56
Current_Time = 19-AUG-2019 09:17:01
Comment = single pulse decoupled g
Data_Format = 1D COMPLEX
Dim_Size = 26214
Dim_Title = Carbon13
Dim_Units = [ppm]
Dimensions = X
Site = JNM-ECA500II
Spectrometer = DELTA2_NMR
Field_Strength = 11.7473579[T] (500[MHz])
X_Acq_Duration = 0.83361792[s]
X_Domain = 13C
X_Freq = 125.76529768[MHz]
X_Offset = 100[ppm]
X_Points = 32768
X_Prescans = 4
X_Resolution = 1.19959034[Hz]
X_Sweep = 39.3081761[kHz]
X_Sweep_Clipped = 31.44654088[kHz]
Irr_Domain = Proton
Irr_Freq = 500.15991521[MHz]
Irr_Offset = 5.0[ppm]
Clipped = TRUE
Scans = 2500
Total_Scans = 2500
Relaxation_Delay = 2[s]
Recvr_Gain = 58
Temp_Get = 20[dC]
X_90_Width = 52.78[us]
X_Acq_Time = 0.83361792[s]
X_Angle = 30[deg]
X_Atn = 9.7[dB]
X_Pulse = 17.59333333[us]
Irr_Atn_Dec = 20.948[dB]
Irr_Atn_Noe = 20.948[dB]
Irr_Noise = WALTS
Irr_Pwidth = 92[us]
Decoupling = TRUE
Initial_Wait = 1[s]
Noe = TRUE
Noe_Time = 2[s]
Repetition_Time = 2.83361792[s]
abundance
0 0.1 0.2 0.3 0.4
200.0 190.0 180.0 170.0 160.0 150.0 140.0 130.0 120.0 110.0 100.0 90.0 80.0 70.0 60.0 50.0 40.0 30.0 20.0 10.0

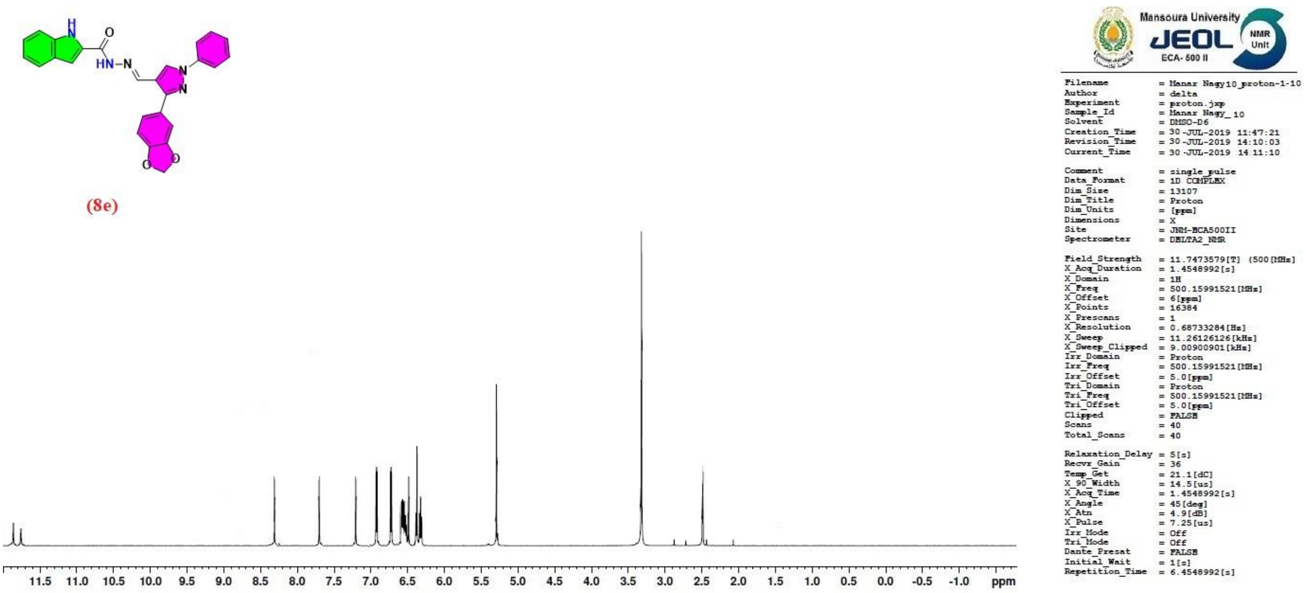
(8e)
Mansoura University
JEOL
NMR Unit
ECA- 500 II
Filename = Manar Nagy10_proton-1-10
Author = delta
Experiment = proton.jxp
Sample_Id = Manar Nagy_ 10
Solvent = DMSO-D6
Creation_Time = 30-JUL-2019 11:47:21
Revision_Time = 30-JUL-2019 14:10:03
Current_Time = 30-JUL-2019 14:11:10
Comment = single_pulse
Data_Format = 1D COMPLEX
Dim_Size = 13107
Dim_Title = Proton
Dim_Units = [ppm]
Dimensions = X
Site = JNM-ECA500II
Spectrometer = DELTA2_NMR
Field_Strength = 11.7473579[T] (500[MHz]
X_Acq_Duration = 1.4548992[s]
X_Domain = 1H
X_Freq = 500.15991521[MHz]
X_Offset = 6[ppm]
X_Points = 16384
X_Prescans = 1
X_Resolution = 0.68733284[Hz]
X_Sweep = 11.26126126[kHz]
X_Sweep_Clipped = 9.00900901[kHz]
Irr_Domain = Proton
Irr_Freq = 500.15991521[MHz]
Irr_Offset = 5.0[ppm]
Tri_Domain = Proton
Tri_Freq = 500.15991521[MHz]
Tri_Offset = 5.0[ppm]
Clipped = FALSE
Scans = 40
Total_Scans = 40
Relaxation_Delay = 5[s]
Recvr_Gain = 36
Temp_Get = 21.1[dC]
X_90_Width = 14.5[us]
X_Acq_Time = 1.4548992[s]
X_Angle = 45[deg]
X_Atn = 4.9[dB]
X_Pulse = 7.25[us]
Irr_Mode = Off
Tri_Mode = Off
Dante_Presat = FALSE
Initial_Wait = 1[s]
Repetition_Time = 6.4548992[s]
11.5 11.0 10.5 10.0 9.5 9.0 8.5 8.0 7.5 7.0 6.5 6.0 5.5 5.0 4.5 4.0 3.5 3.0 2.5 2.0 1.5 1.0 0.5 0.0 -0.5 -1.0 ppm

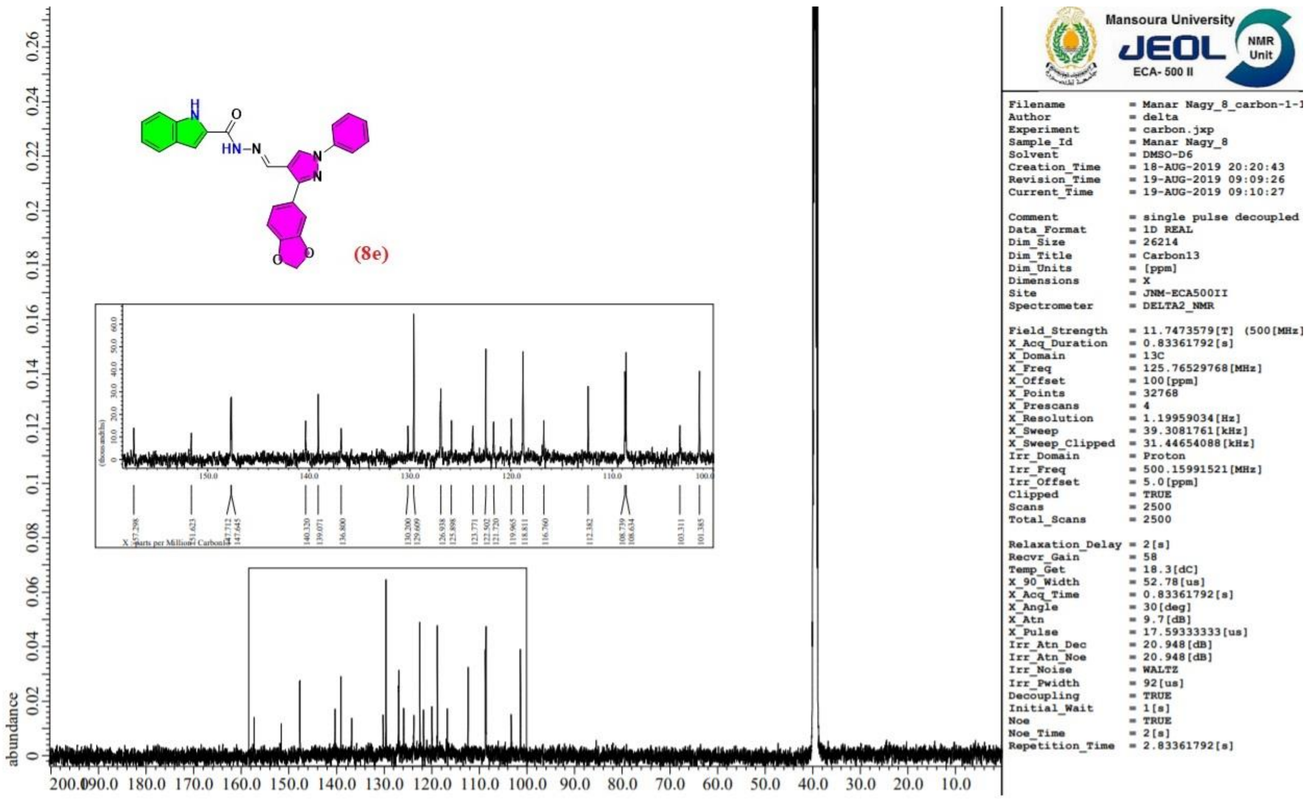
(8e)
Mansoura University
JEOL
NMR Unit
ECA- 500 II
Filename = Manar Nagy_8_carbon-1-1
Author = delta
Experiment = carbon.jxp
Sample_Id = Manar Nagy_8
Solvent = DMSO-D6
Creation_Time = 18-AUG-2019 20:20:43
Revision_Time = 19-AUG-2019 09:09:26
Current_Time = 19-AUG-2019 09:10:27
Comment = single pulse decoupled
Data_Format = 1D REAL
Dim_Size = 26214
Dim_Title = Carbon13
Dim_Units = [ppm]
Dimensions = X
Site = JNM-ECA500II
Spectrometer = DELTA2_NMR
Field_Strength = 11.7473579[T] (500[MHz]
X_Acq_Duration = 0.83361792[s]
X_Domain = 13C
X_Freq = 125.76529768[MHz]
X_Offset = 100[ppm]
X_Points = 32768
X_Prescans = 4
X_Resolution = 1.19959034[Hz]
X_Sweep = 39.3081761[kHz]
X_Sweep_Clipped = 31.44654088[kHz]
Irr_Domain = Proton
Irr_Freq = 500.15991521[MHz]
Irr_Offset = 5.0[ppm]
Clipped = TRUE
Scans = 2500
Total_Scans = 2500
Relaxation_Delay = 2[s]
Recvr_Gain = 58
Temp_Get = 18.3[dC]
X_90_Width = 52.78[us]
X_Acq_Time = 0.83361792[s]
X_Angle = 30[deg]
X_Atn = 9.7[dB]
X_Pulse = 17.59333333[us]
Irr_Atn_Dec = 20.948[dB]
Irr_Atn_Noe = 20.948[dB]
Irr_Noise = WALTZ
Irr_Pwidth = 92[us]
Decoupling = TRUE
Initial_Wait = 1[s]
Noe = TRUE
Noe_Time = 2[s]
Repetition_Time = 2.83361792[s]
abundance
X : parts per Million : Carbon13
200.0 190.0 180.0 170.0 160.0 150.0 140.0 130.0 120.0 110.0 100.0 90.0 80.0 70.0 60.0 50.0 40.0 30.0 20.0 10.0

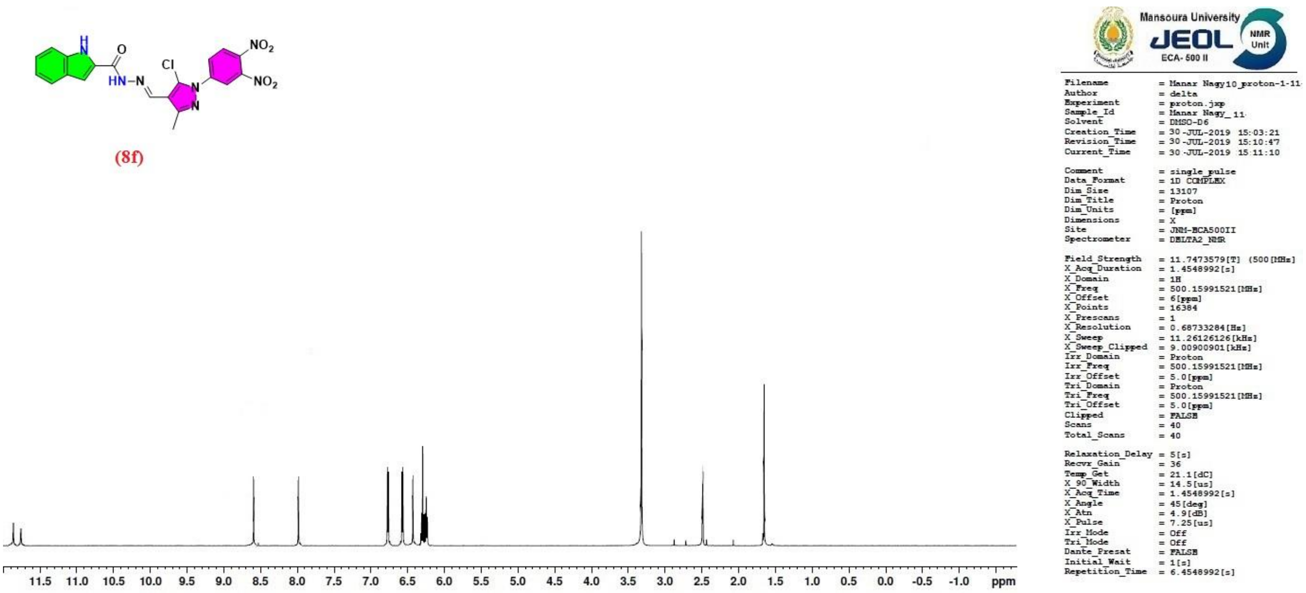

NO2
O
H
N
HN–N
Cl
N
N
NO2
(8f)
Mansoura University
JEOL
NMR Unit
ECA- 500 II
Filename = Manar Nagy10_proton-1-11
Author = delta
Experiment = proton.jxp
Sample_Id = Manar Nagy_11
Solvent = DMSO-D6
Creation_Time = 30-JUL-2019 15:03:21
Revision_Time = 30-JUL-2019 15:10:47
Current_Time = 30-JUL-2019 15:11:10
Comment = single_pulse
Data_Format = 1D COMPLEX
Dim_Size = 13107
Dim_Title = Proton
Dim_Units = [ppm]
Dimensions = X
Site = JNM-ECA500II
Spectrometer = DELTA2_NMR
Field_Strength = 11.7473579[T] (500[MHz]
X_Acq_Duration = 1.4548992[s]
X_Domain = 1H
X_Freq = 500.15991521[MHz]
X_Offset = 6[ppm]
X_Points = 16384
X_Prescans = 1
X_Resolution = 0.68733284[Hz]
X_Sweep = 11.26126126[kHz]
X_Sweep_Clipped = 9.00900901[kHz]
Irr_Domain = Proton
Irr_Freq = 500.15991521[MHz]
Irr_Offset = 5.0[ppm]
Tri_Domain = Proton
Tri_Freq = 500.15991521[MHz]
Tri_Offset = 5.0[ppm]
Clipped = FALSE
Scans = 40
Total_Scans = 40
Relaxation_Delay = 5[s]
Recvr_Gain = 36
Temp_Get = 21.1[dC]
X_90_Width = 14.5[us]
X_Acq_Time = 1.4548992[s]
X_Angle = 45[deg]
X_Atn = 4.9[dB]
X_Pulse = 7.25[us]
Irr_Mode = Off
Tri_Mode = Off
Dante_Presat = FALSE
Initial_Wait = 1[s]
Repetition_Time = 6.4548992[s]
11.5 11.0 10.5 10.0 9.5 9.0 8.5 8.0 7.5 7.0 6.5 6.0 5.5 5.0 4.5 4.0 3.5 3.0 2.5 2.0 1.5 1.0 0.5 0.0 -0.5 -1.0 ppm

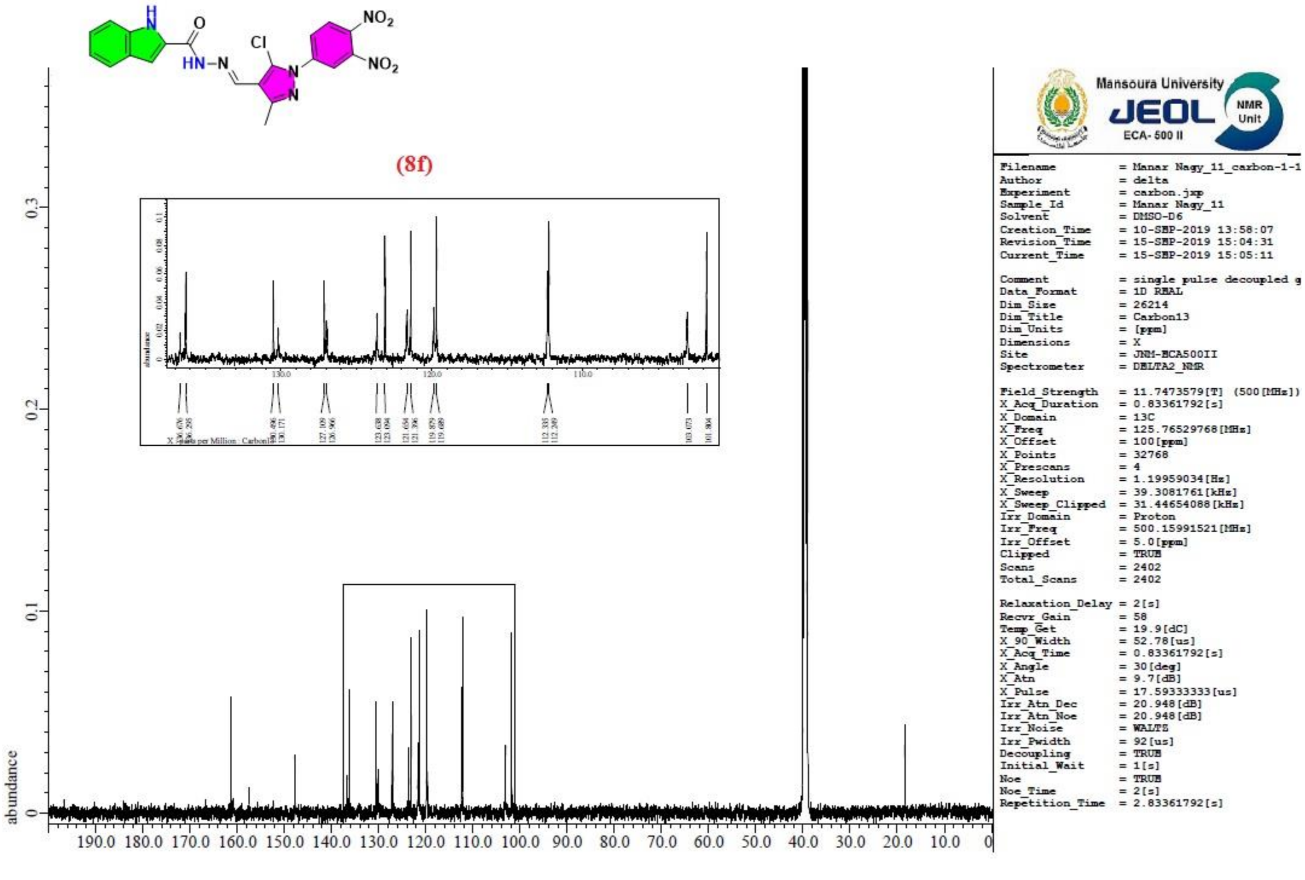

H
N
O
HN–N
Cl
N
N
NO2
NO2
(8f)
Mansoura University
JEOL
NMR Unit
ECA- 500 II
Filename = Manar Nagy_11_carbon-1-1
Author = delta
Experiment = carbon.jxp
Sample_Id = Manar Nagy_11
Solvent = DMSO-D6
Creation_Time = 10-SEP-2019 13:58:07
Revision_Time = 15-SEP-2019 15:04:31
Current_Time = 15-SEP-2019 15:05:11
Comment = single pulse decoupled g
Data_Format = 1D REAL
Dim_Size = 26214
Dim_Title = Carbon13
Dim_Units = [ppm]
Dimensions = X
Site = JNM-ECA500II
Spectrometer = DELTA2_NMR
Field_Strength = 11.7473579[T] (500[MHz])
X_Acq_Duration = 0.83361792[s]
X_Domain = 13C
X_Freq = 125.76529768[MHz]
X_Offset = 100[ppm]
X_Points = 32768
X_Prescans = 4
X_Resolution = 1.19959034[Hz]
X_Sweep = 39.3081761[kHz]
X_Sweep_Clipped = 31.44654088[kHz]
Irr_Domain = Proton
Irr_Freq = 500.15991521[MHz]
Irr_Offset = 5.0[ppm]
Clipped = TRUE
Scans = 2402
Total_Scans = 2402
Relaxation_Delay = 2[s]
Recvr_Gain = 58
Temp_Get = 19.9[dC]
X_90_Width = 52.78[us]
X_Acq_Time = 0.83361792[s]
X_Angle = 30[deg]
X_Atn = 9.7[dB]
X_Pulse = 17.59333333[us]
Irr_Atn_Dec = 20.948[dB]
Irr_Atn_Noe = 20.948[dB]
Irr_Noise = WALTZ
Irr_Pwidth = 92[us]
Decoupling = TRUE
Initial_Wait = 1[s]
Noe = TRUE
Noe_Time = 2[s]
Repetition_Time = 2.83361792[s]
abundance
0 0.1 0.2 0.3
190.0 180.0 170.0 160.0 150.0 140.0 130.0 120.0 110.0 100.0 90.0 80.0 70.0 60.0 50.0 40.0 30.0 20.0 10.0 0
130.0 120.0 110.0
X : parts per Million : Carbon13
